# Mulberry Diels–Alder-type adducts: isolation, structure, bioactivity, and synthesis

**DOI:** 10.1007/s13659-022-00355-y

**Published:** 2022-09-02

**Authors:** Si-Yuan Luo, Jun-Yu Zhu, Ming-Feng Zou, Sheng Yin, Gui-Hua Tang

**Affiliations:** School of Pharmaceutical Sciences, Sun Yat-Sen University, Guangzhou, Guangdong 510006 People’s Republic of China

**Keywords:** Mulberry Diels–Alder-type adducts, MDAAs, Natural products, Bioactivity, Synthesis

## Abstract

Mulberry Diels–Alder-type adducts (MDAAs) are unique phenolic natural products biosynthetically derived from the intermolecular [4 + 2]-cycloaddition of dienophiles (mainly chalcones) and dehydroprenylphenol dienes, which are exclusively distributed in moraceous plants. A total of 166 MDAAs with diverse skeletons have been isolated and identified since 1980. Structurally, the classic MDAAs characterized by the chalcone-skeleton dienophiles can be divided into eight groups (Types A − H), while others with non-chalcone dienophiles or some variations of classic MDAAs are non-classic MDAAs (Type I). These compounds have attracted significant attention of natural products and synthetic chemists due to their complex architectures, remarkable biological activities, and synthetic challenges. The present review provides a comprehensive summary of the structural properties, bioactivities, and syntheses of MDAAs. Cited references were collected between 1980 and 2021 from the SciFinder, Web of Science, and China National Knowledge Internet (CNKI).

**Graphical Abstract:**

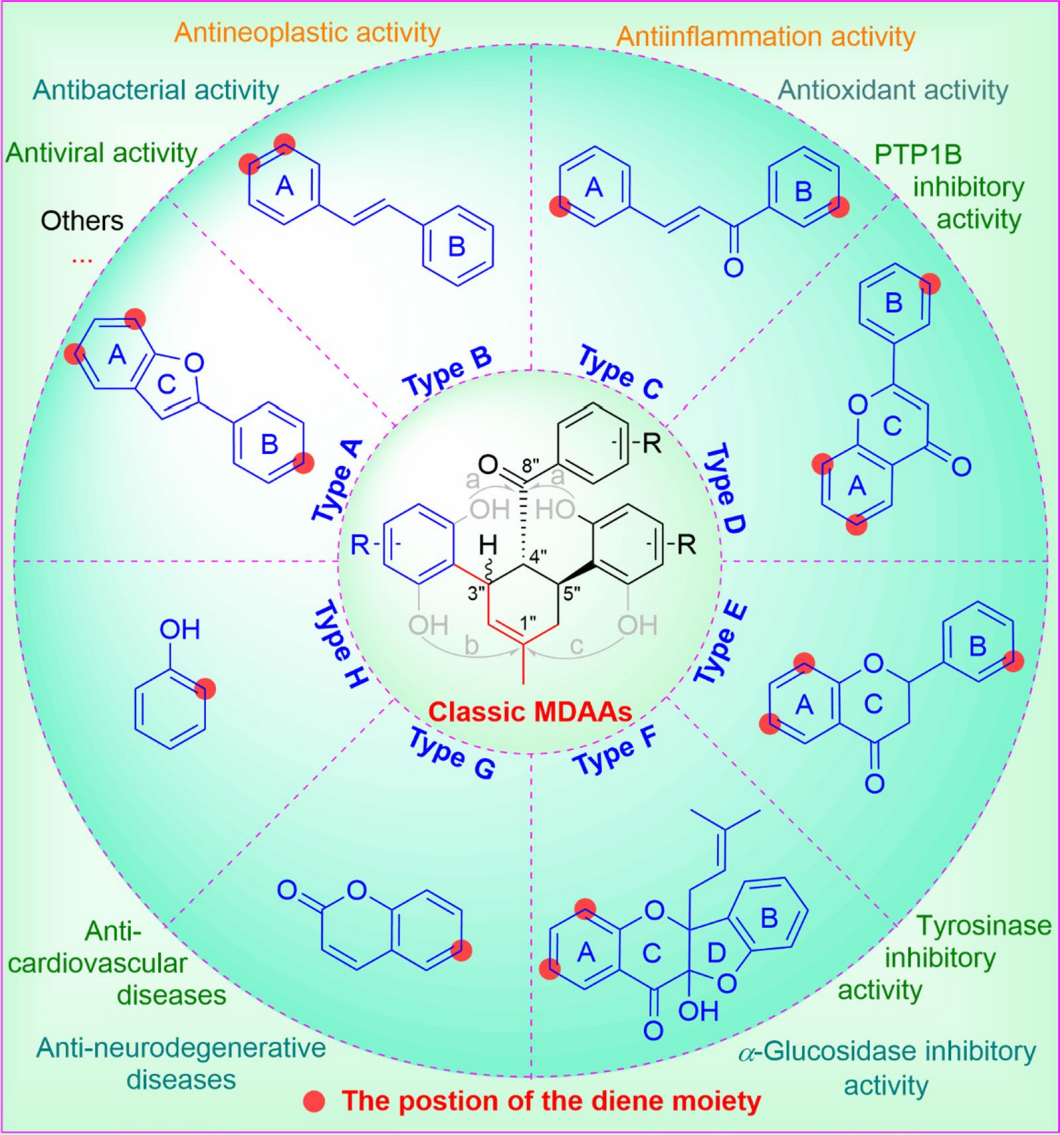

## Introduction to mulberry Diels–Alder-type adducts (MDAAs)

**Scheme 1:**
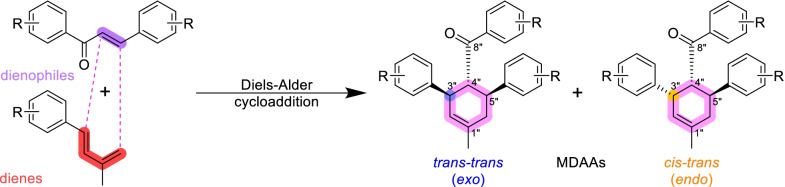
The biosynthesis pathway of classic MDAAs

Mulberry Diels–Alder-type adducts (MDAAs), characteristic constituents of mulberry trees (*Morus* plants of the family Moraceae), are a group of structurally unique natural phenolic compounds biosynthetically derived from the intermolecular [4 + 2]-cycloaddition of dienophiles (mainly chalcones) and dehydroprenylphenol dienes (Scheme 1). Chalcomoracin^.^and kuwanons G and H (albanins F and G), the first representatives of MDAAs, were almost simultaneously reported from the well-known mulberry tree (*Morus alba* L.) by the two groups of Nomura and Takasugi in 1980 [1–5]. A total of 166 MDAAs have been obtained and characterized over the past four decades. MDAAs are not widely distributed in the plants of the family Moraceae, and until now, they have only found in seven genera (21 species) of this family, including *Morus* [13 species, *M. alba* (including the variant *M. alba* var. *shalun*), *M. australis*, *M. bombycis*, *M. cathayana*, *M. insignis*, *M. lhou*, *M. macroura*, *M. mesozygia*, *M. mongolica* (including *M. yunanensis*, a species revised as the present Latin name), *M. multicaulis*, *M. nigra*, *M. notabilis*, and *M. wittiorum*], *Artocarpus* (two species, *A. heterophyllus* and *A. integer*), *Sorocea* (two species, *S. bonplandii* and *S. ilicifolia*), *Brosimopsis* (one species, *B. oblongifolia*), *Brosimum* (one species, *Brosimum rubescens*), *Chlorophora* (one species, *C. regia*), and *Dorstenia* (one species, *D. barteri*). Among these 21 species, *M. alba*, *M. mongolica*, and *M. macroura* were the top three species rich in different MDAAs. Natural MDAAs were demonstrated to occur generally in root barks, stem barks, roots, stems or twigs, leaves, and callus cultures.

A mini-review (in Chinese) on MDAAs and several comprehensive reviews on secondary metabolites from moraceous plants especially the *Morus* genus covered a limited number of MDAAs, but none has provided a complete and in-depth analysis of this group of natural products [6–10]. In this review, it provides a comprehensive summary of the structural classification, distribution, and biological functions of 166 naturally occurring MDAAs. The total synthetic investigations towards this family of compounds by various chemistry research groups are summarized for the first time.

## Structural characteristics and classification of MDAAs

**Fig. 1:**
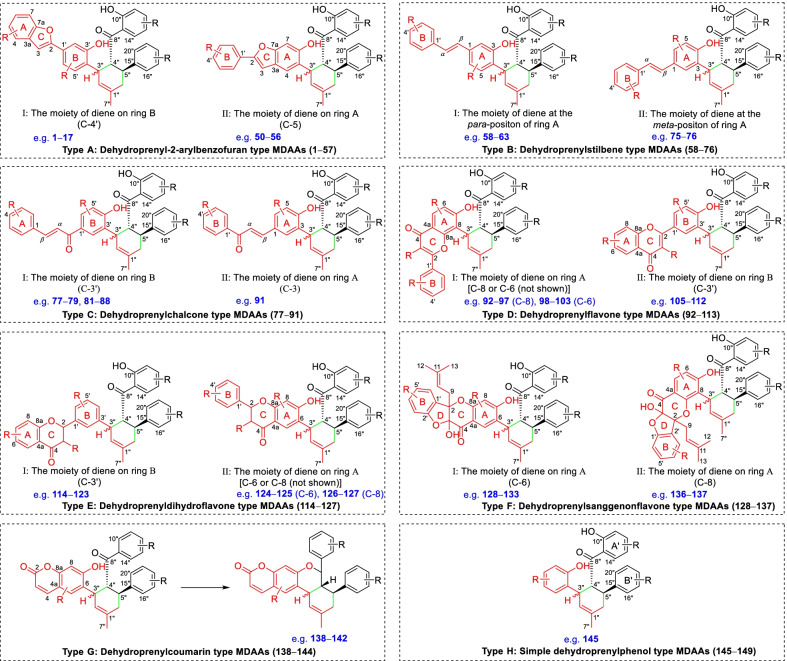
Structures of eight important groups of classic MDAAs (Types A − H)

According to the structural characteristics, MDAAs can be divided into classic and non-classic types. Structurally, classic MDAAs share the same chalcone-skeleton dienophiles but differ in the dehydroprenylphenol dienes. In light of the structural types of dehydroprenylphenol dienes, classic MDAAs (Fig. 1) can be further classified into dehydroprenyl-2-arylbenzofuran type (Type A), dehydroprenylstilbene type (Type B), dehydroprenylchalcone type (Type C), dehydroprenylflavone type (Type D), dehydroprenyldihydroflavone type (Type E), dehydroprenylsanggenonflavone type (Type F), dehydroprenylcoumarin type (Type G), and simple or other dehydroprenylphenol type (Type H). Non-classic MDAAs (Type I) is considered as a kind of Diels–Alder adducts derived from cycloaddition of non-chalcone dienophiles and dehydroprenylphenol dienes or as variations of some classic MDAAs. All MDAAs are phenolic natural products, and the presence of adjacent phenolic hydroxyl groups has allowed different natural modifications of the ketone as well as of newly formed methylcyclohexene ring, resulting in compounds with complex structural features. In addition, the presence of intact or modified prenyl groups in the moiety of dienophiles or dienes also leads to a diversity of MDAAs.

The occurrence and distribution details of 166 MDAAs together with their specific rotation values are summarized in Table 1.

**Table 1:** MDAAs isolated from moraceous plants

Compound name (number)^a^	[*α*]	Species^b^
Type A: Dehydroprenyl-2-arylbenzofuran type MDAAs 57 compounds (**1** − **57**) distributed in 14 species: *C. regia*, *M. alba* (including *M. alba* var. *shalun*), *M. australis*, *M. bombycis*, *M. insignis*, *M. lhou*, *M. macroura*, *M. mesozygia*, *M. mongolica* (syn. *M. yunanensis*), *M. nigra*, *M. notabilis*, *M. wittiorum*, *S. bonplandii*, and *S. ilicifolia*		
Mulberrofuran J (**1**)^#^	− 341 [11]	*M. alba* [12–15]; *M. australis* [16]; *M. lhou* [11]; *M. macroura* [17–19]; *M. mongolica* [20]; *M. nigra* [21]; *M. wittiorum* [22]; *M. yunanensis* [23, 24]
Mongolicin F (**2**)^#^	− 283 [25]	*M. macroura* [19]; *M. mongolica* [25]; *M. wittiorum* [22]; *M. yunanensis* [23]
Albasin A (**3**)	− 217.7 [14]	*M. alba* [14]
Morushalunin (**4**)	− 374.4 [26]	*M. alba* var. *shalun* [26]
Chalcomoracin (**5**)^#,##^	+ 194 [4]	*C. regia* [27]; *M. alba* [4, 14, 28–32]; *M. alba* var. *shalun* [26]; *M. australis* [16]; *M. bombycis* [33]; *M. macroura* [19, 34]; *M. mongolica* [25]; *M. mesozygia* [35]; *M. nigra* [36, 37]; *M. notabilis* [38, 39]; *M. wittiorum* [22]; *M. yunanensis* [23]; *S. bonplandii* [40]; *S. ilicifolia* [41]
Mulberrofuran C (**6**)^#^	+ 153 [42]	*M. alba* [13–15, 43–45]; *M. bombycis* [33, 42]; *M. macroura* [18]
Morbilisin F (**7**)	+ 145.3 [39]	*M. notabilis* [39]
Albasin B (**8**)	+ 440.0 [14]	*M. alba* [14, 46]
Mulberrofuran E (**9**)	+ 302 [47]	*M. alba* [14, 31, 47]; *M. australis* [16]; *M. macroura* [48]; *M. wittiorum* [49]; *M. yunanensis* [23, 50]
Morusalisin C (**10**)	+ 99.7 [51]	*M. alba* [51]
Mulberrofuran U (**11**)	+ 128 [52]	*M. insignis* [52]
Mulberrofuran T (**12**)	+ 139 [53]	*M. alba* [53]; *M. mongolica* [25]
Mulberrofuran O (**13**)	+ 196 [54]	*M. alba* [54]; *M. macroura* [19]; *M. wittiorum* [49]; *M. yunanensis* [23, 24]; *S. ilicifolia* [55]
Morbilisin G (**14**)	+ 217.0 [39]	*M. notabilis* [39]
Morbilisin H (**15**)	+ 96.1 [39]	*M. notabilis* [39]
Morusalisin D (**16**)	+ 85.5 [51]	*M. alba* [51]
Morusalisin E (**17**)	+ 89.7 [51]	*M. alba* [51]
Wittiorumin F (**18**)	+ 299.1 [22]	*M. macroura* [48]; *M. wittiorum* [22]
Morbilisin E (**19**)	+ 244.2 [39]	*M. notabilis* [39]
Inethermulberrofuran C (**20**)	+ 232.0 [15]	*M. alba* [15]
Macrourin I (**21**)	+ 305.6 [48]	*M. macroura* [48]
Macrourin J (**22**)	+ 302.3 [48]	*M. macroura* [48]
Yunanensin C (**23**)	+ 439 [23]	*M. yunanensis* [23]
Mongolicin C (**24**)	+ 160 [25]	*M. alba* [13, 14, 46]; *M. australis* [16]; *M. mongolica* [25]; *M. yunanensis* [23]
Australisine B (**25**)	+ 191.3 [16]	*M. australis* [16]
Macrourin G (**26**)	+ 175.1 [19]	*M. alba* [46]; *M. macroura* [19]
Morusalbin A (**27**)	+ 48.6 [46]	*M. alba* [46]
Mulberrofuran I (**28**)	+ 212.2 [56]	*M. bombycis* [56]
Mulberrofuran S (**29**)	+ 166 [57]	*M. alba* [57]
Mulberrofuran Q (**30**)	+ 82.4 [58]	*M. alba* [58–62]; *M. australis* [16]; *M. mongolica* [20]; *M. yunanensis* [23, 24]
Mulberrofuran G (Albanol A, **31**)	+ 137.2 [63]; + 546 [64]; + 515 [65]	*M. alba* [12–15, 44, 46, 59, 61–63, 66–69]; *M. australis* [16, 70]; *M. bombycis* [71–73]; *M. lhou* [64, 65]; *M. macroura* [74]; *M. mongolica* [25]; *M. nigra* [21, 75, 76]
Mulberrofuran F (**32**)	+ 513 [64]	*M. alba* [14]; *M. australis* [16]; *M. lhou* [64, 65]; *M. macroura* [48]; *M. mongolica* [25]; *M. wittiorum* [49]; *M. yunanensis* [23]
Mulberrofuran F_1_ (**33**)	+ 412.3 [29]	*M. alba* [29]
Mongolicin A (**34**)	+ 605 [25]	*M. mongolica* [25]
Morbilisin C (**35**)	+ 538.5 [39]	*M. notabilis* [39]
Morbilisin D (**36**)	+ 510.8 [39]	*M. notabilis* [39]
Morusalisin B (**37**)	+ 82.5 [51]	*M. alba* [51]
Yunanensin E (**38**)	+ 674.8 [23]	*M. yunanensis* [23]
Morbilisin B (**39**)	+ 527.3 [39]	*M. notabilis* [39]
Mulberrofuran K (**40**)	+ 425 [54]	*M. alba* [14, 15, 46, 54, 62]; *M. alba* var. *shalun* [77]; *M. bombycis* [73]; *M. macroura* [48, 74]; *M. yunanensis* [23, 50]; *S. bonplandii* [40]
Morbilisin A (**41**)	+ 539.5 [39]	*M. notabilis* [39]
Morusalbin B (**42**)	+ 237.0 [46]	*M. alba* [46]
Yunanensin D (**43**)	+ 161.5 [78]	*M. yunanensis* [23, 78]
Yunanensin A (**44**)	+ 12.0 [79]	*M. alba* [46]; *M. yunanensis* [23, 79]
Mulberrofuran P (**45**)	+ 19.5 [80]	*M. alba* [80]
Albanol B (**46**)	+ 118 [50]	*M. alba* [46, 62, 63, 67, 81, 82]; *M. mongolica* [25, 83]; *M. nigra* [75]; *M. yunanensis* [23, 50]
Sorocenol C (**47**)	0 [40]	*S. bonplandii* [40]
Sorocenol D (**48**)	0 [40]	*S. bonplandii* [40]
Morusalbin C (**49**)	0 [46]	*M. alba* [46]
Albafuran C (**50**)	− 302 [84]	*M. alba* [15, 82, 84]; *M. lhou* [11]; *M. macroura* [18, 48, 85]; *M. nigra* [76]; *M. wittiorum* [49]
Guangsangon J (**51**)	− 419.7 [17]	*M. macroura* [17, 19]; *M. yunanensis* [23]
Guangsangon A (**52**)	− 408.5 [85]	*M. macroura* [85]
Morusalisin F (**53**)	− 82.5 [51]	*M. alba* [51]
Yunanensin B (**54**)	− 183.2 [78]	*M. yunanensis* [23, 78]
Australisine C (**55**)	+ 340 [16]	*M. australis* [16]
Guangsangon E (**56**)	+ 139.7 [85]	*M. alba* var. *shalun* [26]; *M. macroura* [19, 34, 85]
Isomulberrofuran G (**57**)	+ 514.8 [66]	*M. alba* [15, 66]
Type B: Dehydroprenylstilbene type MDAAs 19 compounds (**58** − **76**) distributed in ten species: *C. regia*, *M. alba*, *M. bombycis*, *M. cathayana*, *M. lhou*, *M. macroura*, *M. mongolica* (syn. *M. yunanensis*), *M. wittiorum*, *S. bonplandii*, and *S. ilicifolia*		
Kuwanon X (**58**)^##^	− 332 [11]	*M. lhou* [11]; *M. macroura* [18, 85]; *M. yunanensis* [23, 24]
Macrourin F (**59**)	− 925.0 [19]	*M. macroura* [19]
Macrourin E (**60**)	− 525.0 [19]	*M. macroura* [19]
Kuwanon Y (**61**)^#,##^	+ 172 [86]	*M. alba* [86]; *M. macroura* [18, 85]
Kuwanol E (**62**)^##^	+ 171 [53]	*C. regia* [27]; *M. alba* [53]; *M. macroura* [19]; *S. bonplandii* [40, 87]; *S. ilicifolia* [41]
Sorocein M (**63**)	+ 112 [55]	*S. ilicifolia* [55]
Kuwanol A (**64**)^##^	+ 577 [88]	*M. alba* [14, 15, 62]; *M. bombycis* [88]; *M. yunanensis* [23]
Sorocein I (**65**)	+ 331 [89]	*M. macroura* [34, 48]; *S. ilicifolia* [89]
Sorocein L (**66**)	+ 290 [55]	*S. ilicifolia* [55]
Morusalbin D (**67**)	+ 446.5 [46]	*M. alba* [46]
Sorocein A (**68**)	+ 477 [90]	*M. macroura* [18, 19]; *M. wittiorum* [49]; *M. yunanensis* [23]; *S. bonplandii* [90]; *S. ilicifolia* [41]
Sorocenol E (**69**)	0 [40]	*S. bonplandii* [40]
Cathayanon C (**70**)	+ 11.4 [91]	*M. cathayana* [91]
Cathayanon D (**71**)	− 5.7 [91]	*M. cathayana* [91]
Kuwanol B (**72**)	+ 103.5 [88]	*M. bombycis* [88]
Kuwanon Z (**73**)	+ 40.5 [86]	*M. alba* [86]
Sorocenol F (**74**)	+ 20 [40]	*S. bonplandii* [40]
Guangsangon B (**75**)	− 394.7 [85]	*M. macroura* [85]; *M. yunanensis* [23, 24]
Kuwanon P (**76**)	− 509 [92]	*M. lhou* [11, 92]; *M. macroura* [18, 85]; *M. yunanensis* [23, 24]
Type C: Dehydroprenylchalcone type MDAAs 15 compounds (**77** − **91**) distributed in 12 species: *A. heterophyllus*, *A. integer*, *B. oblongifolia*, *D. barteri*, *M. alba* (including *M. alba* var. *shalun*), *M. bombycis*, *M. macroura*, *M. mongolica*, *M. nigra*, *M. notabilis*, *S. bonplandii*, and *S. ilicifolia*		
Brosimone B (**77**)^##^	− 447 [93]	*B. oblongifolia* [93]
Kuwanon I (**78**)^#,##^	− 454 [94]	*M. alba* [94]
Dorstenone (**79**)^#^	− 371 [95]	*D. barteri* [95]
Brosimone A (**80**)^##^	− 711 [96]	*B. oblongifolia* [96]
Kuwanon J (**81**)^#,##^	+ 85 [28]	*A. integer* [97]; *M. alba* [14, 28, 29]; *M. alba* var. *shalun* [26]; *M. bombycis* [71, 73, 98]; *M. macroura* [17, 18, 48]; *M. mongolica* [20, 83]; *M. nigra* [36]; *M. notabilis* [38]; *S. bonplandii* [40, 99]; *S. ilicifolia* [55]
Kuwanon J 2,4,10"-trimethyl ether (**82**)	+ 102.5 [100]	*M. alba* [100]
Kuwanon Q (**83**)	+ 161 [47]	*M. alba* [47]; *M. bombycis* [71]
Artonin C (**84**)	+ 20 [101]	*A. heterophyllus* [101]
Artonin X (**85**)	+ 12 [102]	*A. heterophyllus* [102]
Kuwanon R (**86**)	+ 56 [47]	*A. heterophyllus* [97, 102]; *M. alba* [14, 47, 100]; *M. bombycis* [71, 73, 98]
Kuwanon V (**87**)^#^	+ 145 [47]	*M. alba* [47]; *M. bombycis* [71, 73, 98, 103]
Artonin D (**88**)	+ 77 [101]	*A. heterophyllus* [101, 102]; *S. bonplandii* [87, 99]
Mongolicin G (**89**)	+ 166 [104]	*M. mongolica* [104]
Sorocein B (**90**)	+ 962 [90]	*S. bonplandii* [87, 90]; *S. ilicifolia* [41]
Guangsangon C (**91**)	–412.8 [85]	*M. macroura* [85]
Type D: Dehydroprenylflavone type MDAAs 22 compounds (**92** − **113**) distributed in 13 species: *A. heterophyllus*, *A. integer*, *B. oblongifolia*, *M. alba*, *M. australis*, *M. bombycis*, *M. lhou*, *M. macroura*, *M. mesozygia*, *M. mongolica* (syn. *M. yunanensis*), *M. multicaulis*, *M. nigra*, and *M. wittiorum*		
Kuwanon G (Albanin F, Moracenin B, **92**)^##^	− 534 [1]; − 529 [3]	*M. alba* [1, 3, 5, 15, 61, 62, 67, 82, 105–109]; *M. australis* [16, 70]; *M. bombycis* [33]; *M. lhou* [110]; *M. mongolica* [20]; *M. nigra* [21, 37, 76]; *M. yunanensis* [23, 24]
Kuwanon H (Albanin G, Moracenin A, **93**)^##^	− 536 [2]; − 455 [3]	*M. alba* [2, 3, 5, 15, 61, 62, 82, 108, 111, 112]; *M. bombycis* [33]; *M. lhou* [110]; *M. mongolica* [25]; *M. nigra* [21, 37]; *M. yunanensis* [23, 50]
Mongolicin D (**94**)	− 227 [25]	*M. mongolica* [25]
Moracenin D (**95**)	− 419 [105]	*M. alba* [61, 69, 105]; *M. australis* [70]
Moracenin E (**96**)	n.m^c^	*M. alba* [61]
Kuwanon W (**97**)	− 440 [113]	*M. lhou* [113]
Brosimone D (**98**)	− 204 [93]	*B. oblongifolia* [93]
Multicaulisin (**99**)	− 136 [114]	*M. alba* [111]; *M. multicaulis* [114]
Mesozygin B (**100**)	+ 139.6 [35]	*M. mesozygia* [35]
Artonin I (**101**)^##^	+ 95 [115]	*A. heterophyllus* [115]; *M. alba* [13]; *M. mesozygia* [35, 116]
Deoxyartonin I (**102**)^##^	+ 177 [117]	*A. integer* [97]
Mesozygin C (**103**)	+ 85.7 [35]	*M. mesozygia* [35]
Mesozygin A (**104**)	+ 343.2 [35]	*M. mesozygia* [35]
Guangsangon G (**105**)	− 469.1 [17]	*M. macroura* [17]
Guangsangon I (**106**)	− 470.5 [17]	*M. macroura* [17]
Wittiorumin A (**107**)	− 415.3 [22]	*M. wittiorum* [22]
Wittiorumin B (**108**)	− 442.6 [22]	*M. wittiorum* [22]
Kuwanon K (**109**)	− 218 [118]	*M. alba* [82, 118]; *M. lhou* [110]
Kuwanon N (**110**)	− 188 [110]	*M. australis* [70]; *M. lhou* [110]
Guangsangon O (**111**)	+ 212.1 [119]	*M. macroura* [119]
Wittiorumin C (**112**)	+ 420.2 [22]	*M. wittiorum* [22]
Australisine A (**113**)	+ 523 [16]	*M. australis* [16]
Type E: Dehydroprenyldihydroflavone type MDAAs 14 compounds (**114** − **127**) distributed in seven species: *M. alba*, *M. australis*, *M. lhou*, *M. macroura*, *M. mongolica* (syn. *M. yunanensis*), *M. nigra*, and *M. wittiorum*		
Guangsangon M (**114**)	− 276.5 [74]	*M. macroura* [74]
Guangsangon N (**115**)	− 335.3 [74]	*M. macroura* [74]
Wittiorumin D (**116**)	− 575.0 [22]	*M. wittiorum* [22]
Wittiorumin E (**117**)	− 296.5 [22]	*M. wittiorum* [22]
Kuwanon L (**118**)	− 227 [118]	*M. alba* [43, 61, 62, 69, 82, 118, 120]; *M. australis* [70]; *M. mongolica* [20]; *M. nigra* [37]
Kuwanon O (**119**)	− 243 [110]	*M. alba* [62, 107]; *M. australis* [70]; *M. lhou* [110]; *M. mongolica* [25]; *M. yunanensis* [23]
Guangsangon D (**120**)	− 108.3 [85]	*M. macroura* [85]
Guangsangon H (**121**)	− 127.9 [17]	*M. macroura* [17]
Guangsangon K (**122**)	− 178.5 [74]	*M. macroura* [74]
Guangsangon F (**123**)	− 112.1 [74]	*M. macroura* [74]
Sanggenon G (**124**)	− 277 [121]	*M. alba* [43–45, 61, 69, 111, 120, 121]; *M. mongolica* [122]
Sanggenon T (**125**)	− 194 [123]	*M. alba* [123]; *M. australis* [70]; *M. mongolica* [122]
Sanggenol M (**126**)	− 126 [122]	*M. mongolica* [122]; *M. yunanensis* [23, 24]
Wittiorumin G (**127**)	+ 87.3 [49]	*M. wittiorum* [49]
Type F: Dehydroprenylsanggenonflavone type MDAAs 10 compounds (**128** − **137**) distributed in seven species: *C. regia*, *M. alba*, *M. bombycis*, *M. cathayana*, *M. mongolica*, *M. nigra*, and *S. bonplandii*		
Sanggenon D (**128**)	− 145 [124]	*M. alba* [81, 120, 124]; *M. cathayana* [125]; *M. mongolica* [122]
Sanggenon E (**129**)	− 86 [126]	*M. alba* [120, 126, 127]; *M. nigra* [128]
Sanggenon C (**130**)^##^	+ 304 [129]	*C. regia* [27]; *M. alba* [43, 69, 120, 127, 129]; *M. bombycis* [71]; *M. cathayana* [91, 125, 130]; *M. mongolica* [83, 122]; *M. nigra* [36]
Sanggenon O (**131**)^##^	− 64 [131]	*M. alba* [69, 120, 127, 131]; *M. bombycis* [71]; *M. cathayana* [130]; *M. mongolica* [83]; *M. nigra* [36]
Sanggenon P (Sorocein H, **132**)	+ 215 [126]; + 235 [99]	*M. alba* [126]; *M. nigra* [36]; *S. bonplandii* [99]
Sanggenol J (**133**)	+ 98 [125]	*M. cathayana* [125]
Sorocein C (**134**)	+ 427 [99]	*S. bonplandii* [99]; *S. ilicifolia* [41]
Cathayanon E (**135**)	+ 189.1 [91]	*M. cathayana* [91]
Cathayanon A (**136**)	− 193.9 [130]	*M. cathayana* [130]
Cathayanon B (**137**)	− 733.7 [130]	*M. cathayana* [130]
Type G: Dehydroprenylcoumarin MDAAs Seven compounds (**138** − **144**) distributed in one species: B.* rubescens		
Palodesangren A (**138**)	− 11.3 [132]	B.* rubescens [132]
Palodesangren B (**139**)^#^	+ 7.7 [132]	B.* rubescens [132]
Palodesangren C (**140**)	+ 5.6 [132]	B.* rubescens [132]
Palodesangren D (**141**)^#^	+ 2.5 [132]	B.* rubescens [132]
Palodesangren E (**142**)	− 5.2 [132]	B.* rubescens [132]
Palodesagretin I (**143**)	+ 7.1 [133]	B.* rubescens [133]
Palodesagretin II (**144**)	+ 12.5 [133]	B.* rubescens [133]
Type H: Simple or other dehydroprenylphenol type MDAAs Six compounds (**145** − **150**) distributed in five species: *M. alba*, *M. macroura*, *M. mongolica* (syn. *M. yunanensis*), *M. nigra*, and *S. bonplandii*		
Guangsangon L (**145**)	− 389.3 [74]	*M. macroura* [74]; *M. yunanensis* [23]
Soroceal B (**146**)	+ 137.5 [13]	*M. alba* [13]
Soroceal (**147**)	+ 365 [90]	*M. nigra* [128]; *S. bonplandii* [90]
Morusalbanol A (**148**)^#^	+ 163 [134]	*M. alba* [134]
Sorocenol B (**149**)^##^	+ 560.4 [87]	*S. bonplandii* [87]
Sanggenon Q (**150**)	+ 111 [83]	*M. mongolica* [83]
Type I: Non-classic MDAAs 16 compounds (**151** − **166**) distributed in four species: *M. alba*, *M. bombycis*, *M. lhou*, and *M. mongolica*		
Kuwanon M (**151**)	− 2.0 [135]	*M. alba* [61]; *M. lhou* [135]
Mongolicin E (**152**)	+ 119 [25]	*M. mongolica* [25]
Dimoracin (**153**)	− 5 [136]	*M. alba* [136]; *M. mongolica* [20]
( +)-Morusalone A (**154**)	+ 136.1 [137]	*M. alba* [137]
( +)-Morusalone B (**155**)	+ 130.7 [137]	*M. alba* [137]
( −)-Morusalone A (**156**)	− 129.7 [137]	*M. alba* [137]
( −)-Morusalone B (**157**)	− 132.9 [137]	*M. alba* [137]
Morusalone C (**158**)	+ 113.3 [137]	*M. alba* [137]
Morusalone D (**159**)	+ 109.4 [137]	*M. alba* [137]
Morusalisin A (**160**)	+ 93.7 [51]	*M. mongolica* [51]
Mongolicin B (**161**)	+ 8 [25]	*M. mongolica* [25]
Mulberrofuran H (**162**)	+ 25 [138]	*M. alba* [46, 128]; *M. bombycis* [71]; *M. lhou* [138]; *M. mongolica* [25]
Mulberrofuran R (**163**)		*M. lhou* [139]
Sanggenon S (**164**)	+ 58.8 [123]	*M. alba* [123]
Sanggenon B (**165**)	+ 62 [140]	*M. alba* [81, 120, 140]; *M. mongolica* [122]
Sanggenon R (**166**)	+ 243 [123]	*M. alba* [123]

^a#^Indicating that its methyl ether derivative has been totally synthesized; ^##^Indicating that it has been totally synthesized. ^b^*A.*, *Artocarpus*; *B.*, *Brosimopsis*; B.*, *Brosimum*; *C.*, *Chlorophora*; *D.*, *Dorstenia*; *M.*, *Moru*s; *S.*, *Sorocea*. ^c^n.m., not measured

As shown in the biosynthetic pathway of classic MDAAs (Scheme 1), a new methylcyclohexene ring with three chiral carbons (C-3", C-4", and C-5") was produced in the Diels–Alder cycloaddition of the chalcone dienophile and the dehydroprenylphenol diene, in which the relative configuration of H-4" and H-5" would always maintain the original *trans* configuration while the stereochemistry of H-3" and H-4" would have *cis* and *trans* configurations. Therefore, this kind of Diels–Alder product has *trans*–*trans* and *cis*–*trans* types, both of which are found in natural products. From the structural characteristics of *endo*- and *exo*-adducts in the Diels–Alder reaction, the *trans*–*trans* type adducts have been found to be *exo*-addition products, while the *cis*–*trans* type adducts are attributed to *endo*-addition. According to the current data statistics, the ratio of the reported natural MDAAs with *trans*–*trans* and *cis*–*trans* types is about 1:1.5. It is noteworthy that natural MDAAs seem to be formed in the manner of in vitro [4 + 2] cycloaddition reactions.

The *trans*–*trans* and *cis*–*trans* types of classic MDAAs could be easily determined by analysis of the coupling constant of H-4" with H-3" and H-5" (generally, large *J*_3"/4"_ and *J*_4"/5"_ for *trans*–*trans*; small *J*_3"/4"_ and large *J*_4"/5"_ for *cis*–*trans*:) in the ^1^H NMR spectrum with resolvable signals. Sometimes this kind of compound usually exists as an equilibrium mixture of conformational isomers in solution, which requires NMR variable temperature experiments to obtain the resolvable signals of the methylcyclohexene ring [17, 22, 74, 85, 94, 110, 118, 124]. Based on X-ray crystallographic analysis and circular dichroism (CD) spectroscopic evidence, in 1988 the Nomura group proposed the empirical rules for determining the absolute configuration of the chiral centers on the methylcyclohexene ring in classic MDAAs [12]: a) the stereochemistry of C-3", C-4", and C-5" in the *trans*–*trans* type MDAAs was 3"*R*,4"*R*,5"*S*, while in the *cis*–*trans* type MDAAs was 3"*S*,4"*R*,5"*S*. b) The *trans*–*trans* type MDAAs exhibited negative optical rotations, and their Cotton effect at the maximum UV absorption tended to be negative, while the *cis*–*trans* type MDAAs showed the opposite optical rotations and Cotton effect. More reports of natural and synthetic MDAAs with clear absolute structures established by single crystals analysis, chemical methods, or ECD calculation methods have confirmed the practicability of Nomura’s empirical rules. In this review, all MDAAs with clear relative configurations have determined their absolute configurations according to the empirical rules.

### Dehydroprenyl-2-arylbenzofuran type MDAAs (Type A)

**Fig. 2:**
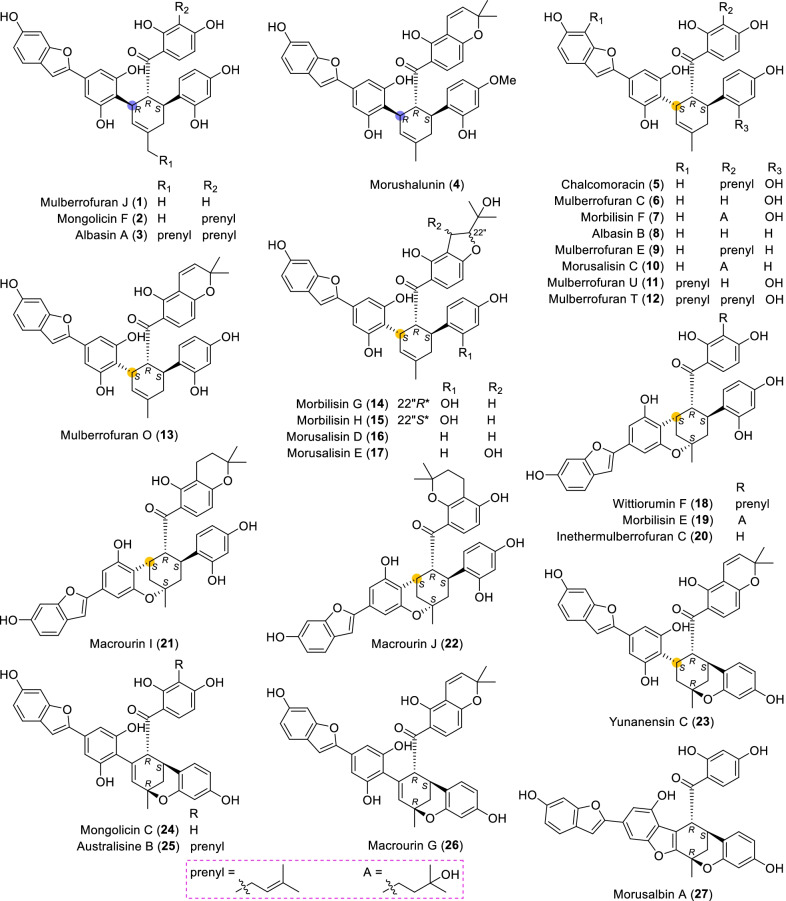
Structures of dehydroprenyl-2-arylbenzofuran type MDAAs (Type A)

**Fig. 3:**
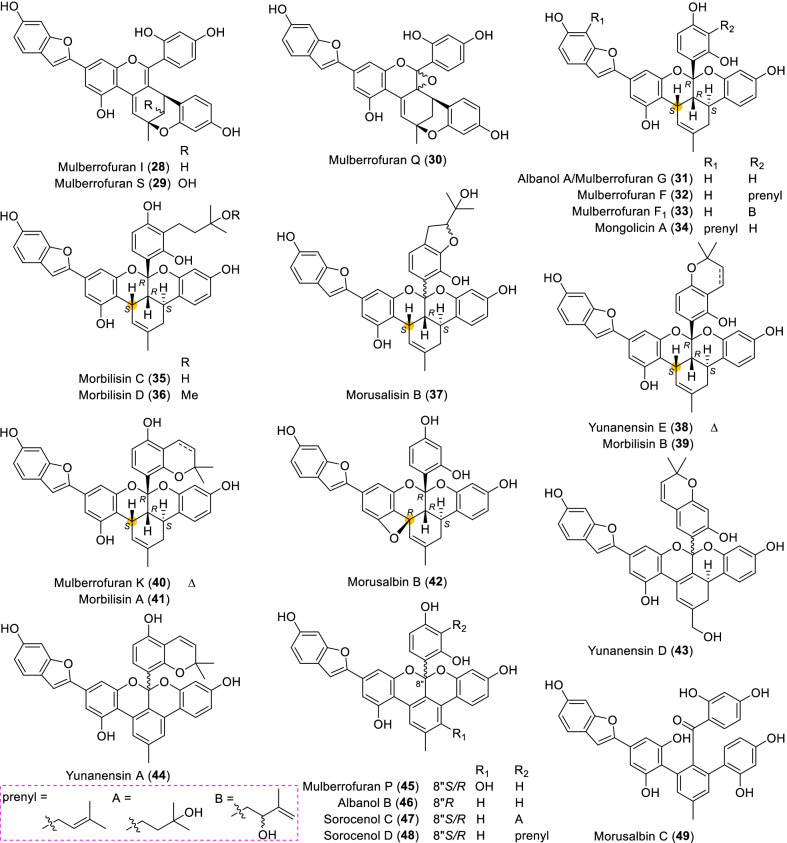
Structures of dehydroprenyl-2-arylbenzofuran type MDAAs (Type A, *continued*)

**Fig. 4:**
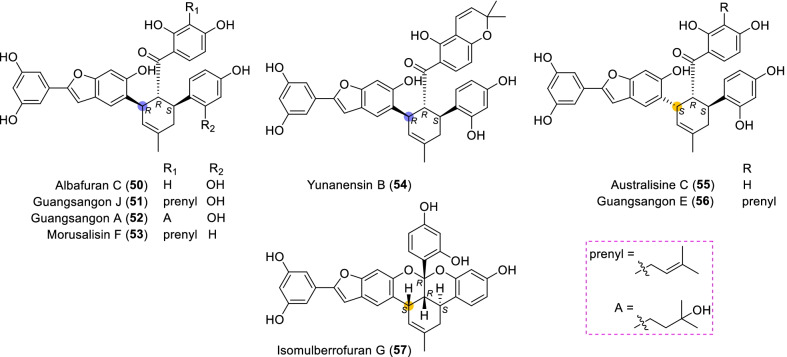
Structures of dehydroprenyl-2-arylbenzofuran type MDAAs (Type A, *continued*)

Until now, 57 type A of MDAAs (**1** − **57**) have been discovered, which compose the biggest family in MDAAs. This type of MDAAs was found mostly in the plants of *Morus*; its diene moiety was the dehydroprenyl group of 2-arylbenzofuran, which is predominantly located on its B ring (C-4′), as illustrated by the structures of compounds **1**–**49** (Figs. 2 and 3). In addition, there were only eight type A of MDAAs (**50**–**57**) with the diene moiety on A ring (C-5) of 2-arylbenzofuran (Fig. 4).

#### Type A with the diene moiety on ring B (Type A-I)

Mulberrofuran J (**1**) [11], mongolicin F (**2**) [25], albasin A (**3**) [14], and morushalunin (**4**) [26] were the only four type A-I MDAAs with *trans–trans* configuration found so far. Compound **1** was first isolated from the root barks of the cultivated mulberry tree *M. lhou* in 1985 by Hirakura et al*.* [11], and its absolute configuration was determined by the Nomura group in 1988 [12]. Albasin A (**3**) possessing a prenyl moiety at C-7″ was discovered from the root bark of *M. alba* [14]. MDAAs with a prenyl group or a modified prenyl group at C-7″ of the methylcyclohexene ring were rare, and only six examples have been reported. The others were macrourin E (**60**) [19] in type B, multicaulisin (**99**) [114] in type D, sanggenon G (**124**) [121], sanggenon T (**125**) [123], and sanggenol M (**126**) [122] in type E. Morushalunin (**4**) [26] isolated in *M. alba* was one of the only three methylated natural MDAAs, and the others were morbilisin D (**36**) [39] of the same type A from *M. notabilis* and kuwanon J 2,4,10"-trimethyl ether (**82**) [100] of type C from *M. alba*.

Compounds **5**–**23** represent a remarkable series of *cis–trans* type A-I MDAAs initially detected in plants of *Morus* [4, 14, 15, 22, 23, 39, 42, 47, 48, 51–54]. Chalcomoracin (**5**), which is the first example of MDAAs, was isolated from the diseased mulberry leaves (*M. alba*) by Takasugi et al*.* in 1980 [4]. Mulberrofuran C (**6**) and chalcomoracin (**5**) were the C-3" epimers of mulberrofuran J (**1**) and mongolicin F (**2**), respectively, all of which were detected in *M. macroura* [18, 19]. Both mulberrofuran U (**11**) [52] and mulberrofuran T (**12**) [53] have a prenyl unit at C-7, which were first obtained from *M. insignis* and *M. alba*, respectively. Morbilisins G and H (**14** and **15**), possessing isopropyldihydrobenzofuran rings, were a pair of the C-22" epimers firstly purified from the leaves of *M. notabilis* [39]. Due to their undefined absolute configurations of C-22", **14** and **15** were randomly assigned the R*- and S*-configurations, respectively, for the distinguishment of them. Morusalisin D (**16**) and its C-21" hydroxylated derive, morusalisin E (**17**), were recently obtained from cell cultures of *M. alba*, and the absolute configuraton of C-21" of **17** has not been confirmed [51]. The structures of compounds **18** − **22** [15, 22, 39, 48] were characteristic of a 6-membered oxygen ring, which might be formed by the intramolecular nucleophilic addition of the steric vicinal hydroxyl (3′- or 5′-OH) with the olefin (C-1") of the original methylcyclohexene ring. The successful transformation from chalcomoracin (**5**) to wittiorumin F (**18**) under 5% CF_3_COOH undoubtedly allowed the determination of the absolute configuration of **18** [22], and it was regarded as a model compound for ECD and specific rotations analysis to determine the absolute configuration of morbilisin E (**19**) [39], inethermulberrofuran C (**20**) [15], macrourin I (**21**) [48], and macrourin J (**22**) [48]. In addition, the ECD calculation of **20** using the TDDFT method further confirmed this way to determine the absolute configuration of these class compounds [15]. Yunanensin C (**23**) was another typical representative, which possessed a 6-membered oxygen ring with the same orientation of CH_3_-7" as H-5" formed by the intramolecular nucleophilic addition of 16″-OH with C-1" [23].

Compounds **24** − **30** [16, 19, 25, 46, 56–58], a series of type A-I MDAAs with a 6-membered oxygen ring similar to **23**, which primarily differed in their methylcyclohexene ring and C-8″ carbon as well as the prenyl substituent. The appearance of the double bond (Δ^2″^) on the methylcyclohexene ring made the C-3" chirality of these compounds disappear, while the absolute configuration of other chiral carbons remained unchanged. Morusalbin A (**27**) [46] featured with an additional furan ring formed by cyclization between C-2" and C-5′ through an oxygen atom, which could structurally derive from mongolicin C (**24**) [25]. Mulberrofuran I (**28**) [56] and mulberrofurans S and Q (**29** and **30**) [57, 58] were first detected in *M. bombycis* and *M. alba*, respectively, which could also be regarded as the 4*H*-pyran derivatives of **24**. Similarly, with **24** as the precursor, its 3′ or 5′-OH condensed with C-8" ketone to form a hemiketal intermediate, which was dehydrated to yield **28** and further oxidized to 6"-hydroxylated product **29** or the epoxy derivative **30**. The orientations of 5-OH in **29** and the epoxy ring in **30** were not yet determined.

Compounds **31** − **48**, a kind of ketalized type A-I MDAAs, were almost only discovered from the species of *Morus* [23, 25, 29, 39, 40, 46, 50, 51, 54, 64, 78–80]. All ketalized MDAAs were reported as *cis–trans* configuration based on the coupling constants of H-4" with H-3" and H-5" (small *J*_3"/4"_ and large *J*_4"/5"_). The relative configuration of the new chiral carbon C-8" of this class of ketalized MDAAs could be determined by the NOE correlations of 8"-Ar–H with methylcyclohexene ring protons, and then its absolute configuration could be assigned bases on the empirical rules. Albanol A (mulberrofuran G, **31**) was first isolated from *M. alba* bark by Rama Rao et al. in 1983, and its absolute configuration was confirmed by X-ray analysis of its pentamethyl ether [63]. In 1984, mulberrofuran F (**32**) and mulberrofuran G (**31**) were found from the root barks of *M. lhou* by Fukai et al. and mulberrofuran G was identified as albanol A by comparison of their NMR and physical data [64]. While the absolute configuration of **32** was determined by the Nomura group in 1988 [12]. In addition, compound **32** was also obtained in the above-mentioned conversion of **7** to **18** [22]. Mongolicin A (**34**), the only ketalized MDAAs possessing a prenyl unit in the moiety of diene, which was isolated from the stem and root bark of *M. mongolica* [25]. Morusalisin B (**37**) without analysis of the relative configuration of C-8″ was discovered from cell cultures of *M. alba*, and the stereochemistry of C-22″ was not defined [51]. Morusalbin B (**42**) being characteristic of a 4-membered oxygen ring were purified from the root bark of *M. alba* [46]. Yunanensin D (**43**), possessing the hydroxylated methyl and conjugated double bond on cyclohexene ring, was isolated from *M. yunanensis* [78]. If the cyclohexene ring with conjugated double bond continues to dehydrogenate, an aromatic ring will be obtained, as exemplified by compounds **44** − **49** [40, 46, 50, 79, 80]. Sorocenols C and D (**47** and **48**) were obtained from the root bark of *Sorocea bonplandii*, which were racemic mixtures due to their zero optical rations [40]. Compared with albanol B (**46**) [50], the specific optical rotation values of yunanensin A (**44**) [79] and mulberrofuran P (**45**) [80] are relatively small (Table 1), and they may also be racemic mixtures in which the ratio of (8"*R*)-**44** or **45** was greater than its (8"*S*)-enantiomer. Morusalbin C (**49**), the first non-ketalized MDAAs with an aromatic ring instead of cyclohexene ring, was recently identified from *M. alba* [46].

#### Type A with the diene moiety on ring A (Type A-II)

Only eight MDAAs in type A-II have been discovered so far, and they also contained *trans–trans* (**50** − **54**) [17, 51, 78, 84, 85] and *cis–trans* (**55** − **57**) [16, 66, 85] configurations. Albafuran C (**50**), the first type A-II MDAAs to be reported, was isolated from *M. alba* by Mitsuo et al. in 1982 [84], and its C-3" epimer australisine C (**55**) was obtained from *M. australis* by Zhang et al. in 2007 [16]. Guangsangon J (**51**) [17] and guangsangon E (**56**) [85], another pair of the C-3" epimers in type A-II MDAAs, were isolated from *M. macroura*. The only one ketalized type A-II MDAAs isomulberrofuran G (**57**) [66] was discovered from *M. alba*, whose possible precursor was compound **55**.

### Dehydroprenylstilbene type MDAAs (Type B)

**Fig. 5:**
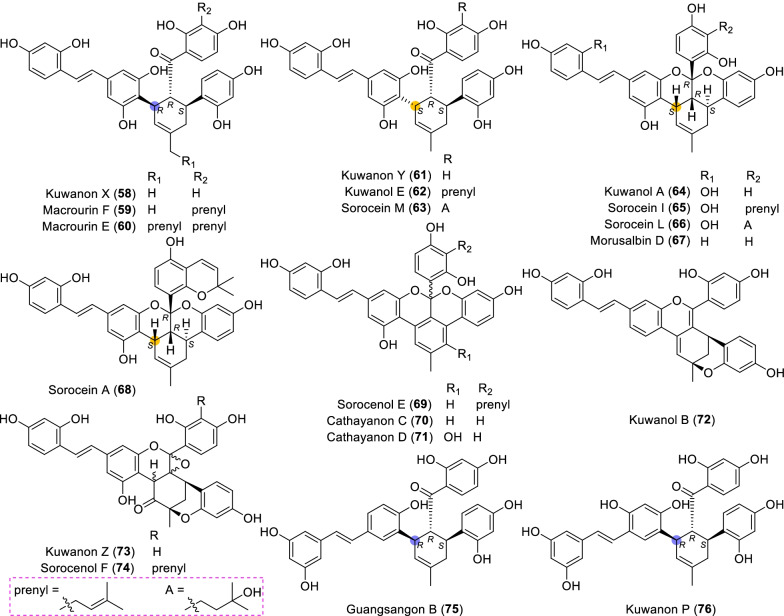
Structures of dehydroprenylstilbene type MDAAs (Type B)

A total of 19 MDAAs (Fig. 5, **58** − **76**) with dehydroprenylstilbene as the diene have been reported so far. Structurally, most of them (**58** − **74**) have the original dehydroprenyl group at the *para*-position of ring A, and only two compounds (**75** and **76**) at the *meta*-position of ring A.

#### Type B with the diene moiety at the *para*-position of ring A (Type B-I)

Three compounds with a *trans*–*trans* configuration were found in the plant of *M. macroura*, and they were kuwanon X (**58**) [18], macrourin F (**59**) [19], and macrourin E (**60**) [19]. Compounds **61** − **68** were *cis–trans* type B-I MDAAs, which were mainly isolated from *Morus* and *Sorocea* [40, 46, 53, 55, 86, 88–90]. Kuwanon Y (**61**) [86] and macrourin E (**62**) [53] were the C-3" epimers of **58** and **59**, respectively. Among the ketalized type B-I MDAAs (**64** − **71**), sorocenol E (**69**) [40], cathayanon C (**70**) [91], and cathayanon D (**71**) [91] were characterized by an aromatic ring rather than a cyclohexene ring. In addition, like sorocenols C and D (**47** and **48**), sorocenol E (**69**) with zero optical ration was also a racemic mixture [40]. In view of small specific optical rotation values of **70** and **71**, they might be racemic mixtures with different ratio of (8"*R*) and (8"*S*)-enantiomers. Kuwanol B (**72**) [88] featured a 6-membered oxygen ring and a 4*H*-pyran ring, similar to compound **29** in type A-I. Kuwanon Z (**73**) could be considered as a highly oxidized derivative of **72**, which was isolated from *M. alba* [86]. Sorocenol F (**74**) [40] obtained from *S. bonplandii* was a prenylated derivative of **73**.

#### Type B with the diene moiety at the *meta*-position of ring A (Type B-II)

The only two MDAAs of type B-II, guangsangon B (**75**) [85] and kuwanon P (**76**) [92], were first obtained from *M. macroura* and *M. lhou*. They both possess the *trans–trans* configuration.

### Dehydroprenylchalcone type MDAAs (Type C)

**Fig. 6:**
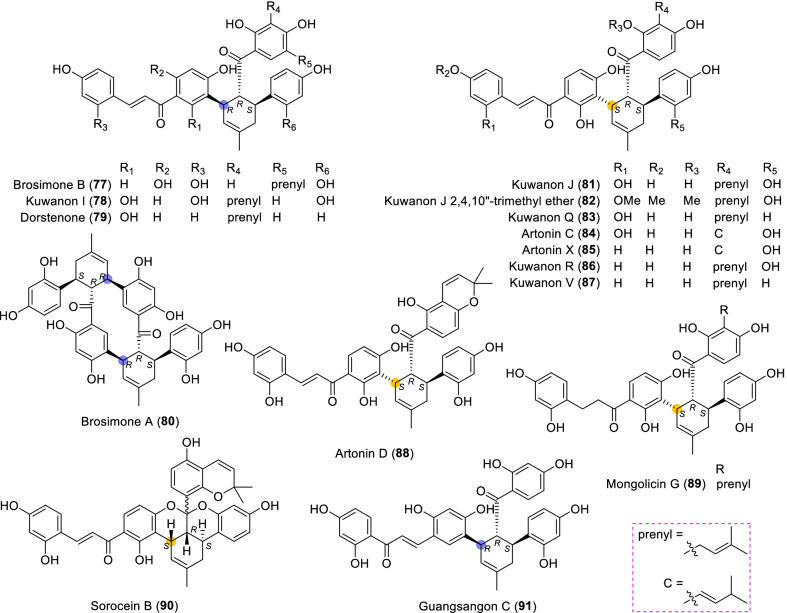
Structures of dehydroprenylchalcone type MDAAs (Type C)

There are 15 MDAAs (Fig. 6, **77** − **91**) formed by cycloaddition of chalcone dienophile with dehydroprenylchalcone diene, and the dienes and dienophiles of most type C MDAAs (**77** − **90**) are structurally derived from the same prenylatedchalcone. This type MDAAs is distributed in 11 species including *A. heterophyllus*, *B. oblongifolia*, *D. barteri*, *M. alba* (*M. alba* var. *shalun****)***, *M. bombycis*, *M. macroura*, *M. mongolica*, *M. notabilis*, *M. nigra*, *S. bonplandii*, and *S. ilicifolia*. According to the position of the dehydroprenyl group on chalcone skeleton, type C MDAAs could be divided into the following two subgroups.

#### Type C with the diene moiety on ring B (Type C-I)

Fourteen type C-I MDAAs have been reported so far, whose dehydroprenyl groups are located on ring B of a chalcone skeleton. Four of them are provided with *trans–trans* configuration (**77** − **80**) and ten with *cis–trans* configuration (**81** − **90**).

Kuwanon I (**78**) [94] and kuwanon J (**81**) [28] together with dorstenone (**79**) [95] and kuwanon V (**87**) [47] are two pairs of 3"-epimers. Brosimone A (**80**) [96], the only one MDAAs being characteristic of two methylcyclohexene rings, could be considered to be derived from brosimone B (**77**) [93] via Deils-Alder cycloaddition between the prenyl moiety at C-13" and the chalcone. Brosimones A and B (**80** and **77**) in this type as well as brosimone D (**98**) in type D are the only three MDAAs isolated from the root of *Brosimopsis oblongifolia* (Table 2) [93, 96]. Kuwanon J 2,4,10"-trimethyl ether (**82**) [100] from *M. alba* is the trimethyl ether of **81**. Comparion with kuwanons J and R (**81** and **86**) [28, 47], artonins C and X (**84** and **85**) [101, 102] from *A. heterophyllus* have a 3-methylbut-1-en-1-yl unit at C-11" instead of a prenyl group. Among the reported type C MDAAs, mongolicin G (**89**) is the only one with dehydroprenyldihydrochalcone as diene, which was identified from *M. mongolica* [104]. The only ketalized type C MDAAs, sorocein B (**90**), was obtained from the roots of *S. bonplandii* by Messana et al. in 1991 [90].

**Table 2:** Types and quantities of MDAAs distributed in different plant sources

No	Species^a^	Total of MDAAs	Type of MDAAs, quantity of each type (compound number)^b^
1	*A. heterophyllus*	6	Type C, 5 (**81**, **84** − **86** and **88**); Type D, 1 (**101**)
2	*A. integer*	3	Type C, 2 (**81** and **86**); Type D, 1 (**102**)
3	*B. oblongifolia*	3	Type C, 2 (**77** and **80**); Type D, 1 (**98**)
4	B.* rubescens	7	Type G, 7 (**138** − **144**)
5	*C. regia*	3	Type A, 1 (**5**); Type B, 1 (**62**); Type F, 1 (**130**)
6	*D. barteri*	1	Type C, 1 (**79**)
7	*M. alba* (including *M. alba* var. *shalun*)	71	Type A, 31 (**1**, **3 − 6**, **8**, **9**, **10**, **12**, **13**, **16**, **17**, **20**, **24**, **26**, **27**, **29** − **33**, **37**, **40**, **44** − **46**, **49**, **50**, **53**, **56**, and **57**); Type B, 5 (**61**, **62**, **64**, **67**, and **73**); Type C, 5 (**78**, **81**, **82**, **86**, and **87**); Type D, 7 (**92**, **93**, **95**, **96**, **99**, **101**, and **109**); Type E, 4 (**118**, **119**, **124**, and **125**); Type F, 5 (**128** − **132**); Type H, 2 (**146** and **148**,); Type I, 12 (**151**, **153** − **159**, **162**, and **164** − **166**)
8	*M. australis*	15	Type A, 9 (**1**, **5**, **9**, **24**, **25**, **30** − **32**, and **55**); Type D, 3 (**95**, **110**, and **113**); Type F, 2 (**118** and **119**); Type E, 1 (**125**)
9	*M. bombycis*	16	Type A, 5 (**5**, **6**, **28**, **31**, and **40**); Type B, 2 (**64** and **72**); Type C, 4 (**81**, **83**, **86**, and **87**); Type D, 2 (**92** and **93**); Type F, 2 (**130** and **131**); Type I, 1 (**162**)
10	*M. cathayana*	8	Type B, 2 (**70** and **71**); Type F, 6 (**128**, **130**, **131**, **133**, **136**, and **137**)
11	*M. insignis*	1	Type A, 1 (**11**)
12	*M. lhou*	15	Type A, 4 (**1**, **31**, **32**, and **50**); Type B, 2 (**58** and **76**); Type D, 5 (**92**, **93**, **97**, **109**, and **110**); Type E, 1 (**119**); Type I, 3 (**151**, **162**, and **163**)
13	*M. macroura*	38	Type A, 17 (**1**, **2**, **5**, **6**, **9**, **13**, **18**, **21**, **22**, **26**, **31**, **32**, **40**, **50** − **52**, and **56**); Type B, 9 (**58** − **62**, **65**, **68**, **75**, and **76**); Type C, 2 (**81** and **91**); Type D, 3 (**105**, **106**, and **111**); Type E, 6 (**114**, **115**, and **120** − **123**); Type H, 1 (**145**)
14	*M. mesozygia*	5	Type A, 1 (**5**) Type D, 4 (**99**, **101**, **103**, and **104**)
15	*M. mongolica* (syn. *M. yunanensis*)	45	Type A, 19 (**1**, **2**, **5**, **9**, **12**, **13**, **20**, **24**, **30** − **32**, **34**, **38**, **40**, **43**, **44**, **46**, **51**, and **54**); Type B, 5 (**58**, **64**, **68**, **75**, and **76**); Type C, 2 (**81** and **89**); Type D, 3 (**92** − **94**); Type E, 3 (**118**, **119**, and **124** − **126**); Type F, 3 (**128**, **130**, and **131**); Type H, 2 (**145** and **150**); Type I, 6 (**152**, **153**, **160** − **162**, and **165**)
16	*M. multicaulis*	1	Type D, 1 (**99**)
17	*M. nigra*	14	Type A, 5 (**1, 5, 31**, **46**, and **50**); Type C, 1 (**81**); Type D, 2 (**92** and **93**); Type E, 1 (**118**); Type F, 4 (**129 − 132**); Type H, 1 (**147**)
18	*M. notabilis*	10	Type A, 9 (**6**, **7**, **14**, **15**, **19**, **35**, **36**, **39**, and **41**); Type C, 1 (**81**)
19	*M. wittiorum*	15	Type A, 8 (**1**, **2**, **5**, **9**, **13**, **18**, **31**, and **50**); Type B, 1 (**68**); Type D, 3 (**107**, **108**, and **112**); Type E, 3 (**116**, **117**, and **127**)
20	*S. bonplandii*	15	Type A, 4 (**5**, **40**, **47**, and **48**); Type B, 4 (**62**, **68**, **69**, and**74**); Type C, 3 (**81**, **88**, and **90**); Type F, 2 (**132** and **134**); Type H, 2 (**147** and **149**)
21	*S. ilicifolia*	9	Type A, 2 (**5** and **13**); Type B, 5 (**62**, **63**, **65**, **66**, and **68**); Type C, 2 (**81** and **90**)

^a^*A.*, *Artocarpus*; *B.*, *Brosimopsis*; B.*, *Brosimum*; *C.*, *Chlorophora*; *D.*, *Dorstenia*; *M.*, *Morus*; *S.*, *Sorocea*. ^b^Type A, Dehydroprenyl-2-arylbenzofuran type MDAAs; Type B, Dehydroprenylstilbene type MDAAs; Type C, Dehydroprenylchalcone type MDAAs; Type D, Dehydroprenylflavone type MDAAs; Type E, Dehydroprenyldihydroflavone type MDAAs; Type F, Dehydroprenylsanggenonflavone type MDAAs; Type G, Dehydroprenylcoumarin MDAAs; Type H, Simple or other dehydroprenylphenol type MDAAs; Type I, Non-classic MDAAs

#### Type C with the diene moiety on ring A (Type C-II)

Only one member, namely guangsangon C (**91**), in this subgroup has been identified so far. As shown in Fig. 6, the position of its dehydroprenyl group is located at C-3 on ring A of the chalcone skeleton, and **91** has the *trans–trans* configuration. Guangsangon C (**91**) was isolated from the stem bark of *M. macroura* [85].

### Dehydroprenylflavone type MDAAs (Type D)

**Fig. 7:**
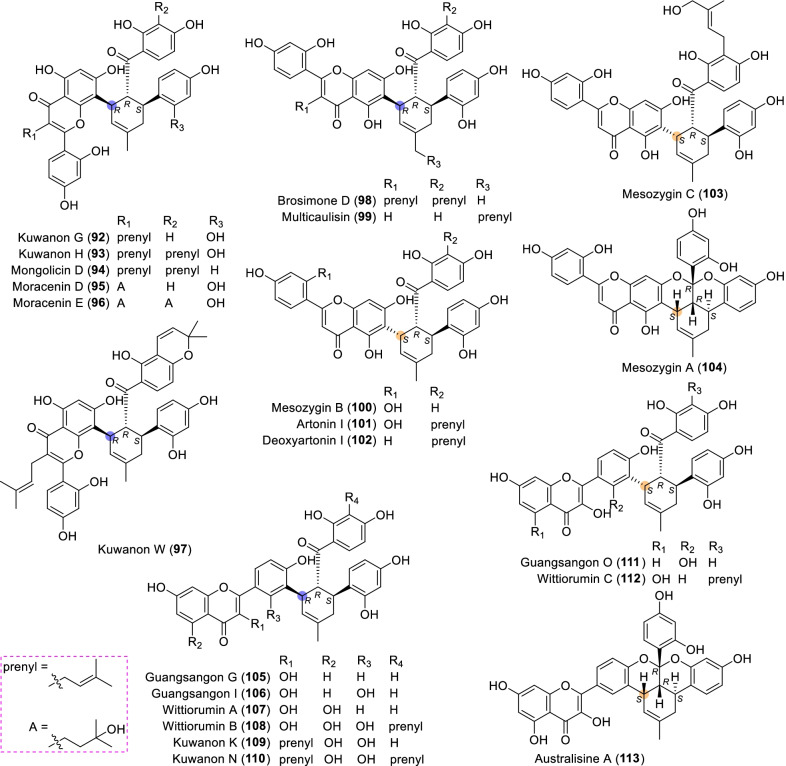
Structures of dehydroprenylflavone type MDAAs (Type D)

Twenty-two MDAAs (Fig. 7, **92** − **113**) with dehydroprenylflavone as the diene have been reported so far. On the basis of the position of dehydroprenyl unit, type D MDAAs are divided into two subgroups: type D-I (the diene moiety on ring A) and type D-II (the diene moiety on ring B), as illustrated by the structures of **92**–**104** and **105**–**113**, respectively.

#### Type D with the diene moiety on ring A (Type D-I)

Among type D-I MDAAs **92**–**104**, the diene moiety of six compounds (**92**–**97**) are located at C-8 of A ring and others (**98**–**104**) at C-6 of A ring. All six type D-I MDAAs with the diene moiety at C-8 of A ring, namely kuwanon G (**92**), kuwanon H (**93**), mongolicin D (**94**), moracenin D (**95**), moracenin E (**96**), kuwanon W (**97**), are provided with *trans–trans* configuration. The other seven type D-I MDAAs with the diene moiety at C-6 of A ring have *trans–trans* and *cis–trans* configurations, as exemplified by **98**–**99** and **100**–**104**, respectively.

Kuwanon G (**92**) [1] and kuwanon H (**93**) [2], also known as albanin F and albanin G [3] as well as moracenin B [109] and moracenin A [112], respectively, are the first reported MDAAs by three research groups in 1980 from the same plant (*M. alba*). Their structures were further confirmed by partial synthesis in 1981 [5]. Moracenin D (**95**) was isolated from *M. alba*, and its structure was elucidated by oxidation of **92** [105]. Compounds **92**–**98** all possess a prenyl group or structure derived thereof at C-3. Multicaulisin (**99**) possessing an additional prenyl moiety at C-7″ was isolated from *M. multicaulis* by Ferrari et al. in 2000 [114]. Mesozygins A (**104**), B (**100**), and C (**103**) as well as artonin I (**101**) were all discovered in the leaves of *M. mesozygia* by Fozing et al. in 2012 [35]. Mesozygin C (**103**) is a prenylated derivative of mesozygin B (**100**), while mesozygin A (**104**) is a ketal form of compound **100**.

#### Type D with the diene moiety on ring B (Type D-II)

There are only nine members (**105**–**113**) in this subgroup, and their diene moieties are all placed at C-3′ of B ring. Among type D-II MDAAs, compounds **105**–**110** are *trans–trans* configurations, while **111**–**113** are *cis–trans* configurations. Guangsangons G, I, and O (**105**, **106**, and **111**) from *M. macroura* [17, 119], wittiorumins A, B, and C (**107**, **108**, and **112**) from *M. wittiorum* [22], and australisine A (**113**) from *M. australis* [16] feature dehydroprenylflavonol dienes. Kuwanon K (**109**) and kuwanon N (**110**), possessing a prenyl unit at C-3 of dehydroprenylflavone diene, were discovered from *M. lhou* [110]. Australisine A (**113**) is the only ketal compound in type D-II MDAAs, which has been reported only once from moraceous plants [16].

### Dehydroprenyldihydroflavone type MDAAs (Type E)

**Fig. 8:**
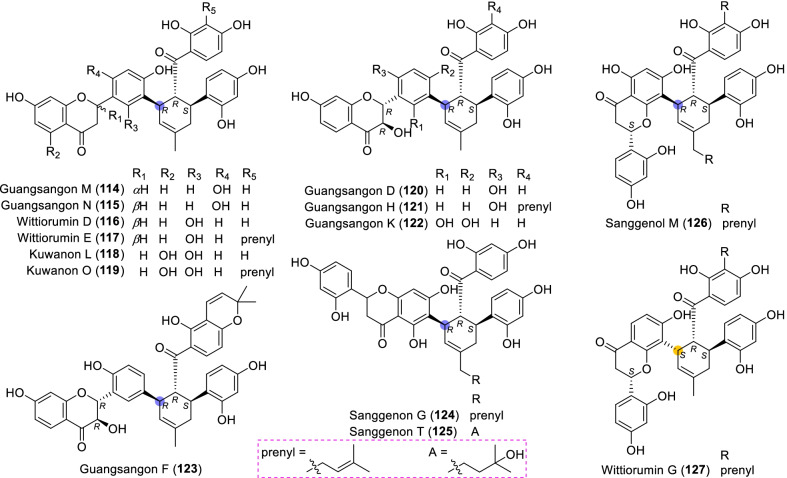
Structures of dehydroprenyldihydroflavone type MDAAs (Type E)

Fourteen type E MDAAs have been reported to date, as exemplified by **114** − **127** (Fig. 8), all sharing the dehydroprenyldihydroflavone diene. The location of the dehydroprenyl at dihydroflavone result in two subgroups: type E-I (the diene moiety on ring B) and type E-II (the diene moiety on ring A).

#### Type E with the diene moiety on ring B (Type E-I)

Among the reported type E-I MDAAs, all compounds having the diene moiety on ring B (all at C-3′) are *trans–trans* configuration. Guangsangons M and N (**114** and **115**) are a pair of the 2-epimers, which were isolated from the stem barks of *M. macroura* by Dai et al*.* in 2004 [74]. Wittiorumin D (**116**) and its prenylated derivative wittiorumin E (**117**) with the same 2*S* configuration were obtained from *M. wittiorum* by Tan et al. in 2009 [22]. Kuwanon L (**118**) [118] and its prenylated derivative kuwanon O (**119**) [110] wereisolated from *M. alba* in 1983 and *M. lhou* in 1984, respectively, in which the configurations of C-2 were not yet determined. Guangsangon D (**120**) [85], guangsangon H (**121**) [17], guangsangon K (**122**) [74], and guangsangon F (**123**) [74] all having a hydroyl group at C-2 are 2*R*,3*R* configurations. Their dienes also could be regarded as dehydroprenyldihydroflavonol.

#### Type E with the diene moiety on ring A (Type E-II)

There are only four compounds in type E-II MDAAs, two (**124** and **125**) of which feature the C-6 dehydroprenyl and two (**126** and **127**) feature the C-8 dehydroprenyl. Compounds **124** − **126** are *trans–trans* configurations, while **127** is *cis–trans* configuraion. Sanggenon G (**124**) [121] and sanggenon T (**125**) [123] have a prenyl group or structure derived thereof at C-7", in which the absolute configurations of C-2 were still not confirmed. Sanggenol M (**126**) is also characterized by the rare C-7" prenyl, which was isolated from the root barks of *M. mongolica* [122]. Wittiorumin G (**127**) obtained from *M. wittiorum* is the only type E MDAAs with *cis–trans* configuration [49].

### Dehydroprenylsanggenonflavone type MDAAs (Type F)

**Fig. 9:**
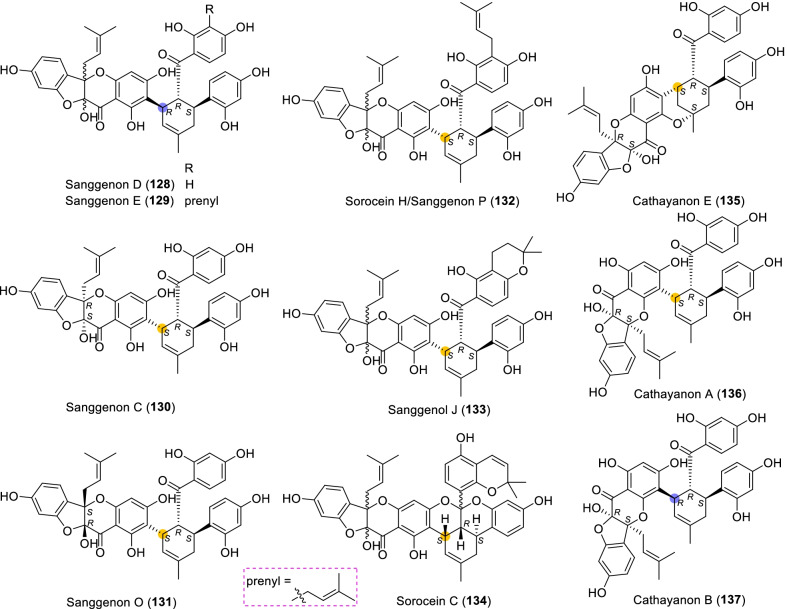
Structures of dehydroprenylsanggenonflavone type MDAAs (Type F)

Sanggenon-type flavanones are a kind of 3-hydroxy-2-prenylflavanones having an ether linkage between C-3 and C-2', which are characteristic constituents in *Morus* plants. Sanggenon-type flavanones are very rare in nature, so only several type F MDAAs (Fig. 9, **128** − **137**) derived from sanggenon-type flavanone with the dehydroprenyl group have been reported so far. According to the position of the dehydroprenyl group on sanggenon-type flavanone, type F MDAAs could be divided into the following two subgroups.

#### Type F with the diene moiety at C-6 of ring B (Type F-I)

Eight members (**128** − **135**) in type F-I MDAAs have so far been reported, which are distributed in five species of the family Moraceae including *M. alba*, *M. cathayana*, *M. mongolica*, *M. nigra*, and *S. bonplandii* [91, 99, 124–126, 129, 131]. Among this subgroup, two compounds (**128** and **129**) are provided with *trans–trans* configuration and six (**130** − **135**) with *cis–trans* configuration. Sanggenon D (**128**) [124], sanggenon E (**129**) [126], sanggenon C (**130**) [129], sanggenon O (**131**) [131], and sanggenon P (**132**) [126], isolated from *M. alba* by Nomura et al. in the 1980s, have been revised to be the present structures shown in Fig. 9 rather than the original structures derived from 2-hydroxy-3-prenylflavanones having an ether linkage between C-3 and C-2'. Except for compounds **130**, **131**, and **135**, the absolute configurations of C-2 and C-3 of **128**, **129**, and **132 − 134** have not been determined. Sorocein C (**134**) belonging to the ketalize MDAAs, was isolated from the root bark of *S. bonplandii* [99]. Cathayanon E (**135**) has a 6-membered oxygen ring formed by the intramolecular nucleophilic addition of 5-OH with the olefinic C-1", which was obtained from the stem bark of *M. cathayana* by Zhang et al. in 2009 [91].

#### Type F with the diene moiety at C-8 of ring B (Type F-II)

Cathayanons A and B (**136** and **137**) are the only two type F MDAAs with their dehydroprenyl group at C-8 of ring B, which were isolated from *M. cathayana* by Shen et al. in 2001 [130]. The absolute structure of the *cis–trans* type adduct cathayanon A (**136**) was confirmed by X-ray crystallographic analysis. The stereochemistry of C-2 and C-3 in the *trans*–*trans* type adduct cathayanon B (**137**) was determined to be the same 2*S*,3*R* as compound **136**.

### Dehydroprenylcoumarin type MDAAs (Type G)

**Fig. 10:**
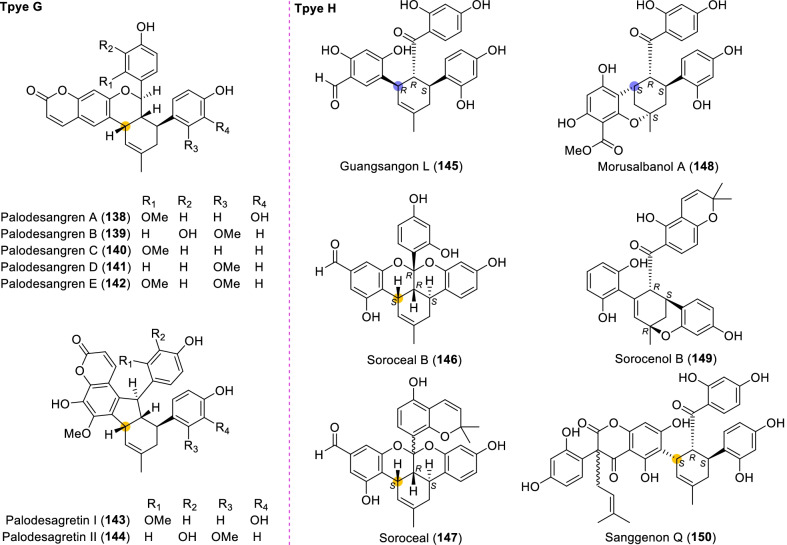
Structures of dehydroprenylcoumarin type MDAAs (Type G) and simple or other dehydroprenylphenol type MDAAs (Type H)

There are only seven members (Fig. 10, **138**–**144**) in this type, in which the dehydroprenyl group is located on its phenyl ring (C-6). All these compounds were isolated from the bark of *Brosimum rubescens* by Shirota et al*.* [132], and they share the same *cis–trans* configuration. The common characteristic of palodesangrens A − E (**138 − 142**) is the presence of a 6-membered oxygen ring formed by an ether linkage between C-7 and C-8", and the main difference is the number and position of methoxy groups on the chalcone unit. Palodesagretins I and II (**143** and **144**) [133] have an additional 5-membered ring formed by the carbon bond of C-5 and C-8", and they are different in the position of methoxy groups. The absolute configurations of **138**–**144** were not determined.

### Simple or other dehydroprenylphenol type MDAAs (Type H)

Compounds **145** − **149** (Fig. 10) are the simplest MDAAs found in moraceous plants, and their dienes are the simple phenolic compounds with the dehydroprenyl moiety. These simple phenolic compounds include 2,4-dihydroxybenzaldehyde, 3,5-dihydroxybenzaldehyde, methyl 2,4,6-trihydroxybenzoate, and resorcinol, as exemplified by **145**, **146** and **147**, **148**, and **149**, respectively. Guangsangon L (**145**) [74] from the stem bark of *M. macroura* is *trans–trans* configuration. The ketalized type H MDAAs, soroceal B (**146**) [13] from *M. alba* and soroceal (**147**) [90] from *S. bonplandii*, have *cis*–*trans* configuration. Morusalbanol A (**148**) [134] from *M. alba*, possessing the same 6-membered oxygen ring as **18** − **22**, has *cis*–*trans* configuration. Sorocenol B (**149**), having the same methylcyclohexene ring and 6-membered oxygen ring as **24** − **26**, was obtained from the root bark of *S. bonplandii* [87]. Sanggenon Q (**150**) [83] from *M. mongolica* is the only other dehydroprenylphenol type MDAAs, in which the diene is dehydroprenyl-2-oxo-3-prenylisoflavanone and the relative configuration of H-3″, H-4″, and H-5″ is *cis–trans*. The stereochemistry of its C-3 was not determined.

### Non-classic MDAAs (Type I)

**Fig. 11:**
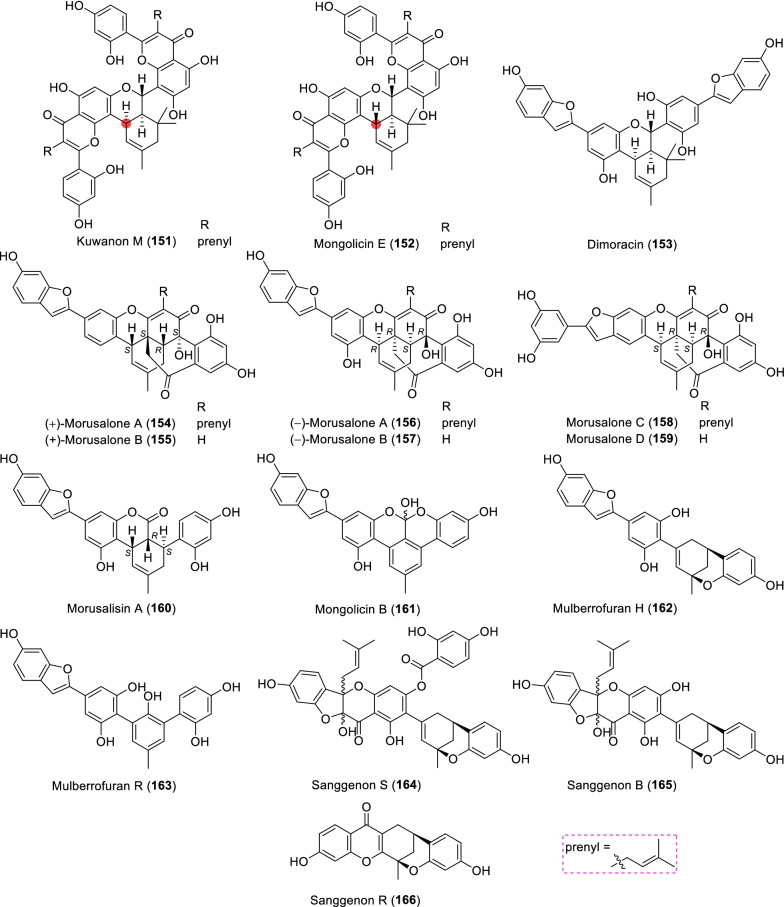
Structures of non-classic MDAAs (Type I)

There are a few non-classic MDAAs (Fig. 11, **151** − **166**) in moraceous plants, in which the dienophile moiety is the olefinic bond of other compounds rather than the *α*,*β*-unsaturated bond of a chalcone skeleton, or which might be derived from some classic MDAAs without the benzoyl at C-4" or the phenyl at C-8". The distribution of non-classic MDAAs in mulberry plants is limited, which were only found in the following three *Morus* plants, *M. alba*, *M. lhou*, and *M. mongolica*.

Kuwanon M (**151**) [135] from *M. lhou* and mongolicin E (**152**) [25] from *M. mongolica* are a pair of epimers, in which the dienophile and diene are delivered from the same prenylatedflavone, kuwanon C [33]. Specifically, after Diels–Alder reaction of the C-8 dehydroprenyl group in one molecule and the C-8 prenyl group in another molecule, a further intramolecular cyclodehydrogenation builds a 6-membered oxygen ring in the additions. Dimoracin (**153**) was isolated from *M. alba* [136], and its formation process is similar to that of **151** and **152**, except that its dienophile and diene are derived from the same prenylated-2-arylbenzofuran, moracin C [136].

Morusalones A − D (**154** − **159**) from *M. alba* cell cultures [137], featuring unprecedented 6/7/6/6/6/6 hexacyclic core skeletons with a unique bridged cycloheptenone ring, could be regarded to be formed by Diels–Alder cycloaddition of quinostilbene dienophiles and dehydroprenyl-2-arylbenzofuran dienes and subsequent intramolecular nucleophilic addition. The absolute configurations of **154** − **159** were determined by X-ray analysis and ECD calculation [137]. Morusalones A and B are the first examples of Diels − Alder adduct enantiomers from the genus *Morus*.

Seven type I MDAAs might be variations of some classic MDAAs, in which the phenyl moieties at C-8" or the benzoyl moieties at C-4" is missing, as exemplified by compounds **160** − **161** [25, 51] or **162** − **166** [123, 138–140], respectively. Among these compounds, sanggenon S (**164**) [123] from *M. alba* is a typical intermediate in the formation of non-classic MDAAs, which seems to be a derivative induced from sanggenon D (**128**). Sanggenon B (**165**) [140] was also isolated from *M. alba*, which can be regarded as derived from **164** by hydrolyzing its 2,4-dihydroxybenzoyl group.

### Overview on distribution of MDAAs in different plants

MDAAs were at least found in 21 species of the family Moraceae, most of which were isolated and identified from *Morus* plants (Tables 1 and 2). Dehydroprenyl-2-arylbenzofuran type (Type A) MDAAs with 57 compounds were widely distributed in 14 species of three genera (*Morus*, *Sorocea*, and *Chlorophora*), belonging to the richest class on MDAAs, followed by dehydroprenylflavone type (Type D) MDAAs with 22 structures distribution in 13 species of three genera (*Morus*, *Artocarpus*, and *Brosimopsis*) and dehydroprenylstilbene type (Type B) MDAAs with 19 compounds distribution in ten species of three genera (*Morus*, *Sorocea*, and *Chlorophora*) (Table 1). Dehydroprenyldihydroflavone type (Type E) MDAAs (14 compounds) and non-classic (Type I) MDAAs (16 compounds) were only identified in *Morus* plants, while dehydroprenylcoumarin type (Type G) MDAAs (seven compounds) was only found in one plant *Brosimum rubescens*. Although there were only 15 compounds in dehydroprenylchalcone type (Type C) MDAAs, they were distributed in five genera (*Morus*, *Artocarpus*, *Sorocea*, *Brosimopsis*, and *Dorstenia*), which was the type with the largest distribution of genera among all types. Most of the dehydroprenylsanggenonflavone type (Type F) MDAAs (ten compounds) were isolated from five *Morus* plants (*M. alba*, *M. bombycis*, *M. cathayana*, *M. mongolica*, and *M. nigra*), and simple or other dehydroprenylphenol type (Type H) MDAAs (five compounds) were distributed in five species including *M. alba*, *M. macroura*, *M. mongolica* (syn. *M. yunanensis*), *M. nigra*, and *S. bonplandii* (Table 1).

*M. alba*, *M. mongolica*, *M. macroura*, *M. bombycis*, *M. australis*, *M. lhou*, *M. wittiorum*, and *S. bonplandii* are rich in MDAAs (Table 2). Among them, the plant of *M. alba* has the largest number MDAAs, more than 70, covering eight types including Types A − F, H, and I. *M. mongolica* contains 45 MDAAs also involving in all eight types except for Type G. 38 and 16 MDAAs were isolated from *M. macroura* and *M. bombycis*, respectively. There are no more than 10 MDAAs in other species, such as *D. barteri*, *M. insignis*, and *M. multicaulis* (each contains one MDAAs), *A. integer*, *Brosimopsis oblongifolia*, and *C. regia* (each contains three MDAAs), *M. mesozygia* (contains five MDAAs), *A. heterophyllus* (contains six MDAAs), *Brosimum rubescens* (contains seven MDAAs), *M. cathayana* (contains eight MDAAs), and *S. ilicifolia* (contains nine MDAAs) (Table 2). In addition, according to the distribution of MDAAs, this kind of compound could be used as chemotaxonomy biomarker within moraceous plants.

## Biological activities

As the characteristic components of *Morus* plants, MDAAs possess a variety of different biological activities, including antineoplastic, anti-inflammation, antimicrobial, antioxidant, antiviral, anti-neurodegenerative diseases, anti-cardiovascular diseases, as well as PTP1B, *α*-glucosidase, and tyrosinase inhibitory activities. In this section, we will focus predominantly on the biological and pharmacological activities of natural MDAAs.

### Antineoplastic activity

The Yu and Chen groups have evaluated the cytotoxicity of 18 MDAAs, mulberrofuran J (**1**), chalcomoracin (**5**), mulberrofuran E (**9**), mulberrofuran O (**13**), mongolicin C (**24**), australisine B (**25**), mulberrofuran Q (**30**), mulberrofuran G (**31**), mulberrofuran F (**32**), mulberrofuran F_1_ (**33**), yunanensin A (**44**), albafuran C (**50**), australisine C (**55**), sorocein A (**68**), kuwanon J (**81**), kuwanon G (**92**), australisine A (**113**), and wittiorumin G (**127**), against five human cancer cell lines (A549, Bel-7402, BGC-823, HCT-8, and A2780). The results showed that nine compounds (**5**, **24**, **25**, **31** − **33**, **44**, **55**, and **113**) possessed potent cytotoxic properties in these tested cell lines (Table 3) [16, 29, 49, 79]. Mulberrofuran J (**1**), mulberrofuran C (**6**), mongolicin C (**24**), mulberrofuran G (**31**), artonin I (**101**), and soroceal B (**146**) were tested for their cytotoxicity against HL-60, Hela, HepG-2, A-549, and AGS cell lines. Compounds **24**, **31**, and **146** exhibited obvious cytotoxic activity against the tested five cell lines (Table 3) and **31** inhibited significantly selective cytotoxic activities towards HL-60 and AGS cells with IC_50_ of 3.4 and 3.5 μM [13]. Fitriani et al. found that morushalunin (**4**), chalcomoracin (**5**), mulberrofuran K (**40**), and guangsangon E (**56**), and kuwanon J (**81**) had significant cytotoxicity against murine leukemia P-388 cells with IC_50_ values 0.7, 1.7 (5.46 μM [34]), 0.6, and 2.5 (2.75 μg/mL [34]), and 5.9 μg/mL, respectively [26, 77]. Han et al. showed for the first time that chalcomoracin (**5**) treatment markedly promoted paraptosis along with extensive cytoplasmic vacuolation derived from the endoplasmic reticulum, rather than apoptosis, in PC-3 and MDA-MB-231cell lines [141]. Subsequently, Zhang et al. demonstrated that chalcomoracin (**5**) could inhibit cell proliferation and increase sensitivity to radiotherapy in human non-small cell lung cancer cells also via endoplasmic reticulum stress-mediated paraptosis mechanism [142]. Takashi et al. revealed that mulberrofuran G (**31**) could induce apoptotic cell death in HL-60 via both the cell death receptor pathway by stimulation of death receptor, and the mitochondrial pathway by Topo II inhibition through caspase-2 activation [59]. In addition, **31** had moderate cytotoxic activity against lung cancer cells NCI-H292 and A549 with IC_50_ value of 3.75 and 10.39 μM, respectively [68]. Mulberrofuran G (**31**), albafuran C (**50**), and kuwanon G (**92**) possessed antiproliferative activity against HePG2 and MCF-7 cell lines, and the IC_50_ values were equivalent to those of the positive control [76]. Phan et al. demonstrated for the first time that albanol B (**46**) exerted the anti-cancer effect by inducing apoptosis and cell cycle arrest at G2/M through mitochondrial ROS production in lung cancer cells [143]. Shu et al. reported that guangsangon E (**56**) could exert anti-lung and nasopharyngeal cancer cells through autophagy-mediated cell death [144]. The cytotoxicity of sanggenon G (**124**), sanggenon T (**125**), sanggenol M (**126**), sanggenon D (**128**), sanggenon C (**130**), and sanggenon B (**165**) from *M. mongolica* was evaluated. Of these MDAAs, **126** and **130** were the most potent agents against human oral tumor cell lines (HSC-2 and HSG) [122]. Park et al. have confirmed for the first time that sanggenon G (**124**) could suppress proliferation and induce apoptosis in lung cancer cells (A549) through caspase-3 activation and PL5-mediated inhibition of c-Myc, and its combination effect with adriamycin was more prominent [145]. Sanggenon D (**128**) was found to inhibit the growth of transplanted tumor and the proliferation of tumor cells in mice [146]. Chen et al. reported that sanggenon C (**130**) could induce apoptosis of colon cancer cells via inhibition of NO production, iNOS expression, and ROS activation of the mitochondrial pathway [147]. It was found that sanggenon O (**131**) induced apoptosis of A549 cells could be counterbalanced by protective autophagy, which indicated that **131** possesses great potential to be a promising candidate combined with autophagy inhibitor for the treatment of NSCLC [148].

**Table 3:** Antineoplastic activities of MDAAs against tumor cell lines or target proteins

Compd.	Cell or Target protein^a^	Activity (IC_50_ in μM or μg/mL)	Positive control (IC_50_ in μM or μg/mL)^b^
Mulberrofuran J (**1**)	A549	n.a. [16]	0.2 μg/mL (5-FU)
	A2780	n.a. [16]	0.7 μg/mL (5-FU)
	Bel-7402	n.a. [16]	0.5 μg/mL (5-FU)
	BGC-823	n.a. [16]	0.7 μg/mL (5-FU)
	HCT-8	n.a. [16]	0.5 μg/mL (5-FU)
Morushalunin (**4**)	P-388	0.7 μg/mL [26]	–
Chalcomoracin (**5**)	A549	5.5 μg/mL [16]	0.2 μg/mL (5-FU)
	A2780	5.7 μg/mL [16]	0.7 μg/mL (5-FU)
	Bel-7402	5.6 μg/mL [16]	0.5 μg/mL (5-FU)
	BGC-823	5.6 μg/mL [16]	0.7 μg/mL (5-FU)
	HCT-8	7.0 μg/mL [16]	0.5 μg/mL (5-FU)
	LNCaP	8 μM [141]	–
	MDA-MB-231	6 μM [141]	–
	PC-3	6 μM [141]	–
	P-388	5.46 μM [34]; 1.7 μg/mL [26]	–
Mulberrofuran E (**9**)	A549	5.8 μg/mL [16]	0.2 μg/mL (5-FU)
	A2780	5.7 μg/mL [16]	0.7 μg/mL (5-FU)
	Bel-7402	6.6 μg/mL [16]	0.5 μg/mL (5-FU)
	BGC-823	5.9 μg/mL [16]	0.7 μg/mL (5-FU)
	HCT-8	6.7 μg/mL [16]	0.5 μg/mL (5-FU)
Mongolicin C (**24**)	A549	6.6 μg/mL [16]	0.2 μg/mL (5-FU)
	A549	16.8 μM [13]	–
	A2780	6.0 μg/mL [16]	0.7 μg/mL (5-FU)
	AGS	17.5 μM [13]	–
	Bel-7402	7.2 μg/mL [16]	0.5 μg/mL (5-FU)
	BGC-823	6.1 μg/mL [16]	0.7 μg/mL (5-FU)
	HCT-8	6.7 μg/mL [16]	0.5 μg/mL (5-FU)
	Hela	17.1 μM [13]	–
	HepG2	17.4 μM [13]	–
	HL-60	17.1 μM [13]	–
Australisine B (**25**)	A549	5.8 μg/mL [16]	0.2 μg/mL (5-FU)
	A2780	5.7 μg/m L [16]	0.7 μg/mL (5-FU)
	Bel-7402	6.1 μg/mL [16]	0.5 μg/mL (5-FU)
	BGC-823	5.4 μg/mL [16]	0.7 μg/mL (5-FU)
	HCT-8	6.8 μg/mL [16]	0.5 μg/mL (5-FU)
Mulberrofuran Q (**30**)	A549	n.a. [16]	0.2 μg/mL (5-FU)
	A2780	n.a. [16]	0.7 μg/mL (5-FU)
	Bel-7402	n.a. [16]	0.5 μg/mL (5-FU)
	BGC-823	n.a. [16]	0.7 μg/mL (5-FU)
	CRL1579	83.9 μM [59]	21.1 μM (CPT)
	HCT-8	n.a. [16]	0.5 μg/mL (5-FU)
	HL-60	9.8 μM [59]	1.9 μM (CPT)
Mulberrofuran G (**31**)	A549	5.2 μg/mL [16]	0.2 μg/mL (5-FU)
	A549	15.3 μM [13]	12.4 μM (CPT)
	A549	10.39 μM [68]	12.35 μM (CPT)
	A2780	5.0 μg/mL [16]	0.7 μg/mL (5-FU)
	AGS	3.5 μM [13]	18.8 μM (CPT)
	Bel-7402	n.a. [16]	0.5 μg/mL (5-FU)
	BGC-823	5.7 μg/mL [16]	0.7 μg/mL (5-FU)
	CRL1579	37.6 μM [59]	21.1 μM (CPT)
	HCT-8	8.9 μg/mL [16]	0.5 μg/mL (5-FU)
	Hela	9.6 μM [13]	4.9 μM (CPT)
	HepG2	14.4 μM [13]	12.5 μM (CPT)
	HepG2	20.37 μM [76]	41.55 μM (CUR)
	HL-60	1.7 μM [59]	1.9 μM (CPT)
	HL-60	3.4 μM [13]	3.7 μM (CPT)
	MCF-7	50.44 μM [76]	34.17 μM (5-FU)
	NCI-H292	3.75 μM [68]	7.36 μM (CPT)
	HIF-1*	2.60 μM [71]	0.0572 μM (ALV)
	VEGF*	28.6 μM [71]	0.0795 μM (ALV)
	Hep3B	> 30 μM [71]	> 30 μM (ALV)
Mulberrofuran F (**32**)	A549	4.9 μg/mL [16]	0.2 μg/mL (5-FU)
	A2780	4.6 μg/mL [16]	0.7 μg/mL (5-FU)
	Bel-7402	4.8 μg/mL [16]	0.5 μg/mL (5-FU)
	BGC-823	5.7 μg/mL [16]	0.7 μg/mL (5-FU)
	HCT-8	4.7 μg/mL [16]	0.5 μg/mL (5-FU)
Mulberrofuran F1 (**33**)	A549	8.18 μM [29]	–
	A2780	8.35 μM [29]	–
	Bel-7402	8.29 μM [29]	–
	BGC-823	8.46 μM [29]	–
	HCT-8	> 10 μM [29]	–
Mulberrofuran K (**40**)	P-388	0.6 μg/mL [77]	–
Yunanensin A (**44**)	A549	0.922 μg/mL [79]	–
	A2780	2.384 μg/mL [79]	–
	Bel7402	5.387 μg/mL [79]	–
	BGC-823	0.863 μg/mL [79]	–
	HCT-8	5.378 μg/mL [79]	–
Albanol B (**46**)	A549	5.6 μM [143]	23.5 μM (ETO)
	BZR	8.9 μM [143]	15.8 μM (ETO)
	NCI-H1975	12.7 μM [143]	18.5 μM (ETO)
	NCI-H226	15.0 μM [143]	14.4 μM (ETO)
Albafuran C (**50**)	HepG2	28.94 μM [76]	41.55 μM (CUR)
	MCF-7	30.57 μM [76]	34.17 μM (5-FU)
Australisine C (**55**)	A549	n.a. [16]	0.2 μg/mL (5-FU)
	A2780	8.5 μg/mL [16]	0.7 μg/mL (5-FU)
	Bel-7402	n.a. [16]	0.5 μg/mL (5-FU)
	BGC-823	n.a. [16]	0.7 μg/mL (5-FU)
	HCT-8	n.a. [16]	0.5 μg/mL (5-FU)
Guangsangon E (**56**)	P-388	2.75 μg/mL [34]; 2.5 μg/mL [26]	–
Sorocein I (**65**)	P-388	n.a. [34]	–
Kuwanon J (**81**)	Hep3B	8.55 μM [71]	> 30 μM (ALV)
	P-388	5.9 μg/mL [26]	–
	HIF-1*	4.10 μM [71]	0.0572 μM (ALV)
	VEGF*	3.14 μM [71]	0.0795 μM (ALV)
Kuwanon Q (**83**)	Hep3B	5.94 μM [71]	> 30 μM (ALV)
	HIF-1*	3.80 μM [71]	0.0572 μM (ALV)
	VEGF*	4.24 μM [71]	0.0795 μM (ALV)
Kuwanon R (**86**)	Hep3B	6.42 μM [71]	> 30 μM (ALV)
	HIF-1*	3.17 μM [71]	0.0572 μM (ALV)
	VEGF*	3.51 μM [71]	0.0795 μM (ALV)
Kuwanon V (**87**)	Hep3B	9.54 μM [71]	> 30 μM (ALV)
	HIF-1*	8.32 μM [71]	0.0572 μM (ALV)
	VEGF*	7.84 μM [71]	0.0795 μM (ALV)
Kuwanon G (**92**)	A549	n.a. [16]	0.2 μg/mL (5-FU)
	A2780	n.a. [16]	0.7 μg/mL (5-FU)
	Bel-7402	n.a. [16]	0.5 μg/mL (5-FU)
	BGC-823	n.a. [16]	0.7 μg/mL (5-FU)
	HCT-8	n.a. [16]	0.5 μg/mL (5-FU)
	HepG2	34.35 μM [76]	41.55 μM (CUR)
	MCF-7	35.79 μM [76]	34.17 μM (5-FU)
Australisine A (**113**)	A549	n.a. [16]	0.2 μg/mL (5-FU)
	A2780	8.2 μg/mL [16]	0.7 μg/mL (5-FU)
	Bel-7402	n.a. [16]	0.5 μg/mL (5-FU)
	BGC-823	n.a. [16]	0.7 μg/mL (5-FU)
	HCT-8	9.2 μg/mL [16]	0.5 μg/mL (5-FU)
Sanggenol M (**126**)	HSC-2	13 μM [122]	58 μM (2′-HC)
	HSG	13 μM [122]	49 μM (2′-HC)
Sanggenon D (**128**)	HSC-2	44 μM [122]	58 μM (2′-HC)
	HSG	64 μM [122]	49 μM (2′-HC)
Sanggenon C (**130**)	Hep3B	8.26 μM [71]	> 30 μM (ALV)
	HSC-2	18 μM [122]	58 μM (2′-HC)
	HSG	23 μM [122]	49 μM (2′-HC)
	HIF-1*	1.26 μM [71]	0.0572 μM (ALV)
	VEGF*	3.20 μM [71]	0.0795 μM (ALV)
Sanggenon O (**131**)	Hep3B	8.75 μM [71]	> 30 μM (ALV)
	HIF-1*	1.03 μM [71]	0.0572 μM (ALV)
	VEGF*	2.08 μM [71]	0.0795 μM (ALV)
Soroceal B (**146**)	A549	> 40 μM [13]	12.4 μM (CPT)
	AGS	15.8 μM [13]	18.8 μM (CPT)
	Hela	18.7 μM [13]	4.9 μM (CPT)
	HepG2	> 40 μM [13]	12.5 μM (CPT)
	HL-60	17.7 μM [13]	3.7 μM (CPT)
Mulberrofuran H (**162**)	Hep3B	> 30 μM [71]	> 30 μM (ALV)
	HIF-1	0.14 μM [71]	0.0572 μM (ALV)
	VEGF	15.4 μM [71]	0.0795 μM (ALV)
Sanggenon B (**165**)	HSC-2	39 μM [122]	58 μM (2′-HC)
	HSG	47 μM [122]	49 μM (2′-HC)

^a^Target proteins: HIF-1*, Hypoxia-inducible factor-1; VEGF*, Vascular endothelial growth factor. ^b^*2′-HC* 2′-Hydroxychalcone, *5-FU *5-Fluorouracil; *ALV* Alvespimycin (17-DMAG); *CPT* Cisplatin, *CUR* Curcumin, *ETO* Etoposide

Hypoxia-inducible factor-1 (HIF-1) inhibitor represents a promising anti-cancer agent. The eight MDAAs mulberrofuran G (**31**), kuwanon J (**81**), kuwanon Q (**83**), kuwanon R (**86**), kuwanon V (**87**), sanggenon C (**130**), sanggenon O (**131**), and mulberrofuran H (**162**) were reported for the first times to possess HIF-1 inhibitory effect in the Hep3B cell-based assay [71]. Additionally, these compounds were also active against hypoxia-induced vascular endothelial growth factor (VEGF) secretion in Hep3B cells (Table 3) [71].

### Anti-inflammation activity

The Yu group have evaluated the inhibition effects of the obtained MDAAs (at a concentration of 10 μM) on the release of *β*-glucuronidase in rat polymorphonuclear (PMN) leukocytes induced by PAF and found that mongolicins C and E (**24** and **152**) showed potent anti-inflammatory activities with inhibitory ratios of 80.4% and 77.0% [25], respectively, while albafuran C (**50**), guangsangon J (**51**), guangsangon B (**75**), guangsangon I (**106**), and guangsangon H (**121**) displayed moderate effects [17, 85, 149]. Kimura et al. have found that mulberrofuran J (**1**), mulberrofuran Q (**30**), mulberrofuran G (**31**), kuwanon G (**92**), kuwanon H (**93**), sanggenon D (**128**), and sanggenon C (**130**) could affect arachidonate metabolism in rat platelets [150]. Kuwanon G (**92**) and kuwanon O (**119**) could significantly down-regulated the expressions of TNF-*α*, IL-1*β*, IL-6, COX-2, and NF-*κ*B in dose-dependent manners in LPS-stimulated RAW264 cells [107]. Among albanol B (**46**), sanggenon D (**128**), and sanggenon B (**165**), **46** exhibited the best anti-inflammatory activity, which inhibited expression of inducible nitric oxide synthase (iNOS) and cyclooxygenase-2 (COX-2) and suppressed production of pro-inflammatory cytokines and mediators in LPS-induced RAW264.7 cells [81]. Mulberrofuran K (**40**) suppressed the production of NO, reactive oxygen species (ROS), and proinflammatory cytokines IL-1*β*, IL-6, and TNF-*α* in a dose-dependent manner in LPS-stimulated RAW264 cells [73]. Cathayanons A and B (**136** and **137**) displayed potent activities on the inhibition of HL-60 cell adhesion to BAEC (Bovine Arterial Endothalium cells) at 10 μM, with inhibitory rates of 44.72% and 39.02%, respectively [130]. Among the six MDAAs mulberrofuran J (**1**), mulberrofuran C (**6**), mongolicin C (**24**), mulberrofuran G (**31**), artonin I (**101**), and soroceal B (**146**), only **1** (IC_50_ = 21.4 μM) and **24** (IC_50_ = 8.8 μM) exhibited moderate inhibitory activity against NO production in LPS-activated RAW264.7 cells [13]. In TNF-*α*-stimulated HeLa cells, kuwanon J 2,4,10"-trimethyl ether (**82**) and kuwanon R (**86**) strongly inhibited NF-*κ*B activity with the IC_50_ values of 4.65 and 7.38 μM, respectively [100]. Sanggenons C and O (**130** and **131**) could inhibit NO production and iNOS expression by suppressing NF-*κ*B activity and I*κ*B*α* activation in LPS-stimulated RAW264 cells [151]. In a high-throughput screening for ADAMTS1 (a disintegrin and metalloprotease with thrombospondin type I motifs-1) inhibitors by the fluorescence resonance energy transfer (FRET) method, four compounds mulberrofuran J (**1**), albafuran C (**50**), kuwanon X (**58**), and kuwanon P (**76**) were identified from a diverse library of 40,960 total compounds [152]. The results of kuwanon G (**92**) on ovalbumin (OVA)-induced allergic asthma in mice indicated that it could prevent the pathological progression of allergic asthma through the inhibition of lung destruction by inflammation and immune stimulation [153]. Kuwanon G (**92**) could inhibit chemokine production by blocking of STAT1 (signal transducer and activator of transcription 1) and NF-*κ*B pathways in HaCaT keratinocytes and reduce the release of histamine and LTC_4_ (leukotriene C_4_) by suppressing the 5-LO (5-lipoxygenase) activation in MC/9 mast cells, which suggested it had anti-allergic and anti-inflammatory effects [154]. Among the five MDAAs sanggenons B − E and O, sanggenon E (**129**), sanggenon C (**130**), and sanggenon O (**131**) had inhibitory effects on two cyclooxygenase isoenzymes COX-1 and -2 with IC_50_ values of 10 − 14 μM and 40 − 50 μM, respectively [127].

### Antibacterial activity

Fukai et al. found that chalcomoracin (**5**), mulberrofuran G (**31**), mulberrofuran F (**32**), and albanol B (**46**) showed considerable antibacterial activity against several vancomycin-resistant enterococci (VRE) and methicillin-resistant *Staphylococcus aureus* (MRSA) strains with MIC values ranging from 3.13 to 6.25 μg/mL. Among chalcomoracin (**5**), mulberrofuran G (**31**), mulberrofuran F (**32**), and albanol B (**46**), compound **5** (MIC = 0.78 μg/mL) showed antimicrobial activity comparable to that of positive drugs against MRSA (strains K3 and ST28) and methicillin-sensitive *Staphylococcus aureus* (MSSA) (strains FDA 209P and Smith), while **32** and **46** showed slightly weak antimicrobial activity against these MRSAs and MSSAs (MICs = 0.78–1.56 μg/mL) (Table 4) [155]. In addition to anti *Staphylococcus aureus* including MRSA, chalcomoracin (**5**) could strongly inhibited *S. aureus* enoyl-ACP reductase (saFabI) with a IC_50_ of 5.5 μM, a novel target for antibacterial drug development [30, 156]. Among the four MDAAs mulberrofuran G (**31**), albanol B (**46**), sanggenon D (**128**), and sanggenon B (**165**), compounds **31** and **46** showed strong antibacterial activity against *Escherichia coli*, *Salmonella typhimurium*, *Staphylococcus epidermis*, and *Staphylococcus aureus* with 5–30 μg/mL of MICs, while **128** and **165** were effective to *Saccaromyces cerevisiae*, *Staphylococcus epidermis*, and *Staphylococcus aureus* with 12.5–50 μg/mL of MICs [157]. Except for kuwanol A (**64**) and kuwanon L (**118**), all seven MDAAs mulberrofuran Q (**30**), mulberrofuran G (**31**), mulberrofuran K (**40**), albanol B (**46**), kuwanon G (**92**), kuwanon H (**93**), and kuwanon O (**119**) had better bioactivity against MRSA both in vitro and in *vivo* than antibiotics such as berberine, ampicillin, and kanamycin. Preliminary mechanism of action showed that these compounds could damage the bacterial cell membranes as well as inhibit the efflux of drugs such as methicillin and ethidium bromide [62]. Among the six MDAAs albanol B (**46**), albafuran C (**50**), kuwanon G (**92**), kuwanon H (**93**), kuwanon K (**109**), and kuwanon L (**118**), compounds **93** and **109** diminished the growth of a standard strain *E. faecalis*, three clinical isolates of VRE, and three clinical isolates of MRSA, with MIC values of 1–32 μg/mL [82]. Kuwanon G (**92**) significantly inhibited the growth of the cariogenic bacteria such as *Streptococcus mutans* (MIC = 8 μg/mL), *Streptococcus sobrinus* (MIC = 8 μg/mL), and *Streptococcus sanguis* (MIC = 8 μg/mL), and periodontal bacterium, *Porpyromonas gingivalis* (MIC = 8 μg/mL). Transmission electron microscopy (TEM) showed that **92** could remarkably cause morphological damage of the cell wall and condensation of the cytoplasm in *S. mutans* [106]. Wu et al. found that kuwanon G (**92**) and kuwanon H (**93**) could treat MRSA-associated infections by disrupting the proton motive force and membrane permeability [108]. Kuwanon G (**92**) was also identified as a good candidate for the development of novel antibacterial combination therapy [158, 159]. Kuwanon H (**93**), multicaulisin (**99**), and sanggenon G (**124**) were found to possess potent effect against ten clinical MRSA strains with MICs/MBCs of 2–8/16–128 mg/L, and also showed synergy with conventional antibacterial agents such as aminoglycosides [111]. Artonin I (**101**) could inhibit multidrug resistance in *Staphylococcus aureus* and potentiate the action of inactive antibiotics in vitro [116]. From the five MDAAs kuwanon L (**118**), sanggenon G (**124**), sanggenon D (**128**), sanggenon C (**130**), and sanggenon B (**165**), compound **124** was discovered to be a potent antibacterial agent against *Streptococcus pneumoniae* with a MIC of 5.44 μM [160]. Pang et al. found that sanggenon D (**128**) could inhibit the growth of *Staphylococcus aureus* by moderating the fatty acid biosynthesis system [161].

**Table 4:** Antibacterial activities of MDAAs

Compd	Bacterium^a^	Activity (MIC or MIC/MBC in μg/mL)	Positive control (MIC or MIC/MBC in μg/mL)^b^
Chalcomoracin (**5**)	*B. subtilis* PCI219	1.56 [155]	0.10 (AMX); 0.39 (VAN)
	*E. faecium* JCM 5804	6.25 [156]	0.78 (VAN); 1.56 (LNZ)
	*E. faecalis* JCM 7783	3.13 [156]	3.13 (VAN); 1.56 (LNZ)
	*E. faecium* JU 1777	3.13 [156]	100 (VAN); 1.56 (LNZ)
	*E. faecalis* JU 1782	3.13 [156]	100 (VAN); 0.78 (LNZ)
	*E. faecalis* JU 1856	3.13 [156]	100 (VAN); 0.78 (LNZ)
	*E. faecium* JU 1858	3.13 [156]	200 (VAN); 0.78 (LNZ)
	*E. gallinarum* JU 2786	3.13 [156]	12.5 (VAN); 0.78 (LNZ)
	*M. luteus* ATCC9341	1.56 [155]	0.025 (AMX); 1.56 (VAN)
	MRSA (10 strains)	3.13 [156]	0.78 − 1.56 (VAN); 50 − > 200 (OXA); 0.78 (LNZ)
	MRSA CCARM3167	2 [30]	-
	MRSA K3	0.78 [155]	25 (AMX); 1.56 (VAN)
	MRSA ST28	0.78 [155]	50 (AMX); 0.78 (VAN)
	MSSA ATCC29213	3.13 [156]	0.78 (VAN); 0.20 (OXA); 1.56 (LNZ)
	MSSA FDA209	0.78 [155]	0.10 (AMX); 0.39 (VAN)
	MSSA Smith	0.78 [155]	0.10 (AMX); 1.56 (VAN)
	*S. aureus* RN4220	4 [30]	-
Mulberrofuran Q (**30**)	MRSA 003	16/32 [62]	4/8 (KAN); 16/32 (AMP); 256/512 (MET); 256/512(BER)
	MRSA 011	8/16 [62]	4/4 (KAN); 8/16 (AMP); 128/256 (MET); 128/256 (BER)
	MRSA 031	16/32 [62]	4/8 (KAN); 16/32 (AMP); 256/512 (MET); 128/256 (BER)
	*S. aureus* ATCC4330	8/8 [62]	2/4 (KAN); 4/16 (AMP); 32/64 (MET); 64/128 (BER)
Mulberrofuran G (**31**)	*B. subtilis* PCI219	3.13 [155]	0.10 (AMX); 0.39 (VAN)
	*C. albicans*	> 60 [157]	1.25 (AMB); 1.25 (MIZ)
	*E. faecium* JCM 5804	6.25 [156]	0.78 (VAN); 1.56 (LNZ)
	*E. faecalis* JCM 7783	6.25 [156]	3.13 (VAN); 1.56 (LNZ)
	*E. faecium* JU 1777	3.13 [156]	100 (VAN); 1.56 (LNZ)
	*E. faecalis* JU 1782	3.13 [156]	100 (VAN); 0.78 (LNZ)
	*E. faecalis* JU 1856	3.13 [156]	100 (VAN); 0.78 (LNZ)
	*E. faecium* JU 1858	3.13 [156]	200 (VAN); 0.78 (LNZ)
	*E. gallinarum* JU 2786	6.25 [156]	12.5 (VAN); 0.78 (LNZ)
	E.* *coli*	30 [157]	1.25 (AMP); 1.25 (ERY)
	*M. luteus* ATCC9341	1.56 [155]	0.025 (AMX); 1.56 (VAN)
	MRSA (10 strains)	3.13 [156]	0.78 − 1.56 (VAN); 50 − > 200 (OXA); 0.78 (LNZ)
	MRSA 003	16/32 [62]	4/8 (KAN); 16/32 (AMP); 256/512 (MET); 256/512(BER)
	MRSA 011	8/16 [62]	4/4 (KAN); 8/16 (AMP); 128/256 (MET); 128/256 (BER)
	MRSA 031	8/16 [62]	4/8 (KAN); 16/32 (AMP); 256/512 (MET); 128/256 (BER)
	MRSA K3	1.56 [155]	25 (AMX); 1.56 (VAN)
	MRSA ST28	3.13 [155]	50 (AMX); 0.78 (VAN)
	MSSA ATCC29213	3.13 [156]	0.78 (VAN); 0.20 (OXA); 1.56 (LNZ)
	MSSA FDA209	1.56 [155]	0.10 (AMX); 0.39 (VAN)
	MSSA Smith	1.56 [155]	0.10 (AMX); 1.56 (VAN)
	S.* cerevisiae	> 60 [157]	1.25 (AMB); 1.25 (MIZ)
	S.** typhimurium	7.5 [157]	1.25 (AMP); 5 (ERY)
	*S. aureus*	5 [157]	1.25 (AMP); 1.25 (ERY)
	*S. aureus* ATCC4330	8/16 [62]	2/4 (KAN); 4/16 (AMP); 32/64 (MET); 64/128 (BER)
	*S. epidermis*	6.25 [157]	20 (AMP); 1.25 (ERY)
Mulberrofuran F (**32**)	*B. subtilis* PCI219	6.25 [155]	0.10 (AMX); 0.39 (VAN)
	*E. faecium* JCM 5804	6.25 [156]	0.78 (VAN); 1.56 (LNZ)
	*E. faecalis* JCM 7783	6.25 [156]	3.13 (VAN); 1.56 (LNZ)
	*E. faecium* JU 1777	3.13 [156]	100 (VAN); 1.56 (LNZ)
	*E. faecalis* JU 1782	3.13 [156]	100 (VAN); 0.78 (LNZ)
	*E. faecalis* JU 1856	3.13 [156]	100 (VAN); 0.78 (LNZ)
	*E. faecium* JU 1858	6.25 [156]	200 (VAN); 0.78 (LNZ)
	*E. gallinarum* JU 2786	3.13 [156]	12.5 (VAN); 0.78 (LNZ)
	*M. luteus* ATCC9341	1.56 [155]	0.025 (AMX); 1.56 (VAN)
	MRSA (10 strains)	3.13 [156]	0.78 − 1.56 (VAN); 50 − > 200 (OXA); 0.78 (LNZ)
	MRSA K3	1.56 [155]	25 (AMX); 1.56 (VAN)
	MRSA ST28	1.56 [155]	50 (AMX); 0.78 (VAN)
	MSSA ATCC29213	3.13 [156]	0.78 (VAN); 0.20 (OXA); 1.56 (LNZ)
	MSSA FDA209	0.78 [155]	0.10 (AMX); 0.39 (VAN)
	MSSA Smith	1.56 [155]	0.10 (AMX); 1.56 (VAN)
Mulberrofuran K (**40**)	MRSA 003	8/16 [62]	4/8 (KAN); 16/32 (AMP); 256/512 (MET); 256/512(BER)
	MRSA 011	4/8 [62]	4/4 (KAN); 8/16 (AMP); 128/256 (MET); 128/256 (BER)
	MRSA 031	8/16 [62]	4/8 (KAN); 16/32 (AMP); 256/512 (MET); 128/256 (BER)
	*S. aureus* ATCC4330	4/8 [62]	2/4 (KAN); 4/16 (AMP); 32/64 (MET); 64/128 (BER)
Albanol B (**46**)	*B. subtilis* PCI219	1.56 [155]	0.10 (AMX); 0.39 (VAN)
	*C. albicans*	> 60 [157]	1.25 (AMB); 1.25 (MIZ)
	*E. faecium* JCM 5804	6.25 [156]	0.78 (VAN); 1.56 (LNZ)
	*E. faecalis* JCM 7783	3.13 [156]	3.13 (VAN); 1.56 (LNZ)
	*E. faecium* JU 1777	3.13 [156]	100 (VAN); 1.56 (LNZ)
	*E. faecalis* JU 1782	3.13 [156]	100 (VAN); 0.78 (LNZ)
	*E. faecalis* JU 1856	3.13 [156]	100 (VAN); 0.78 (LNZ)
	*E. faecium* JU 1858	3.13 [156]	200 (VAN); 0.78 (LNZ)
	*E. gallinarum* JU 2786	3.13 [156]	12.5 (VAN); 0.78 (LNZ)
	E.* coli	20 [157]	1.25 (AMP); 1.25 (ERY)
	*M. luteus* ATCC9341	1.56 [155]	0.025 (AMX); 1.56 (VAN)
	MRSA (10 strains)	3.13 [156]	0.78 − 1.56 (VAN); 50 − > 200 (OXA); 0.78 (LNZ)
	MRSA 003	16/32 [62]	4/8 (KAN); 16/32 (AMP); 256/512 (MET); 256/512(BER)
	MRSA 011	8/16 [62]	4/4 (KAN); 8/16 (AMP); 128/256 (MET); 128/256 (BER)
	MRSA 031	8/16 [62]	4/8 (KAN); 16/32 (AMP); 256/512 (MET); 128/256 (BER)
	MRSA K3	1.56 [155]	25 (AMX); 1.56 (VAN)
	MRSA ST28	1.56 [155]	50 (AMX); 0.78 (VAN)
	MSSA ATCC29213	3.13 [156]	0.78 (VAN); 0.20 (OXA); 1.56 (LNZ)
	MSSA FDA209	1.56 [155]	0.10 (AMX); 0.39 (VAN)
	MSSA Smith	1.56 [155]	0.10 (AMX); 0.1.56 (VAN)
	S.* cerevisiae	> 60 [157]	1.25 (AMB); 1.25 (MIZ)
	S.** typhimurium	5 [157]	1.25 (AMP); 5 (ERY)
	*S. aureus*	5 [157]	1.25 (AMP); 1.25 (ERY)
	*S. aureus* ATCC4330	8/8 [62]	2/4 (KAN); 4/16 (AMP); 32/64 (MET); 64/128 (BER)
	*S. epidermis*	5 [157]	20 (AMP); 1.25 (ERY)
Kuwanon G (**92**)	*B. subtilis* ATCC6051	4 [108]	≤ 0.25 (VAN)
	*E. faecalis* VRE1010798	4 [108]	> 128 (VAN)
	*P. gingivalis* W50	8 [106]	-
	MRSA 003	4/8 [62]	4/8 (KAN); 16/32 (AMP); 256/512 (MET); 256/512(BER)
	MRSA 011	4/4 [62]	4/4 (KAN); 8/16 (AMP); 128/256 (MET); 128/256 (BER)
	MRSA 031	4/4 [62]	4/8 (KAN); 16/32 (AMP); 256/512 (MET); 128/256 (BER)
	MRSA T144	2 [108]	2 (VAN)
	MSSA ATCC29123	2 [108]	2 (VAN)
	*S. aureus* ATCC10231	125 [106]	-
	*S. aureus* ATCC4330	2/4 [62]	2/4 (KAN); 4/16 (AMP); 32/64 (MET); 64/128 (BER)
	S.*** mutans ATCC25175	8 [106]	1 (VAN)
	S.*** sangui ATCC35105	8 [106]	-
	S.*** sobrinus ATCC27351	8 [106]	-
Kuwanon H (**93**)	*B. subtilis* ATCC6051	2 [108]	≤ 0.25 (VAN)
	*E* *faecalis* ATCC 29,212	16/32 [82]	8/8 (AMP)
	*E. faecalis* VRE1010798	4 [108]	> 128 (VAN)
	MSSA ATCC25923	4/32 [111]	0.5/2 (VAN)
	MRSA (ten clinical strains)	2 − 16/32 − 128 [111]	0.25 − 2/1 − 2 (VAN)
	MRSA 003	2/4 [62]	4/8 (KAN); 16/32 (AMP); 256/512 (MET); 256/512(BER)
	MRSA 011	2/2 [62]	4/4 (KAN); 8/16 (AMP); 128/256 (MET); 128/256 (BER)
	MRSA 031	8/4 [62]	4/8 (KAN); 16/32 (AMP); 256/512 (MET); 128/256 (BER)
	MRSA 630	2/2 [82]	128/256 (CIP)
	MRSA 3202	1/2 [82]	16/16 (CIP)
	MRSA 63,718	2/4 [82]	8/16 (CIP)
	MRSA T144	1 [108]	2 (VAN)
	MSSA ATCC29123	2 [108]	2 (VAN)
	*S. aureus* ATCC4330	2/4 [62]	2/4 (KAN); 4/16 (AMP); 32/64 (MET); 64/128 (BER)
	VRE 342B	32/32 [82]	8/16 (AMP)
	VRE 368	32/32 [82]	8/8 (AMP)
	VRE 725B	32/32 [82]	8/8 (AMP)
Multicaulisin (**99**)	MRSA (ten clinical strains)	2 − 8/16 − 32 [111]	0.25 − 2/1 − 2 (VAN)
	MSSA ATCC25923	4/16 [111]	0.5/2 (VAN)
Kuwanon K (**109**)	*E* *faecalis* ATCC 29,212	8/16 [82]	8/8 (AMP)
	MRSA 630	4/4 [82]	128/256 (CIP)
	MRSA 3202	4/4 [82]	16/16 (CIP)
	MRSA 63,718	4/4 [82]	8/16 (CIP)
	VRE 342B	16/16 [82]	8/16 (AMP)
	VRE 368	16/16 [82]	8/8 (AMP)
	VRE 725B	16/32 [82]	8/8 (AMP)
Kuwanon L (**118**)	S.*** pneumoniae DSM20566	31.6 μM [160]	2.08 μM (OSE)
Kuwanon O (**119**)	MRSA 003	8/16 [62]	4/8 (KAN); 16/32 (AMP); 256/512 (MET); 256/512(BER)
	MRSA 011	16/16 [62]	4/4 (KAN); 8/16 (AMP); 128/256 (MET); 128/256 (BER)
	MRSA 031	8/16 [62]	4/8 (KAN); 16/32 (AMP); 256/512 (MET); 128/256 (BER)
	*S. aureus* ATCC4330	4/8 [62]	2/4 (KAN); 4/16 (AMP); 32/64 (MET); 64/128 (BER)
Sanggenon G (**124**)	MRSA (ten clinical strains)	4/8 − 16 [111]	0.25 − 2/1 − 2 (VAN)
	MSSA ATCC25923	4/8 [111]	0.5/2 (VAN)
	S.*** pneumoniae DSM20566	5.44 μM [160]	2.08 μM (OSE)
Sanggenon D (**128**)	*C. albicans*	> 60 [157]	1.25 (AMB); 1.25 (MIZ)
	E.* coli	141.1 μM [161]	17.9 μM (AMP)
	S.* cerevisiae	25 [157]	1.25 (AMB); 1.25 (MIZ)
	S.** typhimurium	40 [157]	1.25 (AMP); 5 (ERY)
	*S. aureus*	20 [157]	1.25 (AMP); 1.25 (ERY)
	*S. aureus*	17.6 μM [161]	9.0 μM (AMP)
	*S. epidermis*	50 [157]	20 (AMP); 1.25 (ERY)
	S.*** pneumoniae DSM20566	31.6 μM [160]	2.08 μM (OSE)
Sanggenon C (**130**)	S.*** pneumoniae DSM20566	n.a [160]	2.08 μM (OSE)
Sanggenon B (**165**)	*C. albicans*	> 60 [157]	1.25 (AMB); 1.25 (MIZ)
	E.* coli	> 100 [157]	1.25 (AMP); 1.25 (ERY)
	S.* cerevisiae	25 [157]	1.25 (AMB); 1.25 (MIZ)
	S.** typhimurium	> 100 [157]	1.25 (AMP); 5 (ERY)
	*S. aureus*	15 [157]	1.25 (AMP); 1.25 (ERY)
	*S. epidermis*	12.5 [157]	20 (AMP); 1.25 (ERY)
	S.*** pneumoniae DSM20566	31.6 μM [160]	2.08 μM (OSE)

^a^*B.*, *Bacillus*; *C.*, *Candida*; *E.*, *Enterococcus*; E.*, *Escherichia*; *M.*, *Micrococcus*; *P.*, *Porphlococcus*; *S.*, *Staphylococcus*; S.*, *Saccaromyces*; S.**, *Salmonella*; S.***, *Streptococcus*; MRSA, methicillin-resistant *S. aureus*; MSSA, methicillin-sensitive *S. aureus*; VRE, vancomycin-resistant enterococci. ^b^*AMB* Amphotericin B, *AMP* Ampicillin, *AMX* Amoxicillin, *BER* Berberine, *CIP* Ciprofloxacin, *ERY* Erythromycin, *KAN* Kanamycin, *LNZ* Linezolid, *MET* Methicillin, *MIZ* Miconazole, *OSE* Oseltamivir, *OXA* Oxacillin, *VAN* Vancomycin

Protein tyrosine phosphatase B (PtpB) is a promising target for the development of novel anti-tuberculosis drugs. From an *in house* library of more than 800 natural substances, kuwanol E (**62**) was discovered by Mascarello et al. to be the most potent *Mycobacterium tuberculosis* PtpB inhibitor (IC_50_ = 1.9 μM and *K*_i_ = 1.6 μM) [162]. Subsequently, Mascarello et al. found that among the four MDAAs chalcomoracin (**5**), kuwanon G (**92**), kuwanon H (**93**), and kuwanon L (**118**), compounds **92** (IC_50_ = 0.83 μM) and **93** (IC_50_ = 0.36 μM) were the two most potent Mtb PtpB inbibitors with *K*_i_ values of 0.39 and 0.20 μM, respectively. The comprehensive research strategies including kinetics, mass spectrometry, and molecular docking demonstrated that both compounds were interacted with the active site of the enzyme [37]. Kuwanol E (**64**), kuwanon G (**92**), and kuwanon H (**93**) are the first non-peptidic PtpB inhibitors discovered from natural sources.

### Antioxidant activity

MDAAs are phenolic natural products with multiple hydroxyl groups, which contribute their strong antioxidant properties. The Yu and Chen groups evaluated the antioxidant properties of their obtained MDAAs in Fe^2+^/cysteine-induced microsomal lipid peroxidation assay and found that most of the compounds at concentrations of 10 μM had good activities (Vitamine E as the positive control) [17, 20, 22, 25, 49, 50, 74, 78, 85, 91, 119, 149]. For example, guangsangon J (**51**), guangsangon I (**106**), and guangsangon H (**121**) were first reported to display potent antioxidant activities with the inhibitory rates of malondialdehyde being 91.1%, 93.9%, and 93.1%, respectively, compared to the positive control Vit E (33.4%) [17]. The other active MDAAs were listed in Table 5. In addition, kuwanol E (**62**) exhibited remarkable free radical scavenging properties with the IC_50_ value of 2.1 μg/mL (the standard trolox, IC_50_ = 1.1 μg/mL) [27]. Li et al. found that sanggenon C (**130**) and sanggenon D (**128**) may undergo an antioxidant approach to protect mesenchymal stem cells (MSCs) against oxidative stress, and the discrepancies in their antioxidant activities could be attributed to the steric effect [163].

**Table 5:** Antioxidant activities of MDAAs at concentrations of 10 μM

Compd.^a^	Activity (inhibitory ratio)	Positive control (vitamine E, inhibitory ratio)
Mulberrofuran J (**1**)	91.2% [20]	66.7%
Mongolicin F (**2**)	86.0%, p < 0.05 [25]	
Mulberrofuran Q (**30**)	85.6% [20]	66.7%
Mulberrofuran F (**32**)	100% [49]	67%
Mulberrofuran K (**40**)*	92.1% [50]	35.9%
Yunanensin D (**43**)	67.9% [78]	70.1%
Albanol B (**46**)*	100.0% [50]	35.9%
Albafuran C (**50**)	76.2% [85]	33.5%
Guangsangon J (**51**)	91.1% [17]	33.4%
Guangsangon A (**52**)	84.9% [85]	33.5%
Yunanensin B (**54**)	57.1% [78]	70.1%
Guangsangon E (**56**)	88.1% [85]	33.5%
Kuwanon X (**58**)	80.8% [85]	33.5%
Kuwanon Y (**61**)	70.9% [85]	33.5%
Sorocein A (**68**)	100% [49]	67%
Cathayanon C (**70**)	95% [91]	62.6%
Cathayanon D (**71**)	103% [91]	62.6%
Guangsangon B (**75**)	83.7% [85]	33.5%
Guangsangon C (**91**)	81.7% [85]	33.5%
Guangsangon I (**106**)	93.9% [17]	33.4%
Wittiorumin A (**107**)	73.0% [22]	62.5%
Wittiorumin B (**108**)	82.0% [22]	62.5%
Guangsangon O (**111**)	94.8% [119]	31.9%
Wittiorumin C (**112**)	82.0% [22]	62.5%
Guangsangon M (**114**)	98.3% [74]	18.2%
Guangsangon N (**115**)	100.0% [74]	18.2%
Kuwanon O (**119**)	63.0%, p < 0.05 [25]	
Guangsangon D (**120**)	77.6% [85]	33.5%
Guangsangon H (**121**)	93.1% [17]	33.4%
Guangsangon K (**122**)	91.8% [74]	18.2%
Guangsangon L (**145**)	97.6% [74]	18.2%
Mongolicin B (**161**)	83.6%, p < 0.05 [25]	

^a^*The working concentration of these samples including positive control vitamine E was 10 mg/L

### Anti-neurodegenerative diseases

Ten MDAAs, mulberrofuran J (**1**), mulberrofuran C (**6**), inethermulberrofuran C (**20**), mulberrofuran G (**31**), mulberrofuran K (**40**), albafuran C (**50**), isomulberrofuran G (**57**), kuwanol A (**64**), kuwanon G (**92**), and kuwanon H (**93**), were systematically screened for their anti-Alzheimer's disease (anti-AD) properties on different targets such as tau aggregation, A*β* self-aggregation, and ChEs. Of these compounds, **6**, **31**, **40**, and **57** were found to be potent multi-targeted agents for AD. The selected mulberrofuran K (**40**) with a good blood–brain barrier (BBB) permeability could play neuroprotective effects by up-regulating the level of glutathione (GSH) and inhibiting the production of reactive oxygen species (ROS) in glutamate-induced HT22 cell model [15]. Among the three MDAAs mulberrofuran C (**6**), mulberrofuran G (**31**), and sanggenon G (**124**), compounds **6** and **31** were found to have neuroprotective activity on glutamate-induced cell death in HT22 cells with EC_50_ values of 19.71 and 16.50 μM, respectively [44]. Mulberrofuran Q (**30**) showed inhibitory activity against oxygen glucose deprivation (OGD)-induced cell death of SH-SY5Y cells, and its protective effect was more potent than the positive control carnosine [60]. Hong et al. have investigated the neuroprotective effect of mulberrofuran G (**31**) in in vitro and in vivo models of cerebral ischemia. It was found that **31** could protect ischemic injury-induced cell death through the inhibition of NOX4-mediated ROS generation and ER stress [72]. Mulberrofuran G (**31**), albanol B (**46**), and kuwanon G (**92**) displayed potent inhibitory activities against ChEs and *β*-site amyloid precursor protein cleaving enzyme 1 (BACE1) [67]. Later, mulberrofuran G (**31**), albanol B (**46**), and kuwanon G (**92**) were identified as inhibitors of human monoamine oxidase (*h*MAO) and modulators of dopaminergic receptor [164]. Kuwanon V (**87**) could inhibit the proliferation of neural stem cells, promote cell survival, and increase neurogenesis [103]. Moracenin D (**95**) was found to possess protective effects in dopamine-induced SH-SY5Y cells via the up-regulation of nurr1 and down-regulation of *α*-synuclein expressions [165]. Zhao et al. demonstrated that sanggenon C (**130**) had neuroprotective effects on ischemic stroke by inhibiting inflammation and oxidative stress by regulating RhoA-ROCK signaling pathway [166]. The neuroprotective activity of morusalbanol A (**148**) in H_2_O_2_-induced PC12 cells was evaluated, and the result showed that pretreatment with **148** could significantly attenuate the H_2_O_2_-induced cell damage in a dose-dependent manner [134].

### PTP1B inhibitory activity

Protein tyrosine phosphatase 1B (PTP1B) is a key negative regulator of insulin and leptin signaling pathways. It is currently considered as a valid therapeutic target for type 2 diabetes mellitus and obesity [167]. In 2006, Cui et al. first found four MDAAs mulberrofuran C (**6**), kuwanon L (**118**), sanggenon G (**124**), and sanggenon C (**130**) showed significant PTP1B inhibitory activities with IC_50_ values of 4.9, 16.9, 1.6, and 2.6 μM, respectively. Analysis of inhibition kinetics by Lineweaver–Burk plots suggested that the inhibition mode of the most active compounds (**6**, **124**, and **130**) on the activity of PTP1B was mixed-type [43]. Subsequently, kuwanon J (**81**), kuwanon R (**86**), and kuwanon V (**87**) were indentified as mixed-type inhibitors of PTP1B with IC_50_ values ranging from 2.7 to 13.8 μM [98]. Since 2015, a large number of MDAAs have been reported to have significant inhibitory activity against PTP1B (Table 6) [14, 39, 46, 51, 61, 137, 168]. Mechanism research suggested that the potent PTP1B inhibitors ( +)-morusalone A (**154**) and morusalone C (**158**) could significantly increase the insulin induced phosphorylation of IRβ in HepG2 cells, and then upregulate the phosphorylation level of downstream Akt [137].

**Table 6:** Inhibitory activities of MDAAs against PTP1B

Compd.	Activity (IC_50_ in μM); *K*_i_ value; Inhibition type	Positive control (IC_50_ in μM)^a^
Mulberrofuran J (**1**)	0.60 [14]	3.31 (OA)
Albasin A (**3**)	1.59 [14]	3.31 (OA)
Chalcomoracin (**5**)	1.9 [39]	3.0 (OA)
Mulberrofuran C (**6**)	4.9 [43]	4.5 (RK)
	0.72 [14]	3.31 (OA)
Albasin B (**8**)	5.12 [14]	3.31 (OA)
	2.80; *K*_i_ = 1.00 μM; Noncompetitive [46]	9.47 (UA)
Morusalisin C (**10**)	1.58 [51]	1.70 (CCF)
Morbilisin G (**14**)	2.0 [39]	3.0 (OA)
Morbilisin H (**15**)	1.5 [39]	3.0 (OA)
Morusalisin D (**16**)	1.52 [51]	1.70 (CCF)
Morusalisin E (**17**)	1.60 [51]	1.70 (CCF)
Morbilisin E (**19**)	2.3 [39]	3.0 (OA)
Mongolicin C (**24**)	14.65 [46]	9.47 (UA)
	1.63 [14]	3.31 (OA)
Macrourin G (**26**)	2.52; *K*_i_ = 1.09 μM; Noncompetitive [46]	9.47 (UA)
Morusalbin A (**27**)	6.07 [46]	9.47 (UA)
Mulberrofuran G (**31**)	0.57; *K*_i_ = 0.70 μM; Mixed-competitive [168]	3.54 (UA)
	20.03 [61]	13.27 (RK)
	4.56 [46]	9.47 (UA)
	6.99 [14]	3.31 (OA)
Mulberrofuran F (**32**)	0.57 [14]	3.31 (OA)
Mulberrofuran K (**40**)	8.49 [46]	9.47 (UA)
	1.62 [14]	3.31 (OA)
Morbilisin A (**41**)	1.9 [39]	3.0 (OA)
Morusalbin B (**42**)	6.77 [46]	9.47 (UA)
Yunanensin A (**44**)	5.26 [46]	9.47 (UA)
Albanol B (**46**)	2.31 [46]	9.47 (UA)
	0.80; *K*_i_ = 1.02 μM; Mixed-competitive [168]	3.54 (UA)
Morusalbin C (**49**)	9.67 [46]	9.47 (UA)
Morusalisin F (**53**)	1.14 [51]	1.70 (CCF)
Kuwanol A (**64**)	1.58 [14]	3.31 (OA)
Morusalbin D (**67**)	1.90; *K*_i_ = 0.33 μM; Noncompetitive [46]	9.47 (UA)
Kuwanon J (**81**)	7.49 [14]	3.31 (OA)
	2.7; *K*_m_ = 2.81 ± 0.08 mM; Mixed-competitive [98]	3.8 (UA); 4.7 (RK)
Kuwanon R (**86**)	1.57 [14]	3.31 (OA)
	8.2; *K*_m_ = 1.64 mM; Mixed-competitive [98]	3.8 (UA); 4.7 (RK)
Kuwanon V (**87**)	13.8; *K*_m_ = 1.19 mM; Mixed-competitive [98]	3.8 (UA); 4.7 (RK)
Kuwanon G (**92**)	13.07 [61]	13.27 (RK)
	2.26; *K*_i_ = 1.98 μM; Mixed-competitive [168]	3.54 (UA)
Kuwanon H (**93**)	4.04 [61]	13.27 (RK)
Kuwanon L (**118**)	16.9 [43]	4.5 (RK)
	21.67 [61]	13.27 (RK)
Sanggenon G (**124**)	1.6 [43]	4.5 (RK)
	10.87 [61]	13.27 (RK)
Sanggenon C (**130**)	2.6 [43]	4.5 (RK)
Kuwanon M (**151**)	10.71 [61]	13.27 (RK)
( +)-Morusalone A (**154**)	0.72 [137]	1.70 (CCF)
( +)-Morusalone B (**155**)	1.03 [137]	1.70 (CCF)
( −)-Morusalone A (**156**)	1.01 [137]	1.70 (CCF)
( −)-Morusalone B (**157**)	1.02 [137]	1.70 (CCF)
Morusalone C (**158**)	0.35 [137]	1.70 (CCF)
Morusalone D (**159**)	1.99 [137]	1.70 (CCF)
Morusalisin A (**160**)	1.55 [51]	1.70 (CCF)
Morusalisin B (**161**)	2.24 [51]	1.70 (CCF)
Mulberrofuran H (**162**)	78.96 [46]	9.47 (UA)

^a^*OA* Oleanolic acid, *CCF* CCF06240, *RK* RK-682, *UA* Ursolic acid

### *α*-Glucosidase inhibitory activity

*α*-Glucosidase is a key enzyme involved in carbohydrate digestion, and its inhibitors can be effective drugs in the management of postprandial blood glucose and insulin levels in type 2 diabetic patients [169]. Ha et al. found that all the PTP1B inhibitors albasin B (**8**), macrourin G (**26**), morusalbin A (**27**), mulberrofuran G (**31**), mulberrofuran K (**40**), morusalbin B (**42**), yunanensin A (**44**), albanol B (**46**), morusalbin C (**49**), and morusalbin D (**67**) exhibited strong inhibitory activities against *α*-glucosidase with IC_50_ ranging from 2.29 to 5.91 μM. The type of *α*-glucosidase inhibition of the active MDAAs **8**, **26**, **44**, and **67** with *K*_i_ values of 0.42, 2.42, 1.19, and 0.64 μM, respectively, were determined to be competitive-type by enzyme kinetic studies [46]. In addition to these ten *α*-glucosidase inhibitors, mongolicin C (**24**) and mulberrofuran H (**162**) also had significant inhibitory effects on *α*-glucosidase [46]. In 2018, two additional MDAAs sanggenon G (**124**, IC_50_ = 11.96 μM) and sanggenon O (**131**, IC_50_ = 3.06 μM) with significant inhibitory activity against *α*-glucosidase were identified from crude extracts of Sang-Bai-Pi by a novel screening strategy based on the ligand fishing combined with HPLC-QTOF-MS and molecular docking [69]. The other MDAAs with potent inhibitory effects on *α*-glucosidase were listed in Table 7 [32, 48, 61, 68, 97, 168].

**Table 7:** Inhibitory activities of MDAAs against *α*-glucosidase

Compd	Activity (IC_50_ in μM); *K*_i_ value; Inhibition type	Positive control (acarbose, IC_50_ in μM)^a^
Chalcomoracin (**5**)	6.00 [32]	0.02
Albasin B (**8**)	2.90; *K*_i_ = 0.42 μM; Competitive [46]	203.97
Mulberrofuran E (**9**)	5.22 [48]	1428
Wittiorumin F (**18**)	1.89 [48]	1428
Macrourin I (**21**)	1.70 [48]	1428
Macrourin J (**22**)	1.58 [48]	1428
Mongolicin C (**24**)	4.79 [46]	203.97
Macrourin G (**26**)	3.61; *K*_i_ = 2.42 μM; Competitive [46]	203.97
Morusalbin A (**27**)	4.53 [46]	203.97
Mulberrofuran G (**31**)	2.29 [46]	203.97
	1.07 [61]	293.50
	2.48 [68]	85.29 (1-DNJ)
	1.67; *K*_i_ = 1.2 μM; Mixed-competitive [168]	119.16
Mulberrofuran F (**32**)	2.13 [48]	1428
Mulberrofuran K (**40**)	5.91 [46]	203.97
	1.25 [48]	1428
Morusalbin B (**42**)	5.07 [46]	203.97
Yunanensin A (**44**)	3.64; *K*_i_ = 1.19 μM; Competitive [46]	203.97
Albanol B (**46**)	4.34 [46]	203.97
	1.31; *K*_i_ = 0.9; Mixed-competitive [168]	119.16
Morusalbin C (**49**)	5.40 [46]	203.97
Albafuran C (**50**)	2.52 [48]	1428
Sorocein I (**65**)	1.47 [48]	1428
Morusalbin D (**67**)	3.55; *K*_i_ = 0.64 μM; Competitive [46]	203.97
Kuwanon J (**81**)	30.3 [97]	332
	2.17 [48]	1428
Kuwanon R (**86**)	48.3 [97]	332
Kuwanon G (**92**)	2.35; *K*_i_ = 2.51; Mixed-competitive [168]	119.16
	2.80 [61]	293.50
Kuwanon H (**93**)	0.82 [61]	293.50
Deoxyartonin I (**102**)	7.80 [97]	332
Kuwanon L (**118**)	11.03 [61]	293.50
Sanggenon G (**124**)	2.97 [61]	293.50
	11.96 [69]	55.40 (LUT)
Sanggenon O (**131**)	3.06 [69]	55.40 (LUT)
Kuwanon M (**151**)	0.60 [61]	293.50
Mulberrofuran H (**162**)	12.51 [46]	203.97

^a^*1-DNJ* 1-Deoxynojirimycin, *LUT* Lutenolin

### Tyrosinase inhibitory activity

Tyrosinase is a rate-limiting enzyme in the formation of melanin pigments in mammals and the key enzyme for enzymatic browning of many plant-derived food products [170]. Therefore, tyrosinase inhibitors are crucial in the pharmaceutical, skin whitening cosmetic, and food industries. In 2004, Lee et al*.* first discovered that the natural MDAA sanggenon D (**128**, IC_50_ = 7.3 μM) was a potent tyrosinase inhibitor (the positive control kojic acid, IC_50_ = 24.8 μM) [171]. Subsequently, more than 20 MDAAs were reported to have significant inhibitory activity against tyrosinase (Table 8) [19, 21, 32, 36, 70, 172, 173].

**Table 8:** Inhibitory activities of MDAAs against tyrosinase

Compd.	Activity (IC_50_ in μM)	Positive control (kojic acid, IC_50_ in μM)
Mulberrofuran J (**1**)	191.28 [21]; 4.54 [19]	46.95; 19.60
Mongolicin F (**2**)	3.20 [19]	19.60
Chalcomoracin (**5**)	2.59 [36]; 2.27 [19]; 5.61 [32]	32.62; 19.60; 14.18 (Arbutin)
Mulberrofuran O (**13**)	1.83 [19]	19.60
Mulberrofuran G (**31)**	6.35 [173]; 17.53 [21]; 14.65 [70]	36.0; 46.95; 50.43
Albanol B (**46**)	> 350 [173]	36.0
Guangsangon J (**51**)	0.67 [19]	19.60
Guangsangon E (**56**)	0.57 [19]	19.60
Macrourin F (**59**)	0.50 [19]	19.60
Macrourin E (**60**)	0.39 [19]	19.60
Kuwanol E (**62**)	0.54 [19]	19.60
Sorocein A (**68**)	0.67 [19]	19.60
Kuwanon J (**81**)	0.17 [36]	32.62
Kuwanon G (**92**)	67.6 [173]; > 200 [21]; > 200 [70]	36.0; 46.95; 50.43
Kuwanon H (**93**)	10.34 [21]	46.95
Moracenin D (**95**)	4.61 [70]	50.43
Kuwanon N (**110**)	78.95 [70]	50.43
Kuwanon L (**118**)	58.82 [70]	50.43
Kuwanon O (**119**)	1.81 [70]	50.43
Sanggenon T (**124**)	1.20 [70]	50.43
Sanggenon D (**128**)	7.3 [171]	24.8
Sanggenon C (**130**)	1.17 [36]	32.62
Sanggenon O (**131**)	1.15 [36]	32.62
Sorocein H (**132**)	6.49 [36]	32.62

### Antiviral activity

According to the anti-HBV assay on the HepG 2.2.15 cell line in vitro, mulberrofuran G (**31**) exhibited moderate inhibitory activity against hepatitis B virus (HBV) DNA replication (IC_50_ = 3.99 μM) [66]. At non-toxic concentrations, kuwanon X (**58**) possessed prominent activities against herpes simplex virus type 1 and 2 (HSV-1 and HSV-2). The IC_50_ values of **58** to the tested strains HSV-1 (15,577), HSV-1 (clinical), and HSV-2 (333) were 2.2, 1.5 and 2.5 μg/mL, respectively. Mechanism studies revealed that **58** could inhibit HSV-1 adsorption and penetration, HSV-1 IE and L genes expression, viral DNA biosynthesis, and the HSV-induced nuclear factor (NF)-κB activation [174]. In the antiviral investigation of *Morus* spp. plant extracts, the antiviral activity against human coronavirus (HCoV 229E) of their common component kuwanon G (**92**) was also evaluated [175]. Kuwanon L (**118**) was found to have the inhibition of HIV-1 integrase (IN) catalytic activity in the absence and in the presence of LEDGF/p75 protein, and could inhibit the IN dimerization, the IN/LEDGF binding, as well as HIV-1 replication [176]. Further study suggested that kuwanon L (**118**) might exhibit its antiviral activity via binding to multiple viral targets, which may be a promising natural HIV-1 IN inhibitor [177]. Sanggenon G (**124**) had a certain inhibitory effect on influenza A virus (IC_50_ = 30.9 μM) [160].

### Anti-cardiovascular diseases

Nomura et al. found some MDAAs such as mulberrofuran C (**6**), mulberrofuran G (**31**), mulberrofuran F (**32**), kuwanon G (**92**), kuwanon H (**93**), sanggenon D (**128**), and sanggenon C (**130**) had clear hypotensive effects [1, 2, 5, 33, 124, 129]. In an in vitro evaluation carried out by Nikaido et al., these compounds and several other MDAAs showed strong inhibitory activities against beef heart cAMP phosphodiesterase with IC_50_ values ranging 1.0 − 64.0 μM. Nikaido et al. proposed that the possible correlation between the mode of inhibition activity of these compounds against cAMP phosphodiesterase and their hypotensive effects deserves further study [178]. Liu et al. found that kuwanon G (**92**) could attenuate atherosclerosis by upregulation of LXR*α*-ABCA1/ABCG1 and inhibition of NF-*κ*B activity in macrophages [179]. Gu et al. demonstrated that sanggenon C (**130**) exerted direct cytoprotective effects against hypoxia injury in cardiac cells via signaling mechanisms involving the activation of AMPK and concomitant inhibition of target of rapamycin (mTOR) and forkhead box O3a (FOXO3a) [180]. In the same year, Xiao et al. found that sanggenon C (**130**) could protect against cardiac hypertrophy and fibrosis via suppression of the calcineurin/NFAT2 pathway [181].

### Other activities

Some MDAAs have also been found as potential inhibitors of disease-related enzymes, such as phosphodiesterase 1 (PDE1) inhibitors [chalcomoracin (**5**), mesozygin B (**100**), artonin I (**101**), mesozygin C (**103**), and mesozygin A (**104**)] [35], human carboxylesterase 2 (*h*CE2) inhibitors [kuwanon G (**92**), sanggenon D (**128**), and sanggenon C (**130**)] [182], pancreatic lipase inhibitors [kuwanon G (**92**) and sanggenon D (**128**)] [183], *β*-glucuronidase inhibitors [kuwanon G (**92**) and sanggenon C (**130**)] [184, 185], and 5*α*-reductase inhibitors [palodesangrens C − E (**140** − **142**)] [132]. Mulberrofuran C (**6**) and mulberrofuran G (**31**) exhibited potent hepatoprotective activity on *t*-BHP-induced oxidative stress in HepG2 cells, with EC_50_ values of 0.41 and 15.31 μM, respectively [44]. Sanggenons C and D (**130** and **128**) were identified as positive *γ*-aminobutyric acid type A (GABA_A_) receptor modulators [186].

## Chemical and chemoenzymatic total syntheses of MDAAs

MDAAs exhibit kinds of structurally unique frameworks and a variety of promising bioactivities, and so these natural products have attracted extensive attention from synthetic chemists. Since the first report of total syntheses of several dehydroprenyl-2-arylbenzofuran type (Type A) MDAAs in 2010 [187], an amount of total syntheses of these molecules (including Types A − D and F − G) were disclosed over the past decade. Among all the synthetic strategies for MDAAs, the biomimetic intermolecular [4 + 2]-cycloaddition between a diene and a dienophile is the key step, which might be driven by Lewis acids, organocatalysts, and even enzymes. To the best of our knowledge, there have been no reviews focusing on the total synthesis of these MDAAs until now. In this section, we will cover all chemical and chemoenzymatic total syntheses of the MDAAs and their methyl ether derivatives.

### Chemical total syntheses of dehydroprenyl-2-arylbenzofuran type MDAAs (Type A)

#### Total syntheses of ( ±)-mulberrofuran J hexamethyl ether, ( ±)-mongolicin F hexamethyl ether, ( ±)-chalcomoracin heptamethyl ether, and ( ±)-mulberrofuran C heptamethyl ether

In 2010, the Rizzacasa group reported firstly the racemic total syntheses of four methyl ether derivatives of dehydroprenyl-2-arylbenzofuran type MDAAs including mulberrofuran J hexamethyl ether (**1a**), mongolicin F hexamethyl ether (**2a**), chalcomoracin heptamethyl ether (**5a**), and mulberrofuran C heptamethyl ether (**6a**), in which the unit of dehydroprenyl diene was proved to be a challenging intermediate due to its unstable. This diene unit was established by using a Suzuki–Miyaura coupling as the key step, and it was used immediately after rapid purification. They also proved that the presence of the H-bonded phenol in the chalcone dienophile was essential for the success of the [4 + 2]-cycloaddition [187].

**Scheme 2:**
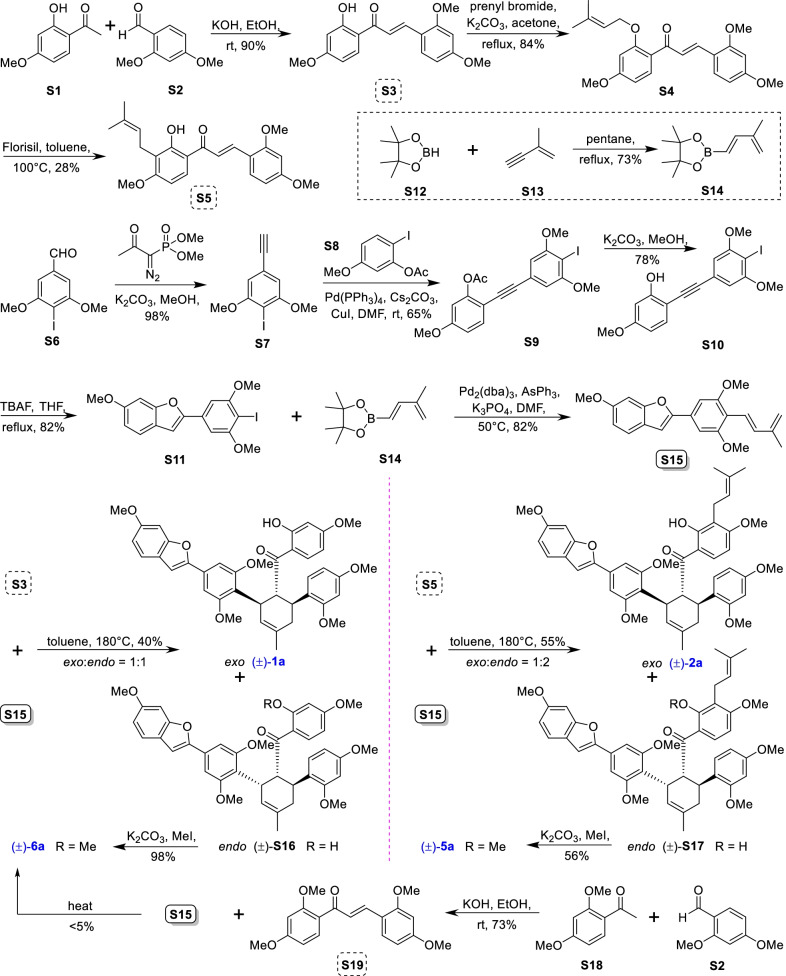
Syntheses of mulberrofuran J hexamethyl ether (**1a**), mongolicin F hexamethyl ether (**2a**), chalcomoracin heptamethyl ether (**5a**), and mulberrofuran C heptamethyl ether (**6a**) by the Rizzacasa group

As outlined in Scheme 2, the authors started the preparation of the chalcone dienophile **S3** from ketone **S1** and aldehyde **S2** via Claisen-Schmidt condensation. As for the synthesis of dienophile **S5**, chalcone **S3** was prenylated under standard conditions and the resultant prenyl ether **S4** was subjected to a Florisil promoted [1, 3]-sigmatropic rearrangement to afford the prenylated chalcone **S5** in 28% yield. Except for the [1, 3]-rearranged product, the corresponding [1, 5]-rearranged isomer (not shown) as well as **S3** were also produced in a significant amount.

With the use of Cs_2_CO_3_ rather than amine bases, Sonogashira coupling of **S8** and phenylalkyne **S7**, prepared by Seyferth-Gilbert homologation of 4-iodo-3,5-dimethoxybenzaldehyde **S6** with Bestman-Ohira reagent, successfully gave the bisphenylalkyne **S9** in good yield. After methanolysis of the acetate alkyne **S9**, the resulting phenol **S10** was subsequently subjected to cyclization into the benzofuran **S11** in 82% yield using TBAF instead of gold or platinum catalysis. The challenging formation of the dehydroprenyl-2-arylbenzofuran diene **S15** was achieved in excellent yield via the Suzuki–Miyaura coupling of iodide **S11** and pinacolboronate **S14** (preparation by simple hydroboration of enyne **S13** with pinacolborane **S12**) after extensive experimentation.

The intermolecular Diels–Alder cycloaddition reaction between dienophile **S3** and diene **S15** succeeded to proceed at 180 °C in toluene in a sealed tube to give the *exo* and *endo* adducts, *trans*,*trans-***1a** [the hexamethyl ether derivative of mulberrofuran J (**1**)] and *cis*,*trans-***S16**, respectively, in a 1:1 ratio. Cycloaddition of **S5** and **S15** under the same conditions afforded a 1:2 ratio of the *exo* adduct mongolicin F hexamethyl ether (**2a**) and the *endo* adduct **S17**. Subsequently, methylation of **S16** and **S17** obtained the previously reported mulberrofuran C heptamethyl ether (**6a**) and the permethylation product of chalcomoracin (**5**), chalcomoracin heptamethyl ether (**5a**), respectively, which assisted to confirm the stereochemistry of the *exo* and *endo* adducts. Unfortunately, the authors did not obtain the natural products mulberrofuran J (**1**), mongolicin F (**2**), chalcomoracin (**5**), and mulberrofuran C (**6**) by deprotection of either their hexamethyl ether derivatives or heptamethyl ether ones.

Claisen-Schmidt condensation of aldehyde **S2** and ketone **S18** instead of **S1** give the fully methylated dienophile **S19**, which failed to undergo clean cycloaddition with **S15**. This demonstrated that a H-bonded *ortho* OH substituent on the chalcone was critical for the success of the [4 + 2]-cycloaddition. Subsequent detailed studies including a computational investigation showed the acceleration of the cycloaddition reaction by the OH group arises both from the LUMO-lowering effect of the OH–carbonyl hydrogen bond and from better coplanarity between the diene and its aryl substituent in the transition structures [188]. Based on the Rizzacasa group's research, the subsequent synthesis of all chalcone dienophiles retained the presence of the free phenol in its C-2 position.

### Chemical total syntheses of dehydroprenylstilbene type MDAAs (Type B)

#### Enantioselective total syntheses of kuwanon X, kuwanon Y, and kuwanol A

In 2014, the Lei group developed a new strategy to forge the desired cyclohexene core unit of dehydroprenylchalcone type MDAAs. This new strategy featured an asymmetric Diels−Alder cycloaddition, catalyzed by a chiral ligand/boron Lewis acid to construct the core structure (see Sect. 4.3.4). In 2016, the same group adopted this strategy to construct the cyclohexene moiety of dehydroprenylstilbene type MDAAs. The biosynthesis-inspired asymmetric Diels − Alder cycloaddition shows high *exo* selectivity, in which the ratio of *exo*/*endo* could up to 13:1. The implementation of this strategy allowed for the first asymmetric total syntheses of the natural products kuwanons X and Y (**58** and **61**) and kuwanol A (**64**) in 12 and 11 steps, respectively [189].

**Scheme 3:**
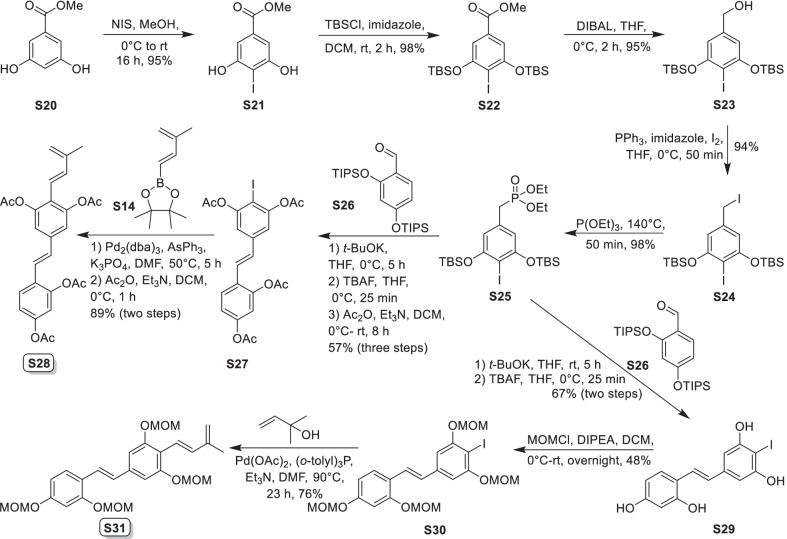
Syntheses of dienes **S28** and **S31** by the Lei group

The synthesis of dienes **S28** (acetyl-protection) and **S31** (MOM-protection) started with the preparation of **S25** from methyl 3,5-dihydroxy-benzoate **S20** in high yield over five steps including aromatic C-H iodination, TBS protection, DIBAL reduction, alkane C–OH iodination, and Michaelis-Arbuzov reaction (Scheme 3). The acetyl protected diene **S28** was obtained over four steps by the Horner-Wadsworth-Emmons reaction of **S25** with **S26** followed by deprotection of the silyl protecting groups and reprotection with acetyl groups, and then via the Suzuki–Miyaura reaction. The authors found that an additional step of acetyl reprotection of the crude mixture produced by the Suzuki reaction could improve the yield of diene **S28**. In a branch procedure, precursors **S25** and **S26** were subjected to the same Horner-Wadsworth-Emmons reaction and deprotection conditions, and then reprotected with MOM groups to produce iodide **S30**, which performed the Heck reaction with 2-methyl-but-3-en-2-ol followed by dehydration to provide the desired MOM protected diene **S31**.

**Scheme 4:**
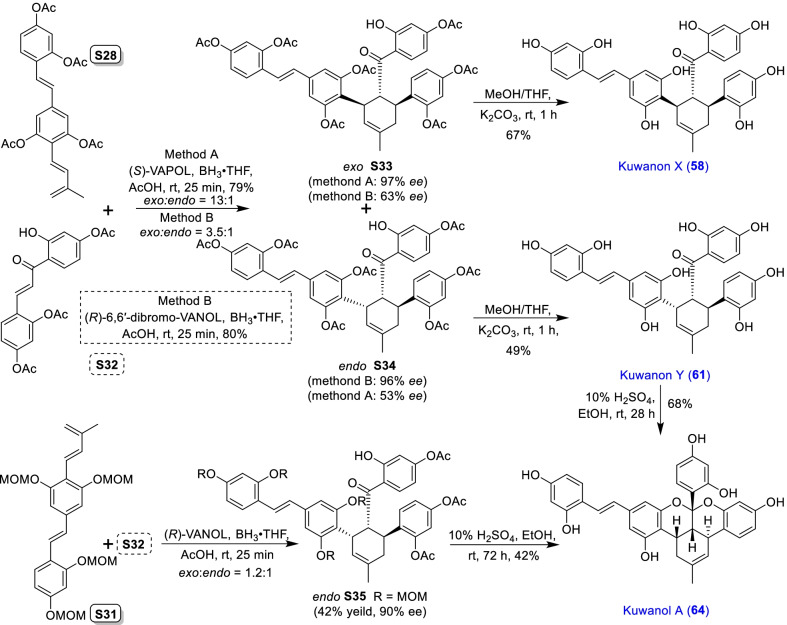
Syntheses of kuwanons X and Y (**58** and **61**) and kuwanol A (**64**) by the Lei group

The critical factor for the symmetric Diels − Alder cycloaddition between diene **S28** and the known dienophile **S32** was to find a suitable chiral ligand. After screening of chiral boron ligands for catalytic [4 + 2] cycloaddition, two chiral ligands (*S*)-VAPOL and (*R*)-6,6′-dibromo-VANOL could be used directly for the enantioselective total synthesis of acetyl ether precursors of **58** and **61**, respectively, due to their very high *exo*- or *endo*-selectivity and *ee* value. As shown in Scheme 4, (*S*)-VAPOL effectively catalyzed the cycloaddition with high *exo* selectivity (*exo*/*endo* = 13:1) and *ee* value (97%), while the chiral ligand (*R*)-6,6′-dibromo-VANOL gave a good *endo*-selectivity (*exo*/*endo* = 3.5:1) and satisfying *ee* value (96%). Deacetylation of the corresponding *exo*-**S33** and *endo*-**S34** in the presence of K_2_CO_3_ in the mixture of THF and MeOH furnished the natural products kuwanons X and Y (**58** and **61**), respectively. The biomimetic intramolecular ketalization of **58** and **61** was performed under the catalysis of sulfuric acid, but only **61** formed a ketalized product kuwanol A (**64**).

The Lei group had further investigated whether the two acetyl groups near the dehydroprenyl group had obvious effect on the *exo*/*endo* stereoselectivity. As shown in Scheme 4, by using the MOM-protected diene **S31** instead of the acetyl-protected diene **S28** in the synthesis of kuwanol A (**64**) from the same dienophile **S32** catalyzed by chiral (*R*)-VANOL-boron Lewis acid, the *exo*/*endo* stereoselectivity was changed from 5.3:1 to 1.2:1 without losing the enantioselectivity, but the total yield increased from 3.6% to 17.6% with one step shorter. Combined with other cases, the authors suggested that different substitutions in diene may contribute to different stereoselectivity in the asymmetric Diels − Alder cycloaddition.

#### Total syntheses of ( ±)-kuwanol E and ( ±)-kuwanon Y heptamethyl ether

Also in 2016, Iovine and coworkers completed their total syntheses of ( ±)-kuwanol E (**62**) and ( ±)-kuwanon Y heptamethyl ether (**61a**) via a convergent strategy in nine steps. The synthesis featured a Lewis acid-mediated biomimetic intermolecular Diels−Alder cycloaddition for creating a cyclohexene core unit from dienophiles **S3** or **S5** and dehydroprenylstilbene diene **S46**. Another key point in this synthesis was that its *exo*/*endo* diastereoselectivity was controlled by the reaction temperature [190].

**Scheme 5:**
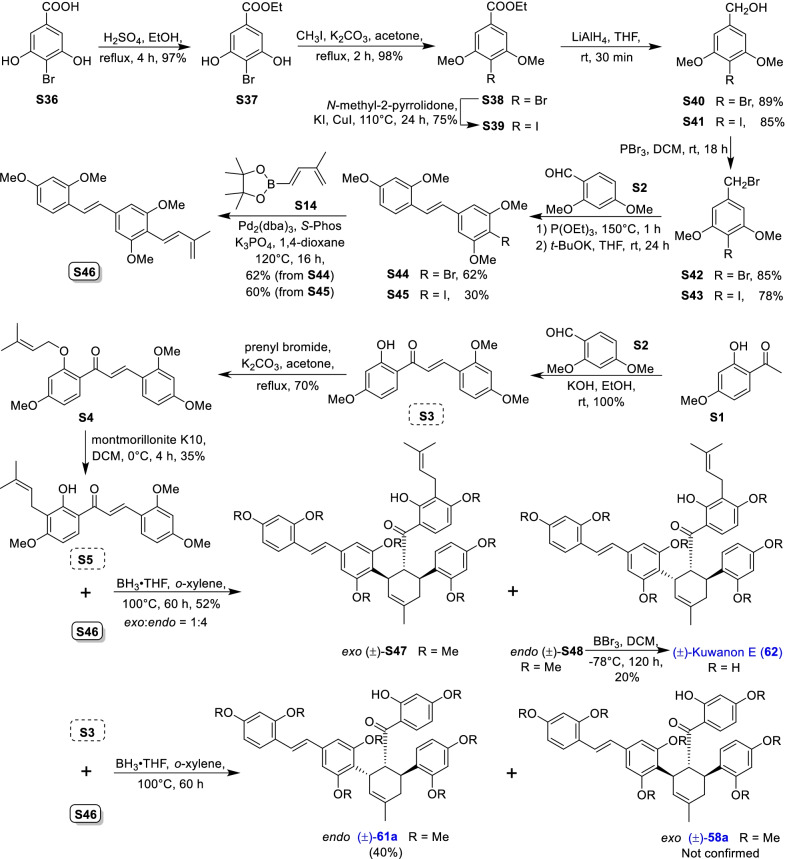
Syntheses of ( ±)-kuwanol E (**62**) and ( ±)-kuwanon Y heptamethyl ether (**61a**) by Iovine and coworkers

The synthesis commenced with the preparation of dienophile **S5** over several classical reported reactions involving Claisen-Schmidt condensation, prenylation, and sigmatropic rearrangement (Scheme 5). Different from the previous report, the use of montmorillonite K10 as the catalyst in [1, 3]-rearrangement could improve the yield of **S5**. As depicted in Scheme 5, the synthetic route towards the key intermediate **S46** started with commercially available 4-bromo-3,5-dihydroxybenzoic acid **S36**, which proceeded via Fischer esterification and methyl protection to obtain bromide **S38**. For exploring the effects of different halogen atoms (Br or I) on the subsequent reactions, iodide **S39** was obtained by aromatic Finkelstein iodination of bromide **S38**. Next, compounds **S38** or **S39** were reduced with LiAlH_4_, and then converted into benzyl bromides **S42** or **S43** using PBr_3_, which exhibited different yields over the two steps. The one-pot Arbuzov and Horner − Wadsworth − Emmons reactions of **S42** or **S43** with **S2** were performed to install the stilbene halides **S44** or **S45**, in which their yields differed by more than one time. After Suzuki − Miyaura coupling of **S44** or **S45** with boronate **S14**, diene **S46** was obtained in equivalent yield from the two building blocks, respectively. Therefore, the use of bromine substituted substrates in these reactions would give a better combined yield of diene **S46**. In addition, compared with traditional ligands such as PPh_3_ or AsPh_3_, the use of S-Phos as a bulky and electron-rich ligand in this Suzuki−Miyaura coupling step was proved to be a key condition affecting the yield of the product.

The Diels − Alder cycloaddition of dienophile **S5** and diene **S46** in dry *o*-xylene with or without the catalyst BH_3_·THF at various temperatures was performed, which exhibited different reactivity and diastereoselectivity. For example, at low temperatures (25 and 50 °C), the cycloaddition reaction had no reactivity, but as the temperature increased to 100 °C, a mixture of the *exo*- and *endo*-adducts (**S47** and **S48**) with a 1:4 ratio was obtained. However, when the temperature was further heated to 160 °C, it yielded **S47** and **S48** in the opposite ratio (4:1). Therefore, the production of the *endo*-isomers such as ( ±)-kuwanol E heptamethyl ether (**S48**) and ( ±)-kuwanon Y heptamethyl ether (**61a**) was carried out under the same conditions as shown in Scheme 5. Subsequently, ( ±)-kuwanol E (**62**) was obtained by demethylation of the corresponding *endo*-isomer **S48** with BBr_3_ in DCM. In this study, the cleavage of the methoxy groups of the heptamethyl ether precursor of ( ±)-kuwanon Y was not attempted due to its unavailable as well as because of the completed total synthesis of kuwanon Y by Lei group.

### Chemical total syntheses of dehydroprenylchalcone type MDAAs (Type C)

#### Total syntheses of ( ±)-dorsterone pentamethyl ether and ( ±)-kuwanon V pentamethyl ether

**Scheme 6:**
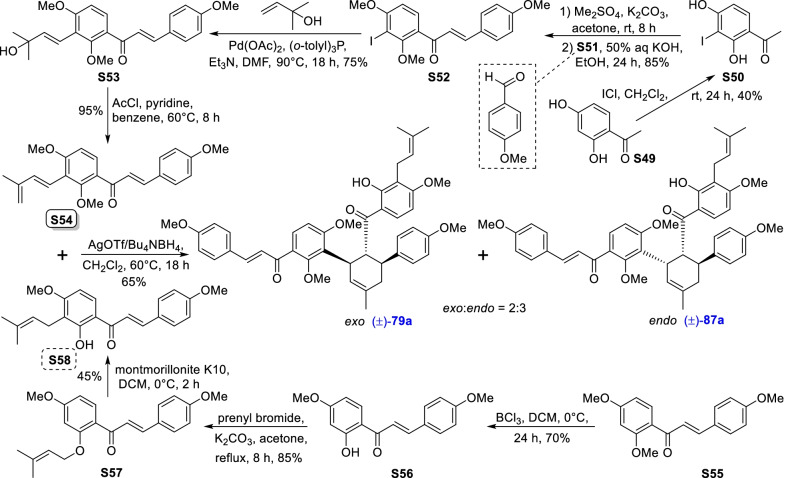
Syntheses of ( ±)-dorsterone pentamethyl ether (**79a**) and ( ±)-kuwanon V pentamethyl ether (**87a**) by the Rahman group

In 2011, the Rahman group reported the total synthesis of the pentamethyl ether derivatives **79a** and **87a** of dorsterone (**79**) and kuwanon V (**87**), respectively, via a Diels–Alder cycloaddition catalyzed by AgOTf/Bu_4_NBH_4_ [191]. The synthesis of diene **S54** started with commercially available 1-(2,4-dihydroxyphenyl)ethanone (**S49**) that underwent iodination, methylation, and Claisen–Schmidt condensation with 4-methoxybenzaldehyde (**S51**) in three steps to yield **S52** (Scheme 6). Next, Heck coupling of **S52** with 2-methylbut-3-en-2-ol occurred under the treatment of Pd(OAc)_2_, (*o*-tolyl)_3_-P, and Et_3_N in DMF and the resultant **S53** was dehydrated to afford diene **S54** in the presence of AcCl/pyridine. The preparation of dienophile **S58** was shown in Scheme 6. After the 2-methoxy group of the known chalcone **S55** was selectively demethylated, at the position of this OH group, a prenyl was introduced under standard conditions. Next, the resulting prenyl ether **S57** was subjected to a montmorillonite K10 promoted [1, 3]-rearrangement to afford the desired dienophile **S58** in 45% yield. After screening of the reaction conditions, the key AgOTf/Bu_4_NBH_4_-catalyzed [4 + 2] cycloaddition of chalcones **S54** and **S58** occurred smoothly to deliver the *exo*- and *endo*-adducts **79a** and **87a** in 65% yield with a diastereomeric ratio of 2:3.

#### Total syntheses of ( ±)-kuwanon I heptamethyl ether and ( ±)-kuwanon J heptamethyl ether

**Scheme 7:**
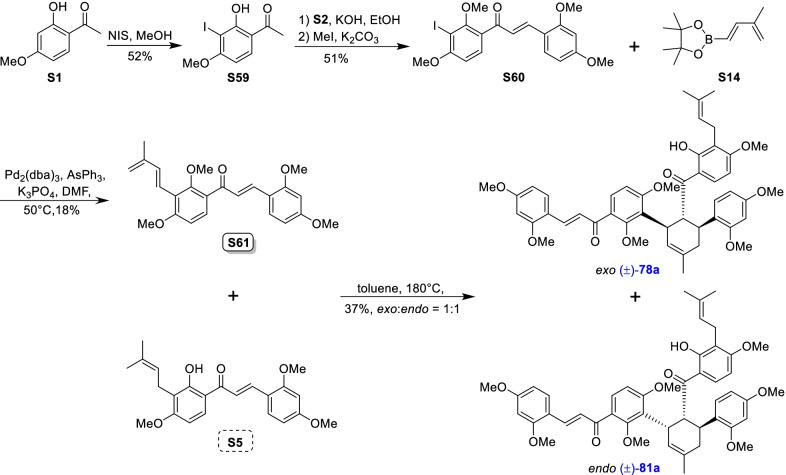
Syntheses of ( ±)**-**kuwanon I heptamethyl ether (**78a**) and ( ±)**-**kuwanon J heptamethyl ether (**81a**) by the Rizzacasa group

In the studies of H-bond accelerated Diels–Alder cycloadditions of chalcones, in 2012 the Rizzacasa group had also completed the total syntheses of two heptamethyl ethers of the dehydroprenylchalcone type MDAAs to examine the cycloaddition reaction [188]. As shown in Scheme 7, the synthesis of the diene **S61** started with the conversion of ketone **S1** to iodide **S59**, which was then subjected to Claisen–Schmidt condensation with aldehyde **S2** followed by methylation to afford a fully esterified chalcone **S60**. The subsequent Suzuki coupling with boronate **S14** gave diene **S61** in low yield, which underwent the [4 + 2]-cycloaddition reaction with the prepared dienophile **S5** only to afford kuwanon I heptamethyl ether (**78a**) and the *endo*-isomer kuwanon J heptamethyl ether (**81a**) in a 1:1 ratio without the product resulting from the Diels–Alder reaction of diene **S61** with itself as the dienophile. This result was also a proof to the importance of the H-bond in chalcone dienophile.

#### Asymmetric total syntheses of ( +)-brosimones A and B

In 2013, the Porco group described their asymmetric total syntheses of brosimone A (**80**) and brosimone B (**77**) through biomimetic dehydrogenative Diels–Alder cycloadditions. The key steps towards these target molecules involved Pt/C-cyclopentene or 2,3-dichloro-5,6-dicyano-1,4-benzoquinone (DDQ) to effect dehydrogenation of prenylchalcones in combination with silver nanoparticles (AgNPs) to catalyze subsequent Diels − Alder cycloaddition [192].

**Scheme 8:**
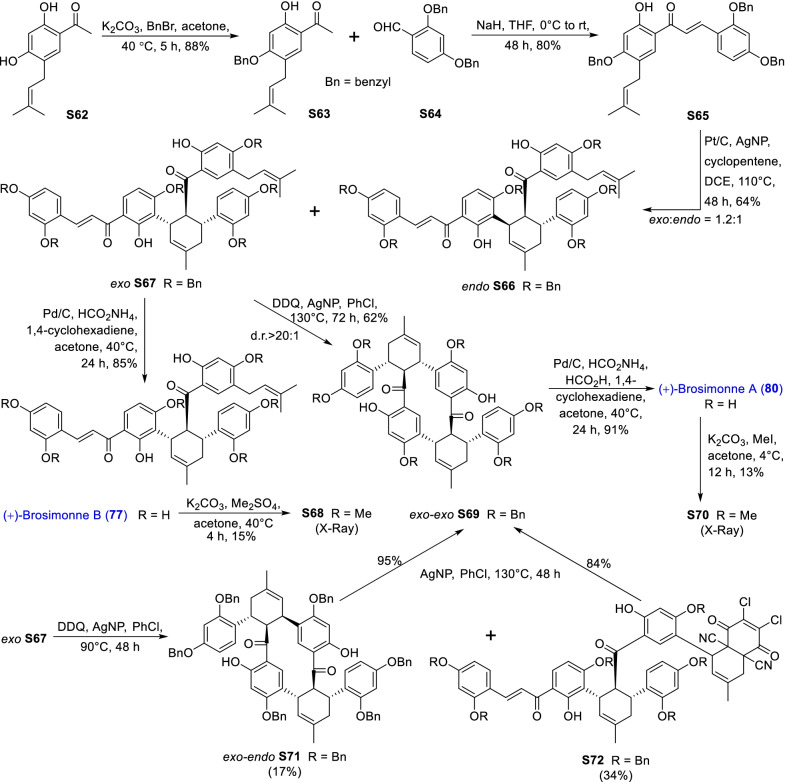
Asymmetric total syntheses of ( +)-brosimones A and B (**80** and **77**) by the Porco group

Brosimones A and B were homodimers derived from prenyl chalcone. Therefore, the synthesis started with the easily prepared acetophone **S62** that underwent a two-step procedure to yield the benzyl-protected prenyl chalcone **S65** (Scheme 8). Based on the model reaction established by the authors, the cycloadducts *exo*-**S67** and *endo*-**S66** with a ratio of 1.2:1 were obtained in 64% yield by dehydrogenative Diels–Alder cycloaddition/dimerization of **S65** using the optimized Pt/C-AgNP conditions with cyclopentene as H_2_ scavenger. Next, hydrogenolysis of *exo*-**S67** produced ( +)-brosimone B (**77**), whose structure was confirmed by X-ray crystal analysis of its methyl-protected derivative **S68** produced by methylation using Me_2_SO_4_. As shown in Scheme 8, the precursor *exo*-*exo* **S69** of brosimone A also could be accessed by dehydrogenative cycloaddition of *exo*-**S67** under different conditions including changing the temperature. For example, in the presence of DDQ and AgNPs as catalyst in chlorobenzene (PhCl) solvent, *exo*-**S67** was converted into the cycloadduct *exo*-*endo*-**S71** (17% yield) and the DDQ adduct **S72** (34% yield) at 90 °C. Both *exo*-*endo*-**S71** and DDQ adduct **S72** could be converted exclusively into *exo*-*exo* **S69** under AgNP-promoted conditions at 130 °C in excellent yield. When the reaction temperature was increased to 130 °C, the dehydrogenative Diels–Alder cycloaddition of *exo*-**S67** predominantly afforded *exo*-*exo* **S69** in 62% yield in the presence of AgNPs. Finally, the hydrogenolysis product of *exo*-*exo* **S69** was determined to be ( +)-brosimone A (**80**) on the basis of the X-ray structure of its methyl derivative **S70**.

#### Enantioselective biomimetic total syntheses of kuwanons I and J and brosimones A and B

In 2014, the Lei group reported the first enantioselective total syntheses of dehydroprenylchalcone type MDAAs kuwanon I (**78**), kuwanon J (**81**), brosimone A (**80**), and brosimone B (**77**) by a common intermediate based on a concise synthetic strategy. The key feature of the synthesis included a biosynthesis-inspired asymmetric Diels–Alder cycloaddition mediated by a chiral ligand/boron Lewis acid. Another important progress involved regioselective Schenck ene reaction, reduction, and dehydration to realize a biomimetic dehydrogenation for generation of the required diene precursor. Furthermore, a remarkable process involved a tandem inter-/intramolecular asymmetric Diels–Alder cycloaddition of a diene with itself as the dienophile [193].

**Scheme 9:**
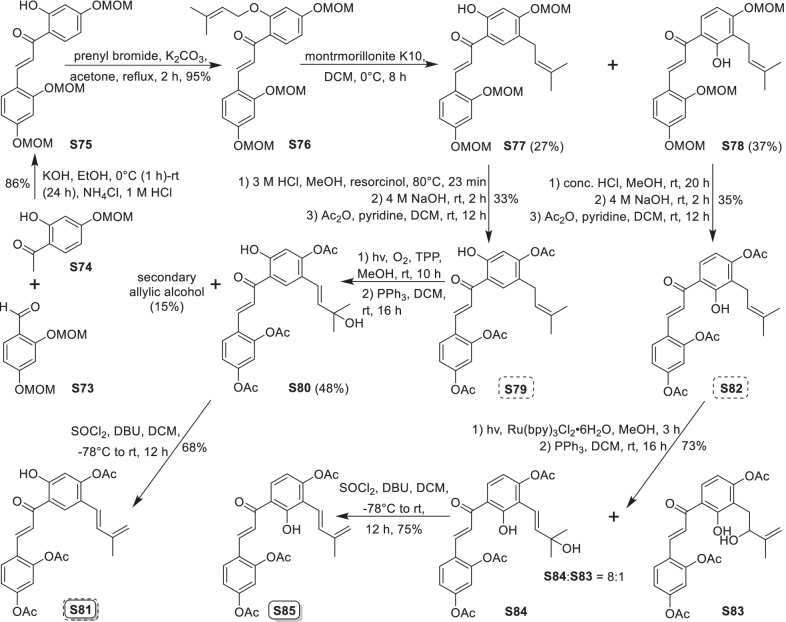
Syntheses of dienophiles (**S79**, **S81**, and **S82**) and dienes (**S81** and **S85**) by the Lei group

As shown in Scheme 9, base-mediated Claisen-Schmidt condensation of the MOM-protected aldehyde **S73** and ketone **S74** readily produced 2' hydroxychalcone **S75**. The subsequent prenylation and sigmatropic rearrangement resulted in the formation of two isomers, *para*- and *ortho*-prenylated chalcones (**S77** and **S78**). Next, the acetyl-protected dienophile **S79** was obtained from the *para*-prenylated chalcone **S77** in 33% yield by replacing MOM groups with acyl groups. After a brief optimization, the key visible-light-mediated regioselective Schenck ene reaction of **S79** by using TPP as photosensitizer and MeOH as solvent occurred smoothly to deliver the tertiary allylic alcohol **S80** and secondary allylic alcohol (not shown in Scheme 9) in 3.2:1 ratio. Then dehydration of **S80** with SOCl_2_/DBU produced the diene or dienophile **S81** in 68% yield. In a parallel procedure, deprotection of the MOM groups followed by reprotection of the *ortho-*isomer **S78** with acetyl groups delivered the required dienophile triacetate **S82** in 35% yield. Next, the visible-light-mediated regioselective Schenck ene reaction of **S82** using Ru(bpy)_3_Cl_2_·6H_2_O and MeOH gave an excellent ratio for the tertiary alcohol **S84**, a better substrate for dehydration, which smoothly provided the diene **S85** under SOCl_2_/DBU in 75% yield.

**Scheme 10:**
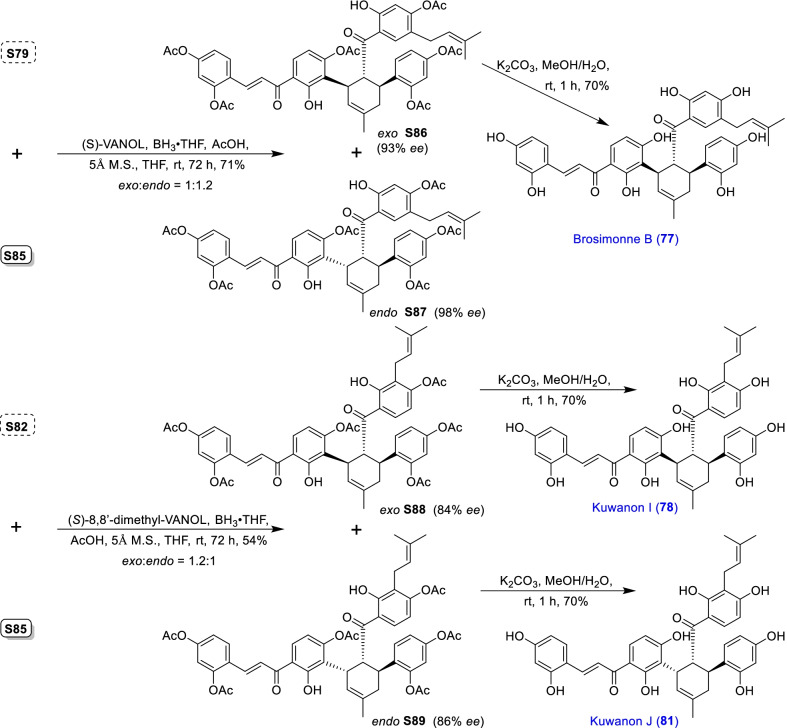
Enantioselective total syntheses of brosimone B (**77**), kuwanon I (**78**), and kuwanon J (**81**) by the Lei group

With dienophiles **S79**, **S81**, and **S82** and dienes **S81** and **S85** in hand, a series of asymmetric Diels–Alder reactions promoted by different ligand/boron Lewis acid for the synthesis of the target dehydroprenylchalcone type MDAAs were investigated. As depicted in Scheme 10, the preferred (*S*)-VANOL ligand catalyzed the cycloaddition of **S79** and **S85** to afford the *exo*- and *endo*-adducts (**S86** and **S87** with a 1:1.2 ratio) in 71% combined yield with excellent *ee* values for both. Similarly, the using of (*S*)-8,8’-dimethyl-VANOL was proved to be the best chiral ligand to obtain both *exo*-**S88** and *endo*-**S89** with good *ee* values. Final deprotection of the acetyl groups of *exo*-**S86**, *exo*-**S87**, and *endo*-**S89** with K_2_CO_3_ as a base efficiently furnished the desired natural products brosimone B (**77**), kuwanon I (**78**), and kuwanon J (**81**), respectively, each in 70% yield.

**Scheme 11:**
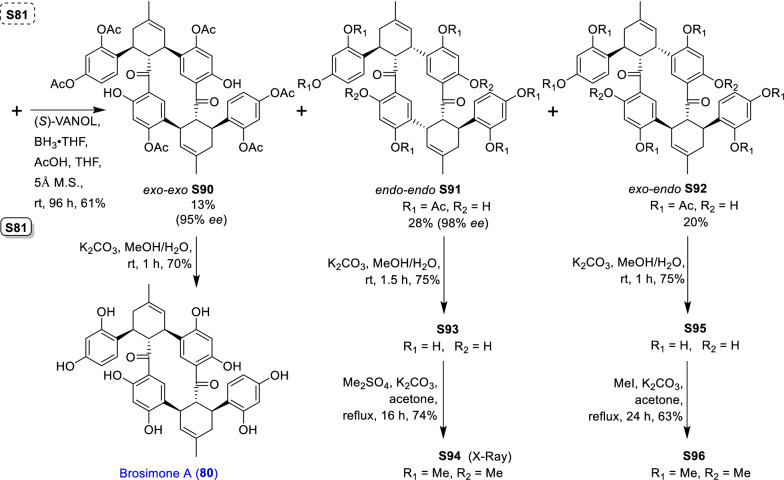
Enantioselective total synthesis of brosimone A (**80**) by the Lei group

It is worth mentioning that a one-pot inter-/intramolecular Diels–Alder cycloaddition cascade was smoothly occurred to afford the three expected products including *exo*,*exo*-**S90** in 13% yield, *endo*,*endo*-**S91** in 28%, and *exo*,*endo*-**S92** in 20% yield under the condition of a small amount of excess (*S*)-VANOL ligand (Scheme 11). Next, deprotection of *exo*,*exo*-**S90** under mild basic conditions efficiently gave the target natural product brosimone A (**80**) in 70% yield. In addition, after removing the acetyl groups of **S91** and **S92** followed by methylation, **S94** and **S96** with definite structures were obtained over two steps, respectively.

### Chemical total syntheses of dehydroprenylflavone type MDAAs (Type D)

#### Total syntheses of kuwanons G and H

In 2021, the Tang group reported a convergent route towards the total synthesis of two MDAAs named kuwanons G and H (**92** and **93**) with unique dehydroprenylflavone dienes. The key features of this approach included the use of Baker-Venkataraman rearrangement, alkylation of *β*-diketone, intramolecular cyclization, and Suzuki–Miyaura coupling to achieve the unstable dehydroprenylflavone diene [194].

**Scheme 12:**
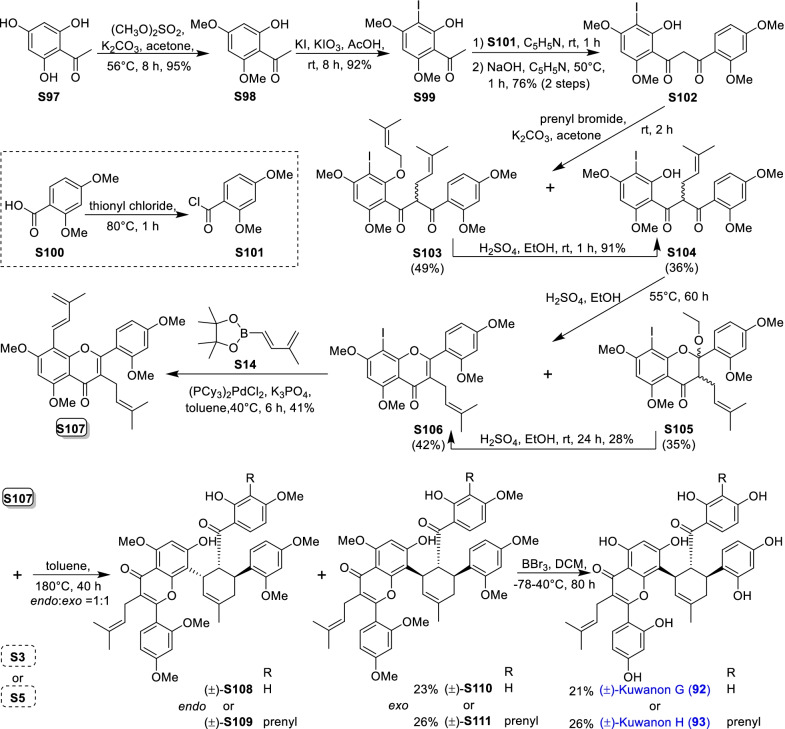
Syntheses of kuwanons G and H (**92** and **93**) by the Tang group

As outlined in Scheme 12, the synthesis of the key intermediate diene **S107** started with a selective methyl protection of 2′,4′,6′-trihydroxyacetophenone (**S97**). The addition of two methyls onto the acetophenone **S97** was performed using (CH_3_O)_2_SO_2_ in acetone to give **S98**. The following regioselective iodination of **S98** resulted in the formation of iodobenzene **S99** in 92% yield. After acylation of **S100** with thionyl chloride, the crude product benzoyl chloride **S101** was directly conducted with **S99** to form an acyloxy ketone, which was then converted into *β*-diketone **S102** through a base-catalyzed Baker–Venkataraman rearrangement. Next, alkylation of *β*-diketone **S102** with prenyl bromide gave the desired **S104** and by-product **S103**, and the later could be effectively hydrolyzed to the former. The treatment of **S104** with concentrated sulfuric acid in anhydrous ethanol enabled intramolecular cyclization onto the target cyclic product **S106** in 42% yield as well as an ethylated by-product **S105**, which also could be further converted to **S106** under acidic condition. With the aim of installing the diene moiety at C-8, the iodoflavonoid **S106** was subjected to the Suzuki–Miyaura coupling reaction with the easily prepared **S14** to dehydroprenylflavone diene **S107** in 41% yield.

The chalcone dienophiles **S3** and **S5** were easily furnished by the established route. As shown in Scheme 12, thermal-mediated intramolecular Diels–Alder cycloaddition of diene (**S107**) and dienophiles (**S3** and **S5**) in a sealed tube with toluene smoothly occurred to the *endo*- and *exo*-adducts with a ratio of 1:1. Deprotection of the corresponding *exo*-isomers ( ±)-kuwanon G heptamethyl ether (**S110**) and ( ±)-kuwanon H heptamethyl ether (**S111**) finally yielded ( ±)-kuwanons G (**92**) and H (**93**), respectively, which were separated by a chiral HPLC to obtain the two natural products (–)-kuwanon G and (–)-kuwanon H.

### Chemical total syntheses of dehydroprenylsanggenonflavone type MDAAs (Type F)

#### Asymmetric total syntheses of sanggenons C and O

In 2016, the Porco group completed the asymmetric total syntheses of sanggenons C and O (**130** and **131**). The syntheses relied on a Lewis acid-promoted double Claisen rearrangement of a bis-allyloxyflavone to install the hydrobenzofuro[3,2-*b*]chromenone core structure of sanggenonflavone diene precursors, and a stereodivergent reaction of a racemic mixture (stereodivergent RRM) involving the B(OPh)_3_/BINOL complexes catalytic enantioselective [4 + 2] cycloaddition to furnish the target molecules [195].

**Scheme 13:**
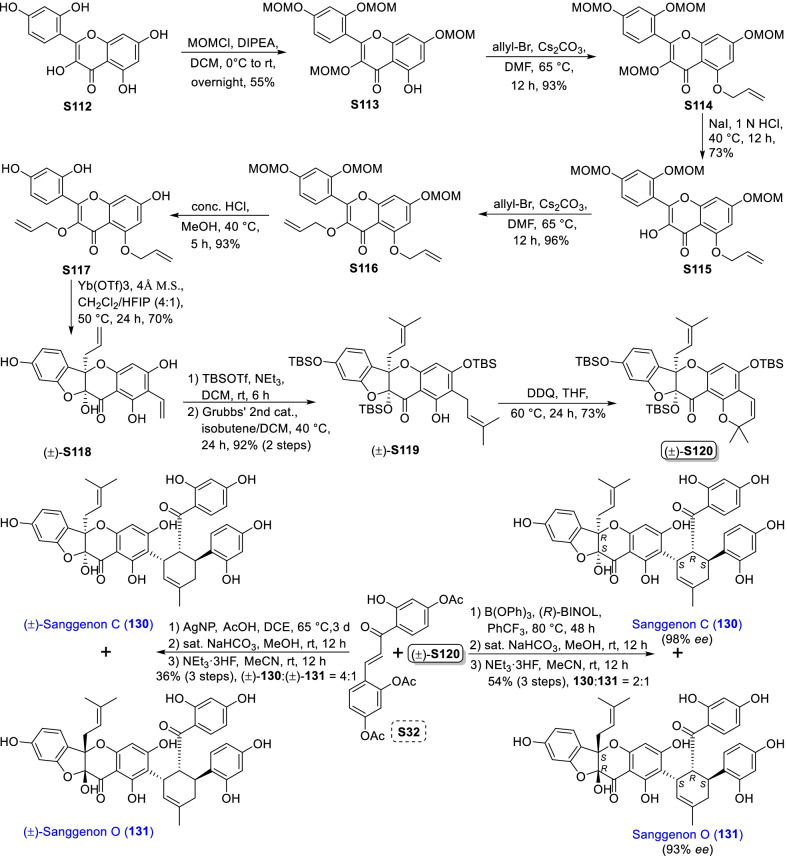
Asymmetric total syntheses of sanggenons C and O (**130** and **131**) by the Porco group

The synthesis of the diene precursor **S120** started with tetra-MOM group protection of the commercially available morin **S112** (Scheme 13). Subsequent 5-allylation of the MOM-protected flavonoid **S113** to afford the intermediate **S114**, which was transformed to **S115** by a chemoselective deprotection of the 3-MOM group using NaI and a catalytic amount of aqueous HCl. After allylation of the C-3 free hydroxyl group of **S115**, the obtained product **S116** was subjected to global deprotection to afford the desired bis-allyloxyflavone **S117**. After evaluation of a number of rare earth metal triflates for double rearrangement, it was found that Yb(OTf)_3_, in the presence of CH_2_Cl_2_/HFIP (4:1), was used for producing the desired hydrobenzofuro[3,2-*b*]chromenone core structure **S118** in 72% yield. With **S118** in hand, the authors carried out silylation and crossmetathesis to afford the tri-silyl-protected ( ±)-sanggenol F [( ±)-**S119**] in excellent yield. The prenyl product ( ±)-**S119** was then treated with 2,3-dichloro-5,6-dicyano-1,4-benzoquinone (DDQ) in tetrahydrofuran (THF) to transform to chromene ( ±)-**S120** in 73% yield.

The Porco group had applied two different strategies to construct the cyclohexene core unit. First, AgNPs-mediated intermolecular Diels–Alder cycloaddition of the TBS-protected diene precursor ( ±)-**S120** with acetylated 2′-hydroxychalcone **S32** smoothly occurred to yield a mixture of two *endo* cycloadducts and minimal production of *exo* diastereomers. The mixture of *endo* cycloadducts was sequentially treated with aqueous NaHCO_3_ and NEt_3_·3HF to yield a mixture of ( ±)-sanggenon C (**130**) and ( ±)-sanggenon O (**131**) in 36% combined yield over three steps. Next, in order to synthesize enantioenriched sanggenons C and O, a verified catalytic system to a stereodivergent RRM strategy was applied. Based on the model reaction, the asymmetric [4 + 2] cycloaddition of diene precursor ( ±)-**S120** with dienophile **S32** using B(OPh)_3_/(*R*)-BINOL as catalyst was carried out under the conditions of PhCF_3_. After sequential deprotection of both acetate and silyl protecting groups, the promising enantioselectivities sanggenon C (**130**, with 98% *ee*) and sanggenon O (**131**, with 93% *ee*) were obtained in 2:1 ratio.

### Chemical total syntheses of dehydroprenylcoumarin type MDAAs (Type G)

#### Total syntheses of palodesangren B trimethyl ether and palodesangren D dimethyl ether

In 2019, the Ploypradith group reported the diastereoconvergent total synthesis of the palodesangrens B and D methyl ethers (**139a** and **141a**), which were completed by installing the final 2*H*-pyran-2-one ring onto the tricyclic 9-methyl-6,7-diphenyl-6a,7,8,10a-tetrahydro-6*H*-benzo[*c*]chromene core constructed from appropriate chalcones and dienes. At the early stage of the synthetic route, the Diels–Alder reaction was utilized to assemble the cyclohexene moiety of the tricyclic core. Next, a novel diastereoconvergent LiAlH_4_-mediated isomerization of the mixture of *exo*- and *endo*-adducts to install the desired stereochemistry, and subsequent acid-mediated stereoselective cyclization was employed to set up the pyran ring. Finally, the formation of its 2*H*-pyran-2-one ring was achieved by four consecutive steps including the regioselective MgCl_2_-mediated Casnati−Skattebøl *ortho*-formylation of phenol, Wittig methylenation, acryloylation, and Ru(II)-catalyzed ring-closing metathesis. Overall, palodesangrens D dimethyl ether and B trimethyl ether were successfully obtained for the first time via this strategy over nine steps starting from the Diels−Alder reactions [196].

**Scheme 14:**
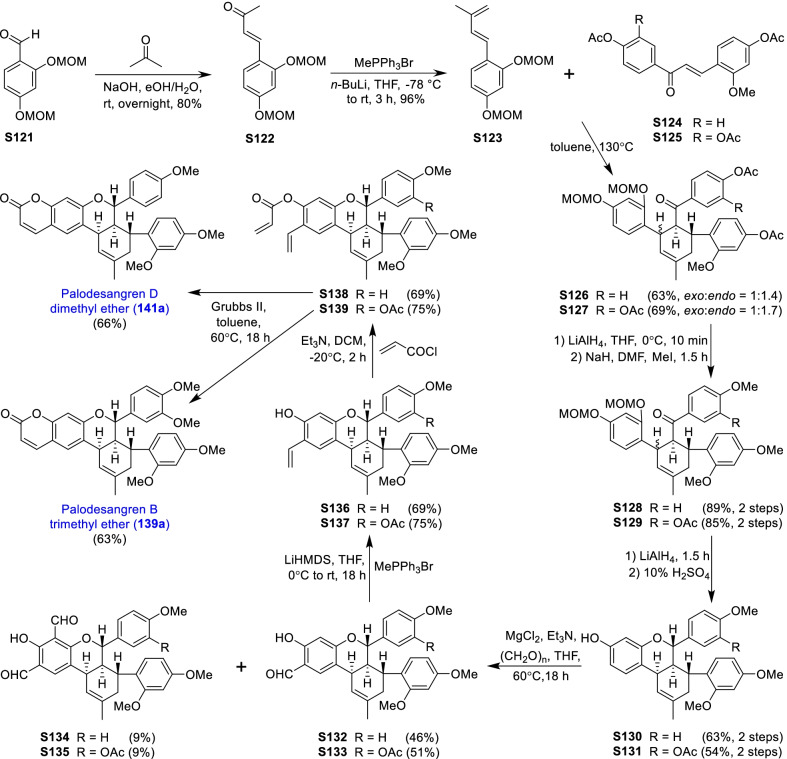
Synthesis of palodesangrens B and D methyl ethers (**139a** and **141a**) by the Ploypradith group

The synthesis commenced with the preparation of aryldiene **S123** from bis-MOM-protected benzaldehyde **S121** by the Claisen − Schmidt condensation and subsequent Wittig methylenation (Scheme 14). With diene **S123** and known chalcone dienophile **S124** in hand, their intermolecular Diels–Alder cycloaddition gave the corresponding cyclohexene **S126** as a mixture of *exo*- and *endo*-adducts with a ratio of 1:1.4 in 63% yield. Subsequent acetyl group deprotection with LiAlH_4_ followed by methylation with MeI provided **S128** in 89% yield over two steps. The ensuing LiAlH_4_-mediated isomerization of the *exo*- and *endo*-adducts mixture **S128** afforded a single *endo* isomer, which was directly subjected to acid-mediated chroman cyclization to furnish the desired tricyclic core **S130** in 63% yield over two steps. After a brief optimization of the Casnati−Skattebøl reaction conditions, the key *ortho*-formylation of **S130** proceeded smoothly to deliver the desired **S132** in 46% yield and a small amount of the byproduct **S134** (9%) together with 29% recovery of **S130**. Next, Wittig methylenation of **S132** with MePPh_3_Br occurred smoothly in the presence of LiHMDS, and the resultant styrene **S136** was subjected to acryloylation to provide the styrene acrylate **S138**. Finally, ring-closing metathesis of compound **S138** using Grubbs II as the catalyst in toluene led to the formation of the desired palodesangren D dimethyl ether (**141a**).

Similarly, palodesangren B trimethyl ether (**139a**) could be readily synthesized from aryldiene **S123** and known chalcone dienophile **S125** by using the same reaction sequences as **141a**. The relative configurations of **139a** and **141a** were the same as those of natrual MDAAs palodesangrens B and D (**139** and **141**).

### Chemical total syntheses of simple or other dehydroprenylphenol type MDAAs (Type H)

#### Total synthesis of ( ±)-sorocenol B

In 2012, the Porco group developed a concise route towards the total synthesis of ( ±)-sorocenol B (**149**). This synthesis featured a silver nanoparticle (AgNP)-catalyzed Diels−Alder cycloaddition to form the cyclohexene core unit and a late-stage Pd(II)-catalyzed oxidative cyclization to install the requisite bicyclo[3.3.1] framework of sorocenol B [197].

**Scheme 15:**
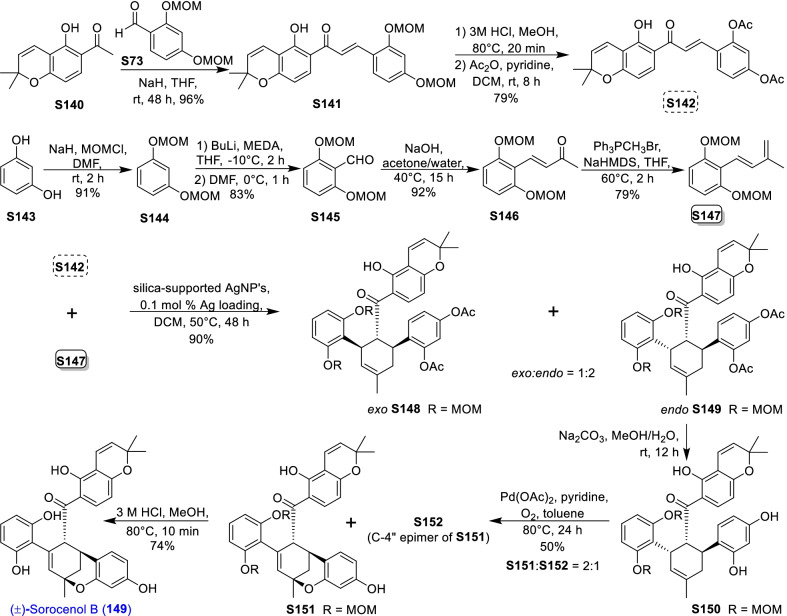
Synthesis of ( ±)-sorocenol B (**149**) by the Porco group

The synthesis started with the preparation of chalcone **S141** from the Claisen−Schmidt condensation between chromene **S140** and benzaldehyde **S73** with NaH as a preferred base in THF (Scheme 15). A hydrolysis of the MOM-protected **S141** delivered a polyphenol, which then gave the acetyl-protected dienophile **S142** under the presence of acetic anhydride. Next, the requisite diene **S147** was prepared in four steps from resorcinol **S143**. After protection of **S143** with MOMCl, the resulting MOM-ether **S144** was subjected to a regioselective formylation to afford benzaldehyde **S145** in 83% yield over two steps. Sequential a base-catalyzed aldol condensation of **S145** with acetone followed by the Wittig olefination yielded the desired diene **S147**. The key Diels−Alder cycloaddition between dienophile **S142** and diene **S147** was then implemented by utilizing silica-supported silver nanoparticles (AgNP’s), which efficiently allowed the formation of the desired cycloadducts *exo*-**S148** and *endo*-**S149** in 90% combined yield with a 1:2 ratio of *exo*/*endo* diastereomers. By unmasking the acetyl-protected phenols of **S149**, the obtained **S150** was submitted to an oxidative Wacker cyclization catalyzed by Pd(OAc)_2_ to construct the bicyclo[1, 3, 3] product. As a result, the desired **S151** and its C-4 epimer **S152** were produced with a ratio of 2:1 in 50% combined yield (Scheme 15). The total synthesis of ( ±)-sorocenol B (**149**) was fulfilled after hydrolysis of **S151** using aqueous HCl in MeOH.

#### Total synthesis of ( ±)-morusalbanol A pentamethyl ether

In 2016, the Chee group described their biomimetic synthesis of morusalbanol A pentamethyl ether (**148a**). The key steps towards the target molecule included a hydrogen-bond-assisted Diels–Alder cycloaddition to build the cyclohexene unit, and a selective cleavage of the *ortho*-methyl ether of the resulting cycloadduct to allow the C3–C21 bond rotation for intramolecular cyclization of the oxabicyclic [3.3.1] core [198]. The construction of this core was based on the previous research on its model by the same group [199].

**Scheme 16:**
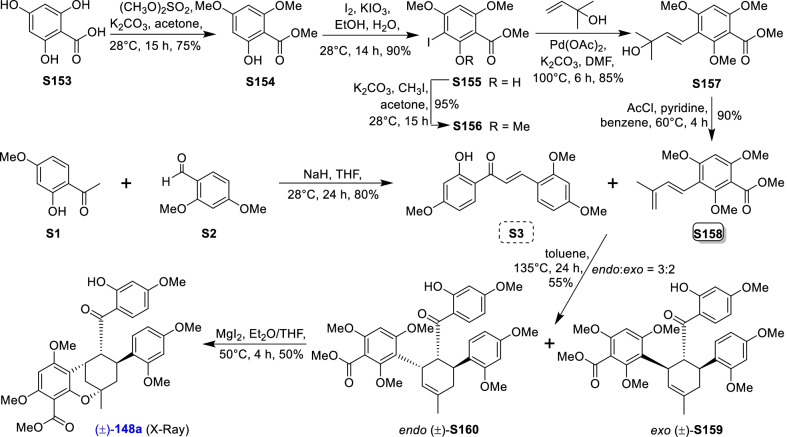
Synthesis of ( ±)-morusalbanol A pentamethyl ether (**148a**) by the Chee group

The synthesis started with commercially available 2,4,6-trihydroxybenzoic acid **S153** that underwent esterification and methyl ether protection to yield ester **S154** in 75% yield (Scheme 16). Regioselective iodination of **S154** followed by methylation of the remaining *ortho*-hydroxy group with iodomethane gave permethylated benzoic ester **S156**. Next, the Heck coupling of **S156** with 2-methylbut-3-en-2-ol proceeded best with Pd(OAc)_2_ as a catalyst to form allylic alcohol **S157** in 85% yield. Upon dehydration of the allylic alcohol with acetyl chloride/pyridine, the desired diene **S158** was obtained in 90% yield. The synthesis of dienophile **S3** in a higher yield was completed by the Claisen–Schmidt condensation of **S1** with **S2** using NaH as the base in THF instead of conventional KOH/MeOH conditions. The thermal Diels–Alder reaction of dienophile **S3** with diene **S158** in toluene at 135 °C afforded the *endo*-adduct **S160** and *exo*-adduct **S159** with a ratio of 3:2 in 55% combined yield. Finally, as shown in Scheme 16, the selective cleavage of the *ortho*-methyl ether of *endo*-**S160** was achieved under the treatment of MgI_2_ and Et_2_O/THF, and subsequent intramolecular cyclization yielded ( ±)-morusalbanol A pentamethyl ether (**148a**) in a stereocontrolled manner in 50% yield.

### Chemoenzymatic total syntheses of MDAAs (Types A − D)

#### MaDA-mediated chemoenzymatic total syntheses of chalcomoracin, 18″-*O*-methychalcomoracin, guangsangon E, kuwanol E, kuwanon J, and deoxyartonin I

In 2020, Lei and coworkers first reported the chemoenzymatic total syntheses of several MDAAs including chalcomoracin (**5**), 18″-*O*-methychalcomoracin (**5b**), guangsangon E (**56**), kuwanol E (**62**), kuwanon J (**81**), and deoxyartonin I (**102**). The synthesis featured an enzymatic intermolecular [4 + 2] cycloaddition of different substrates to produce the natural MDAAs and their derivates with a high efficiency and enantioselectivity. *Morus alba* Diels–Alderase (MaDA), a flavin adenine dinucleotide-dependent enzyme identified from *Morus* cell cultures, was the enzyme functionally responsible for the cycloaddition reaction in the biosynthesis of MDAAs [117].

**Scheme 17:**
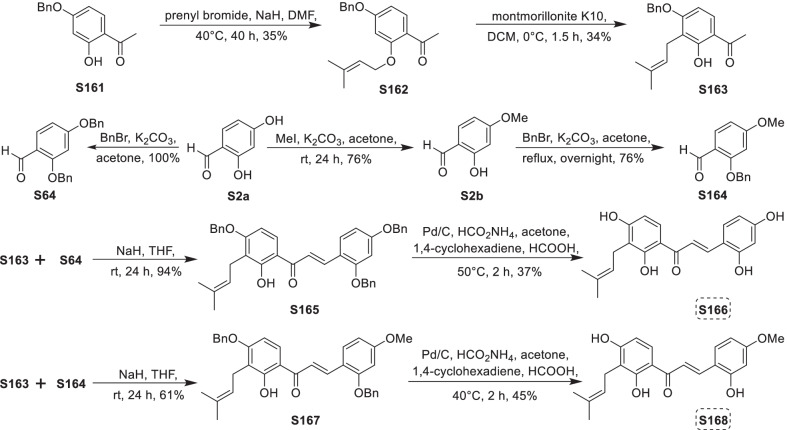
Syntheses of dienophiles (**S166** and **168**) by the Lei group

**Scheme 18:**
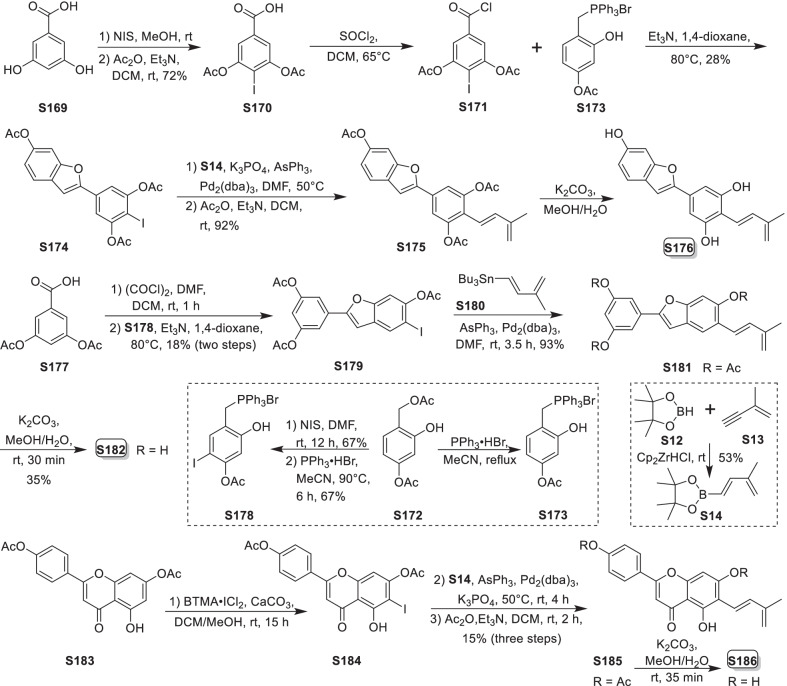
Syntheses of dienes (**S176**, **S182**, and **S186**− **S188**) by the Lei group

**Scheme 19:**
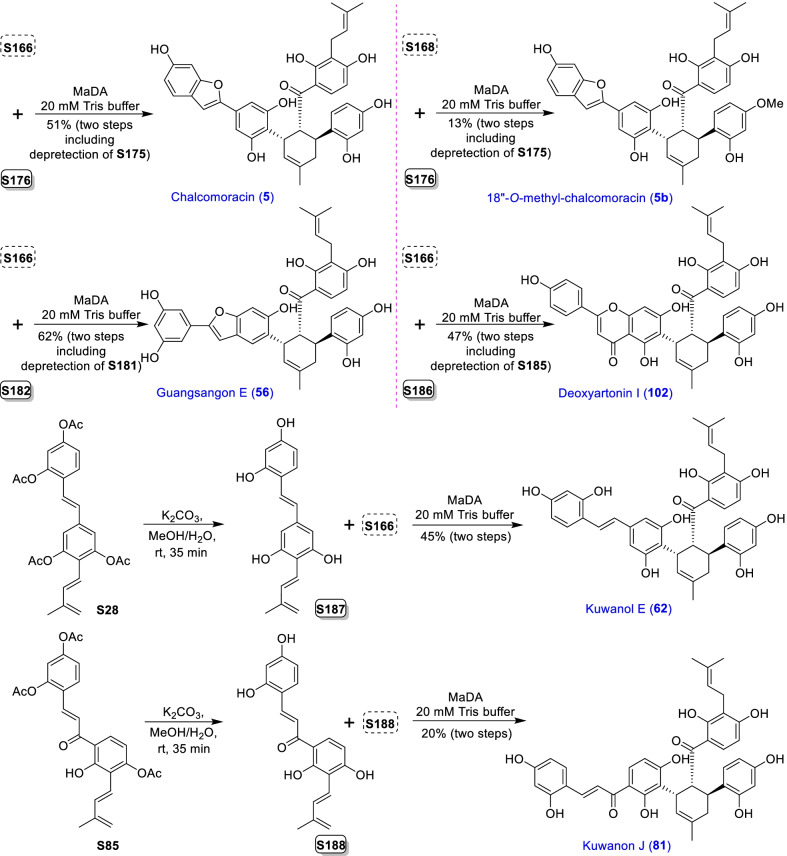
MaDA-mediated chemoenzymatic total syntheses of chalcomoracin (**5**), 18″-*O*-methychalcomoracin (**5b**), guangsangon E (**56**), kuwanol E (**62**), kuwanon J (**81**), and deoxyartonin I (**102**) by the Lei group

As depicted in detail in Schemes 17 and 18, the key building blocks, two main dienophiles (**S166** and **168**) and different dienes (**S176**, **S182**, and **S186** − **S188**), were all obtained by chemical synthesis. For the synthesis of prenyl chalcones morachalcone A (**S166**) and 4′-methylmorachalcone A (**S168**), the intermediate ketone **S163** prepared from **S161** over two steps was subjected to Claisen-Schmidt condensation with aldehydes **S64** and **S164**, respectively, to obtain the benzyl-protected precursors **S165** and **S167**. Benzyl group deprotection finally yielded the two dienophiles **S166** and **168**. As shown in Scheme 18, the phosphonium salts **S173** and **S178** were prepared from the same known compound 4-acetoxy-2-hydroxybenzyl acetate **S172** over one and two steps, respectively. The NIS-mediated iodination of 3,5-dihydroxybenzoic acid **S169** followed by acetyl protection provided iodide **S170** in 72% yield over two steps, which was acylated with thionyl chloride to give **S171**. Next, the preparation of 2-arylbenzofuran iodides (**S174** and **S179**) were achieved by coupling phosphonium salts (**S173** and **S178**) with acyl chlorides (**S171** and the resultant of acylation of **S177** with oxalyl chloride) followed by intramolecular Wittig reaction. Suzuki–Miyaura coupling of 2-arylbenzofuran iodide **S174** with diene borate **S14** followed by an additional acetyl reprotection allowed the formation of acetyl-protected precursor **S175** in excellent yield. While Stille cross-coupling of 2-arylbenzofuran iodide **S179** with the known diene stannane **S180** led to the construction of acetyl-protected precursor **S181** in 93% yield. Finally, global acetyl deprotection of **S175** and **S181** delivered the desired dienes **S176** and **S182**, respectively. After phenol-directed *ortho*-iodination of the known flavone **S183** using BTMA•ICl_2_, the resultant iodide **S184** was subjected to Suzuki–Miyaura coupling with diene borate **S14** followed by a further acetyl reprotection to afford acetyl-protected precursor **S185** in 15% combined yield over three steps. Diene **S186** was prepared from its precursor **S185** by deprotection (Scheme 18), so were dienes **S187** and **S188** from their precursors **S28** and **S85**, respectively (Scheme 19).

As show in Scheme 19, during the final MaDA-mediated chemoenzymatic Diels–Alder cycloaddition, chalcone dienophiles was directly subjected to the corresponding mixture produced by deprotection of diene precursors to yield target MDAAs with high enantioselectivity. Therefore, natural MDAAs and their analogues chalcomoracin (**5**), 18″-*O*-methychalcomoracin (**5b**), guangsangon E (**56**), kuwanol E (**62**), kuwanon J (**81**), and deoxyartonin I (**102**) were obtained from the cycloaddition of **S166** with **S176**, **S168** with **S176**, **S166** with **S182**, **S166** with **S187**, **S188** with **S188**, and **S166** with **S186**, respectively. Their combined yields were 51%, 13%, 62%, 45%, 20%, and 47%, respectively.

#### MaDA-mediated chemoenzymatic total syntheses of artonin I and dideoxyartonin I

**Scheme 20:**
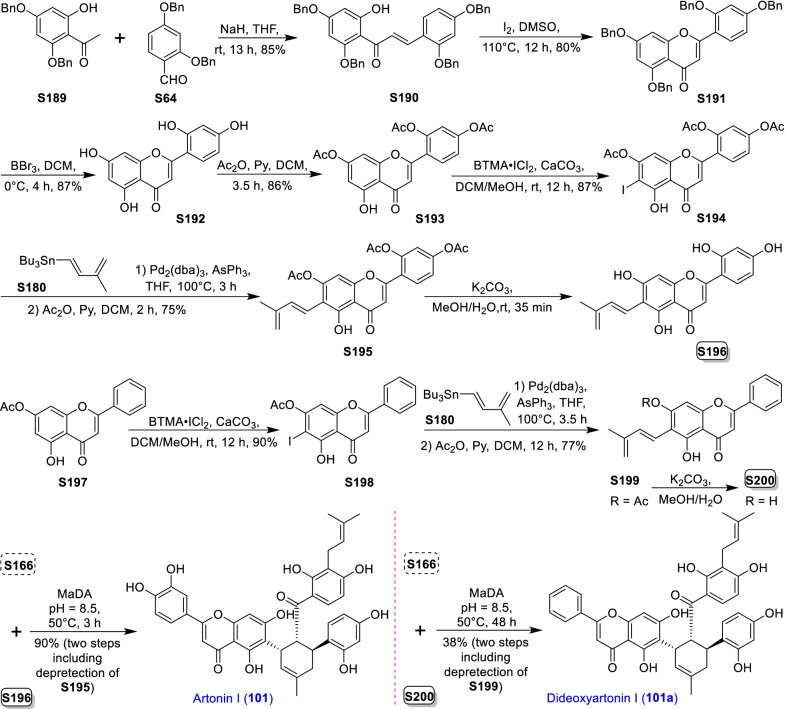
MaDA-mediated chemoenzymatic total syntheses of artonin I (**101**) and dideoxyartonin I (**101a**) by the Lei group

In 2020, the Lei group also completed the chemoenzymatic total syntheses of artonin I (**101**) and dideoxyartonin I (**101a**) with the MaDA catalyzed Diels–Alder reaction as the key step [200]. Based on their previous results, the authors embarked on the preparation of flavonoid dienes. With **S196** as main target diene, the authors started the synthesis from the benzyl-protected **S189** and **S64**. The formation of chalcone **S190** through Claisen–Schmidt condensation reaction of ketone **S189** with aldehyde **S64** followed by iodinemediated oxidative cyclization resulted in flavonoid **S191**, which underwent debenzylation with BBr_3_ to afford flavonoid **S192**, Next, a selective acetylation was performed to yield **S193**, allowing for a phenol-directed *ortho*-iodination using BTMA•ICl_2_. The resulting iodide **S194** (87% yield) was obtained in a regioselective manner. Stille cross-coupling reaction between iodide **S194** and diene stannane **S180** with Pd_2_(dba)_3_ as catalyst in the presence of AsPh_3_ in THF solvent delivered the desired diene precursor **S195**. After mild hydrolysis of **S195**, the product diene **S196** directly underwent Diels–Alder reaction with **S166** under the catalysis of MaDA to yield the natural product artonin I (**101**) with 99% *ee* in 90% yield over two steps (Scheme 20).

Starting from the known flavonoid **S197**, an iodine group was smoothly introduced through phenol-directed *ortho*-iodination. The resulting product **S198** underwent Stille cross-coupling reaction followed by a protection to yield diene precursor **S199**. Diene **S200** was prepared from its precursor **S199** by hydrolysis and then used in the enzymatic Diels–Alder reaction. As expected, MaDA catalyzed the intermolecular [4 + 2] cycloaddition of **S166** with **S200** to afford dideoxyartonin I (**101a**) exclusively (Scheme 20).

## Conclusions

This review has summarized structural classification, distribution, bioactivities, together with chemical and chemoenzymatic total syntheses of naturally occuring MDAAs. MDAAs are a group of structurally unique natural phenolic compounds, which are the most characteristic components of *Morus* plants of the family Moraceae. Over the past four decades, a total of 166 MDAAs including 150 classic (90%) and 16 non-classic (10%) ones have been described, of which dehydroprenyl-2-arylbenzofuran-, dehydroprenylstilbene-, dehydroprenylchalcone-, dehydroprenylflavone-, dehydroprenyldihydroflavone-, dehydroprenylsanggenonflavone-, dehydroprenylcoumarin-, and simple or other dehydroprenylphenol-types, are the eight subtypes (Types A − H) of the classic MDAAs with the same chalcone-skeleton dienophiles. Most MDAAs have many interesting biological properties, especially antineoplastic, anti-inflammation, antimicrobial, antioxidant, and antiviral activities, which may provide some candidates for finding the corresponding innovative drugs. In particular, several compounds such as chalcomoracin, mulberrofurans F, G, J, K, and Q, kuwanons G, H, and L, sanggenons C, D, and G, and albanol B were found to have diverse biological and pharmacological activities, indicating that they have the potential to be developed into multifunctional drugs. Except for the structures of dehydroprenyldihydroflavone type (Type E) and non-classic (Type I) MDAAs, all other types of MDAAs have several examples of chemical or MaDA-mediated chemoenzymatic total syntheses. From all reported synthesis examples, their synthetic strategies included three steps: syntheses of dienophiles, syntheses of dienes, and their biomimetic intermolecular Diels–Alder cycloaddition. Chalcone dienophiles could be obtained directly from known compounds or by a simple synthetic route. The challenge for the synthesis group was to build different diene blocks or to complete the enantioselective biomimetic Diels–Alder cycloaddition. Among them, the enzymatic intermolecular [4 + 2] cycloaddition to produce natural MDAAs was the most efficient and applied strategy with an excellent enantioselectivity and yield. We hope that our newly proposed classification of natural MDAAs and summary of recent total synthetic investigations on them will provide a useful reference for researchers in natural products chemistry and organic synthesis.
